# Cranial and mandibular anatomy of *Plastomenus thomasii* and a new time-tree of trionychid evolution

**DOI:** 10.1186/s13358-023-00267-5

**Published:** 2023-03-16

**Authors:** Serjoscha W. Evers, Kimberley E. J. Chapelle, Walter G. Joyce

**Affiliations:** 1 Department of Geosciences, University of Fribourg, Chemin du Musée 6, 1700 Fribourg, Switzerland; 2 Division of Paleontology, American Museum of Natural History, Central Park West at 79th Street, New York, NY 10024-5192 USA

**Keywords:** Trionychia, Trionychidae, Plastomenidae, Phylogeny, µCT scanning, Evolution, Turtles

## Abstract

Trionychid (softshell) turtles have a peculiar bauplan, which includes shell reductions and cranial elongation. Despite a rich fossil record dating back to the Early Cretaceous, the evolutionary origin of the trionychid bauplan is poorly understood, as even old fossils show great anatomical similarities to extant species. Documenting structural detail of fossil trionychids may help resolve the evolutionary history of the group. Here, we study the cranial and mandibular anatomy of *Plastomenus thomasii* using µCT scanning. *Plastomenus thomasii* belongs to the *Plastomenidae*, a long-lived (Santonian–Eocene) clade with uncertain affinities among trionychid subclades. The skulls of known plastomenids are characterized by unusual features otherwise not known among trionychids, such as extremely elongated, spatulate mandibular symphyses. We use anatomical observations for updated phylogenetic analyses using both parsimony and Bayesian methods. There is strong support across methods for stem-cyclanorbine affinities for plastomenids. The inclusion of stratigraphic data in our Bayesian analysis indicates that a range of Cretaceous Asian fossils including *Perochelys lamadongensis* may be stem-trionychids, suggesting that many features of trionychid anatomy evolved prior to the appearance of the crown group. Divergence time estimates from Bayesian tip-dating for the origin of crown *Trionychia* (134.0 Ma) and *Pan-Trionychidae* (123.8 Ma) constrain the evolutionary time span during which the trionychid bauplan has evolved to a range of < 11 million years. Bayesian rate estimation implies high morphological rates during early softshell turtle evolution. If correct, plastomenids partially fill the stratigraphic gap which results from shallow divergence times of crown cyclanorbines during the late Eocene.

**Supplementary Information:**

The online version contains supplementary material available at 10.1186/s13358-023-00267-5.

## Introduction

*Trionychidae* (i.e., crown-group softshell turtles; Joyce et al., 2021) is one of the major clades of living turtles. They are remarkable for their highly derived body plan among turtles, which notably includes strong reductions of the shell (e.g., Meylan, 1987), high levels of adaptation to aquatic locomotion in the limbs (e.g., Joyce & Gauthier, 2004; Pace et al., 2001), and craniocervical adaptations to subaqueous hunting (e.g., Hermanson et al., 2022; Pritchard, 1984). Trionychids have high size disparity among extant species (Farina et al., 2022; Pritchard, 2001) and are today biogeographically wide spread, mostly across Africa, Asia, and North America (e.g., Ernst & Barbour, 1989; TTWG, 2021). Trionychids are an ancient clade, with molecular divergence time estimates proposing a late Early Cretaceous (e.g., Joyce et al., 2013; Pereira et al., 2017) or early Late Cretaceous (Thomson et al., 2021) diversification. The two major extant lineages of soft-shelled turtles, *Cyclanorbinae* and *Trionychinae*, on the other hand, have much shallower origination estimates, ranging from the early Eocene (Pereira et al., 2017) to the Oligocene (Thomson et al., 2021) for cyclanorbines and from the Late Cretaceous (Pereira et al., 2017) to the Paleocene (Thomson et al., 2021) for trionychines.

The evolutionary history of trionychids is difficult to reconstruct. This is due in part to uneven sampling in the fossil record (Georgalis & Joyce, 2017; Vitek & Joyce, 2015), but also a paucity of phylogenetically informative characters combined with high levels of homoplasy (Meylan, 1987). As a result, it is difficult to even establish fossils as phylogenetically belonging to the trionychid crown group (i.e., *Trionychidae*) or stem group (i.e., non-trionychid *Pan-Trionychidae*). This is maybe best exemplified by the Early Cretaceous *Perochelys lamadongensis*, which is one of the oldest known turtles with a trionychid-like bauplan, but has been found in equally parsimonious phylogenetic positions on the stem lineage of trionychids and deeply nested among the crown group as the sister taxon to individual living species (Li et al., 2015). As a result, the fossil record of pan-trionychids (i.e., total-group trionychids) includes many Cretaceous species (e.g., Danilov & Vitek, 2013; Danilov et al., 2014; Georgalis & Joyce, 2017; Li et al., 2015; Vitek & Danilov, 2013; Vitek & Joyce, 2015), for which it is mostly unclear if they represent stem-trionychids, stem-cyclanorbines, or stem-trionychines (e.g., Brinkman et al., 2017; Li et al., 2015). Alternative phylogenetic placements of many fossil species or lineages, however, is expected to have an impact on our understanding of the biogeographic evolution of soft-shelled turtles (e.g., Joyce & Lyson, 2010), diversification rates and times (e.g., Joyce et al., 2013), as well as character evolution (e.g., Brinkman et al., 2017). Phylogenetic methods that take auxiliary information such as stratigraphic ages of fossils into account may prove useful to resolving trionychid evolution.

A particularly intriguing fossil softshell clade is *Plastomenidae*. Plastomenids are a relatively species rich clade of pan-trionychids (e.g., Edgar et al., 2021; Hay, 1908; Hutchison, 2009; Jasinski et al., 2022; Joyce & Lyson, 2011; Joyce et al., 2009, 2018; Lyson et al., 2021) that are exclusively known from the Santonian to Eocene of North America (Joyce & Lyson, 2010; Vitek & Joyce, 2015; Georgalis & Joyce, 2017; Edgar et al., 2021; Jasinski et al., 2022). Most plastomenid species are known from incomplete shell material (e.g., Vitek & Joyce, 2015), with the notable exception of the Late Cretaceous (Maastrichtian) *Gilmoremys lancensis* and the Early Eocene (Bridgerian) *Plastomenus thomasii*, for which shells are known in addition to skulls (Joyce & Lyson, 2011; Joyce et al., 2016; Vitek & Joyce, 2015). The latter species was originally described based on fragmentary remains (Cope, 1872; see Vitek & Joyce, 2015 for list of syntypes) to which Hay (1908) referred two beautifully preserved specimens, in particular AMNH FR 6015 (Gaffney, 1979; Hay, 1908), which includes some shell remains in addition to a beautifully preserved cranium and mandible, and AMNH FR 6018, a nearly complete shell (see Joyce & Lyson, 2010 for a full summary of the taxonomic history). This referral has generally been accepted in the literature (e.g., Gaffney, 1979; Hummel, 1929), but is not supported using modern taxonomic standards, as the holotype is undiagnostic (Lyson & Joyce, 2010; Vitek & Joyce, 2015). Joyce and Lyson (2010) announced that they were preparing a formal ICZN appeal to replace the non-diagnostic holotype of *Plastomenus thomasii* with AMNH FR 6018. This is still pending (e.g., Vitek & Joyce, 2015). Here, we follow the apparent consensus between studies (e.g., Gaffney, 1979; Hay, 1908; Loyce & Lyson, 2010; Vitek & Joyce, 2015) in accepting the taxonomic referral of AMNH FR 6018 and AMNH FR 6015 to *Plastomenus thomasii*, despite the current formal lack of a neotype.

AMNH FR 6015 and AMNH FR 6018 have been used as the hypodigm for the phylogenetic characterization of *Plastomenus thomasii*. Joyce and Lyson (2010) retrieved *Plastomenus thomasii* (and, by extension, the clade *Plastomenidae*) as stem cyclanorbines in their expansion of Meylan’s (1987) matrix that was phylogenetically constrained to a molecular backbone. Joyce et al. (2009) and Joyce and Lyson (2011) corroborated these results in slightly modified versions of Meylan’s (1987) matrix and using more plastomenid taxa. Later variations of the same matrix have, however, also found *Plastomenidae* to be in a polytomy with cyclanorbines and trionychines (Joyce et al., 2016; Lyson et al., 2021), or as stem trionychines (Joyce & Lyson, 2017; Joyce et al., 2018; see also equal weighting results in Brinkman et al., 2017). Some of the most recent iterations of this matrix recovered the clade once again on the stem of cyclanorbines (Edgar et al., 2021; Jasinski et al., 2022). Therefore, the phylogenetic position of plastomenids is in constant flux and seems to be sensitive to slight variations in taxon and character sampling, as well as to character weighting strategies (e.g., Brinkman et al., 2017).

Although the skull of AMNH FR 6015 has been known for more than 100 years, it has not been properly described. Hay (1908) provided sketches and some overview statements of the fossil, which was not fully prepared at the time. Gaffney (1979) then provided an idealized line-drawing of the skull, which included the previously unprepared palate, but provided no description. Our initial observations on the skull indicated several differences to Gaffney’s (1979) reconstruction. This reconstruction is in part the basis for previous phylogenetic scorings for *Plastomenus thomasii* (Joyce & Lyson, 2010). The primary goal of this contribution is a detailed anatomical description of AMNH FR 6015 based on digital dissection of micro-computed tomography (µCT) scans. A secondary goal is to update the phylogenetic scoring of skull characters for the species. We also use our anatomical observations to provide a new phylogenetic analysis of softshell turtles that includes novel characters and modified character state observations in an attempt to better clarify the phylogenetic position of *Plastomenidae* among *Pan-Trionychidae*. For this, we use standard parsimony protocols as well as a Bayesian tip-dating approach that takes the stratigraphic position of fossils into account.

## Methods

### Digital anatomy

**Fig. 1:**
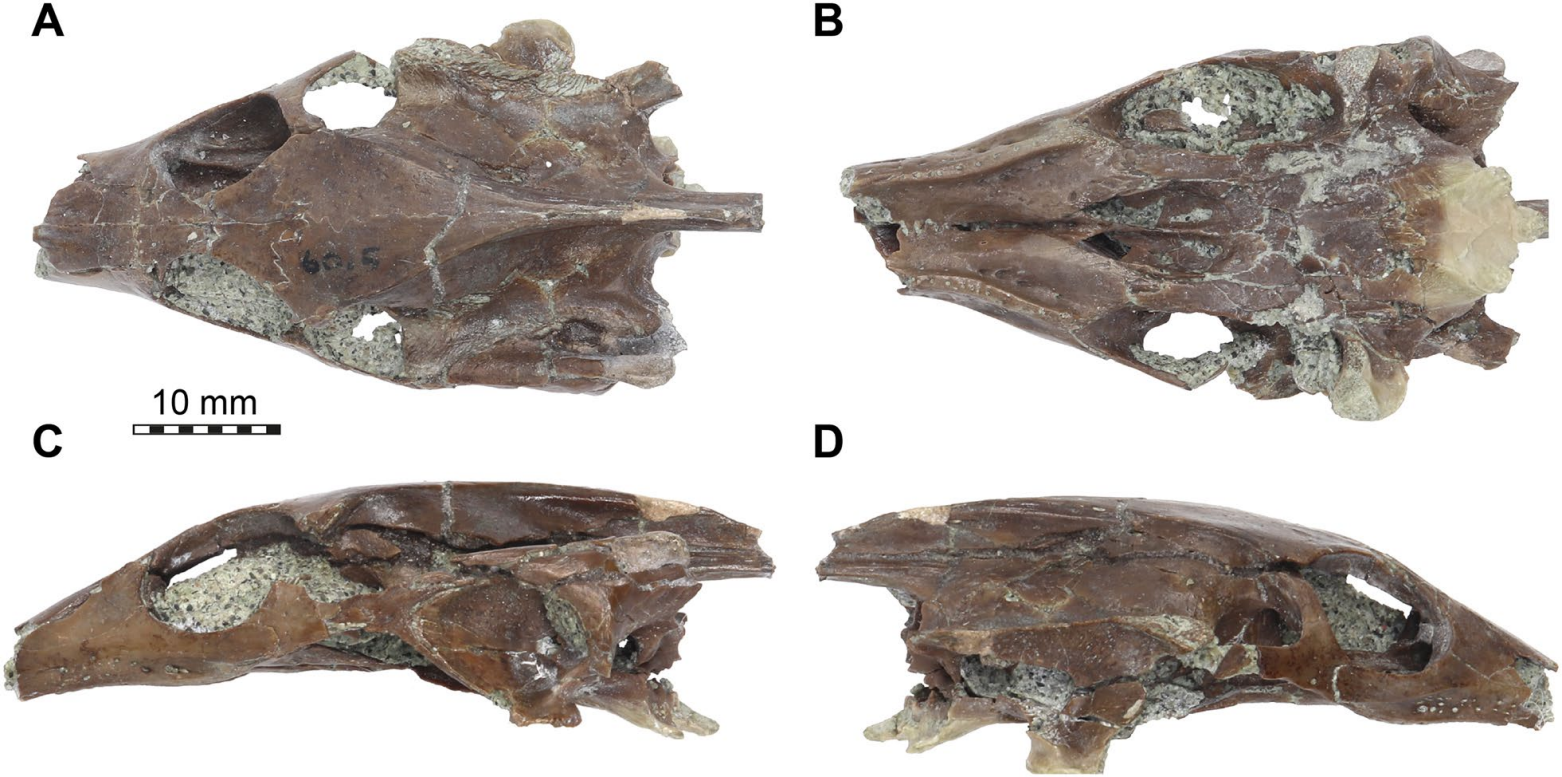
Photographs of the cranium of *Plastomenus thomasii* (AMNH FR 6015). **A** dorsal view. **B** ventral view. **C** left lateral view. **D** right lateral view

**Fig. 2:**
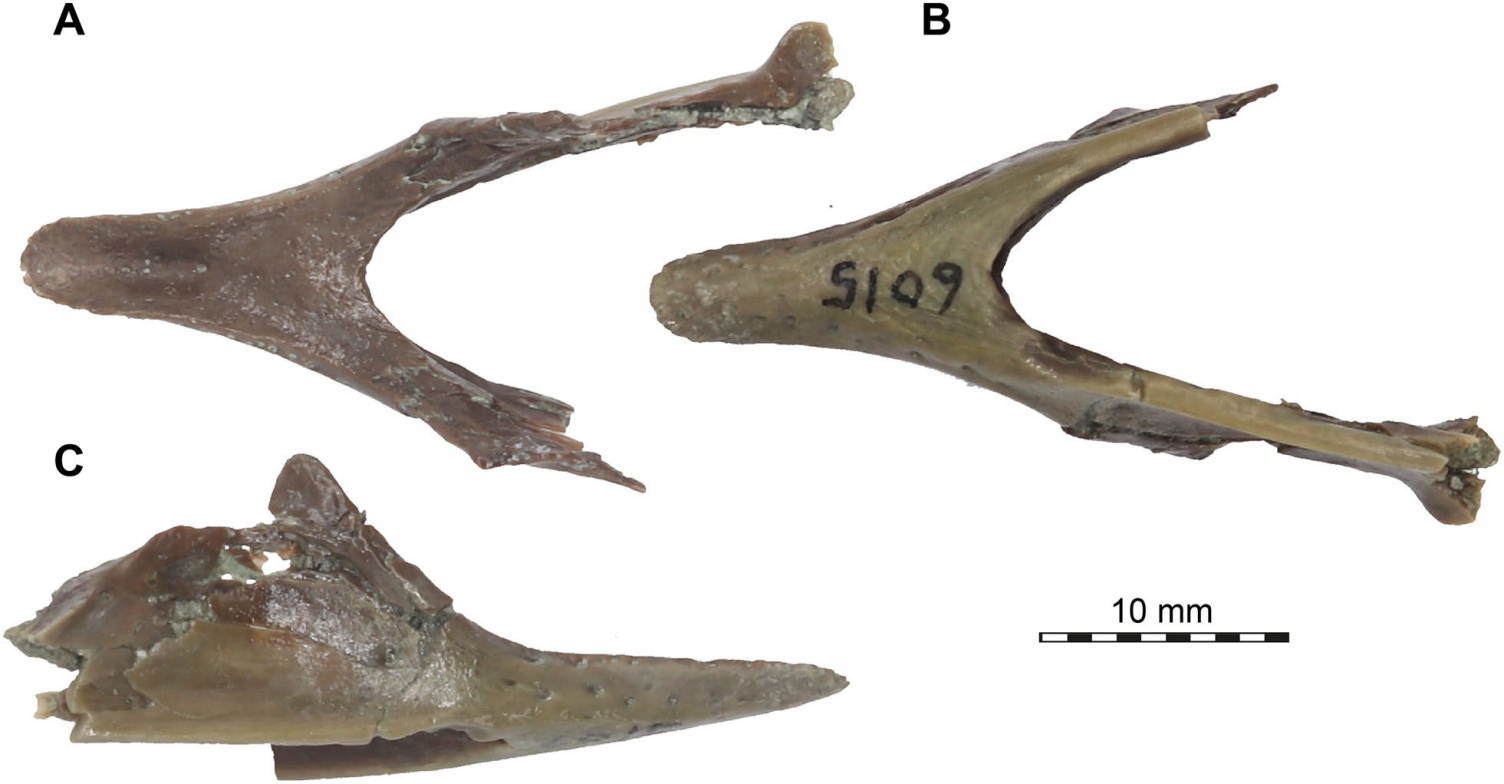
Photographs of the mandible of *Plastomenus thomasii* (AMNH FR 6015). **A** dorsal view. **B** ventral view. **C** right lateral view. Note that the posterior part of the left mandibular ramus is preserved, but broken from the main part of the fossil. See 3D renderings for complete fossil

µCT scanning of AMNH FR 6015 (Figs. 1, 2) was undertaken by Morgan Chase and James Napoli at the American Museum of Natural History (AMNH) Microscopy and Imaging Facility, using a GE phoenix v|tome|x s240 scanner. Two µCT scans were obtained: a cranium-only scan (voltage of 140 kV, current of 130 uA, exposure time of 500 ms, VoxelSize = 18.30829 µm) and a cranium with mandible scan (150 kV, 150 uA, 400 ms, VoxelSize = 25.12641 µm). The cranial bones were digitally segmented by SWE from the first scan using Materialise Mimics software v.24 and the mandibular bones were digitally segmented by KEJC from the second scan using VGSTUDIO MAX v. 3.2.

Mandibular parts were digitally re-assembled by KEJC in VGSTUDIO MAX v. 3.2, and figures were rendered in Blender v. 2.79b by SWE.

We deposited µCT scans and 3D models of AMNH FR 6015 on the online data repository MorphoSource (Evers & Chapelle, 2022; https://www.morphosource.org/projects/000451076), with the scanning parameters specified for each scan. The AMNH has a download policy for material under their care that requires a download permission request by the user, stating the academic purpose for the request.

### Specimen photography

AMNH FR 6015 was photographed using a Canon EOS 77D with a Canon 24–105 mm lens by KEJC. Photographs were edited in GIMP 2.10.32 by KEJC.

### Comparative anatomy

For comparisons, we used µCT-derived cranial models of several extant trionychids, in particular the trionychines *Pelodiscus sinensis* (IW 576-2), *Amyda cartilaginea* (FMNH 224117), *Apalone spinifera* (NHMUK 65.5.9.21), and *Chitra chitra* (NHMUK 1936.12.16.1) and the cyclanorbines *Lissemys punctata* (SMF 74141) and *Cyclanorbis senegalensis* (NHMUK 65.5.9.21). The comparative µCT scans and 3D skull models were all published previously on MorphoSource, including a bone-by-bone segmentation of *Apalone spinifera* (Evers & Benson, 2018). Unless otherwise stated, comparative statements are based on these specimens. Anatomical nomenclature broadly follows Gaffney (1972, 1979), Evers et al., (2022a) for the mandible, Albrecht (1967) for details of the infraorbital artery pattern, and Rollot et al., (2021a) for carotid and facial nerve foramina and canals.

### Phylogenetic matrix

For our phylogenetic work, we used the phylogenetic matrix of Lyson et al., (2021; 95 characters, 39 taxa) as our basis. We added the Cretaceous trionychid *Axestemys infernalis* to the dataset, so that our matrix has 40 terminal taxa. Our new observations of the cranial morphology of *Plastomenus thomasii* revealed variation that warranted the addition of 26 new phylogenetic characters. Of these, 18 are cranial characters and 8 are mandibular characters, which were taken and/or modified from previous phylogenetic matrices (Anquetin et al., 2015; Brinkman & Wu, 1999; Evers et al., 2019a, 2022a; Hirayama, 1985; Joyce, 2007; Meylan, 1996; Vlachos & Rabi, 2018; Zhou & Rabi, 2015) or newly conceived for this study (see character list in the Additional file 1: Text). We deleted four characters that were in the original character matrix (characters 38, 56, 84, 88 of Lyson et al., 2021), and slightly modified the state definitions of three additional characters. All changes and deletions are justified in the remarks section of each character (see Additional file 1: Text). The new total number of characters is 116. Due to the deletion of characters, our character numbers differ from those of Lyson et al. (2021), but the original character numbers are given in our character list for cross-reference (see Additional file 1: Text). We revised the scorings of 19 characters for *Plastomenus thomasii* based on the new observations provided herein: ch. 28: 1- > 2; ch. 29: 1- > 2; ch. 30: ?- > 1; ch. 31: ?- > 3; ch. 32: ?- > 1; ch. 33: 1- > 2; ch. 35: inapplicable- > 2; ch. 37: 2-> 3; ch. 41: 2- > 1; ch. 44: 1- > ?; ch. 47:?- > 1; ch. 48:?- > 2; ch. 49:?- > inapplicable; ch. 50: ?- > 3; ch. 51:?- > 1; ch. 52:?- > 3; ch. 53: inapplicable-> 1; ch. 85:?- > 1; ch. 86: ?- > inapplicable. Of these changes, ten are from “?” to a positive score, reflecting additional detail gained through our re-examinations, while the remaining nine changes are related to previous scorings we determine to have been incorrect. We also adjusted some scorings for *Plastomenus joycei* (ch. 80: 1- > ?; ch. 81: 0- > ?; ch. 82: 1- > ?; ch. 89: 1- > 0; ch. 91: 1- > 0) and *Gilmoremys lancensis* (ch. 30: 2- > 1; ch. 50: 2- > 3; ch. 83: 1- > 0&1). *Nemegtemys conflata*, known from fragmentary hyo- and hypoplastra, was previously scored for several carapacial and other characters (Lyson et al., 2021) that are not observable from the published material (Danilov et al., 2014). Thus, we modified three scorings for this taxon: ch.13: 2- > ?; ch. 90: 0- > ?; ch. 91: 0- > ?. This leaves this species with only two positively scored characters. Although we left the species in the matrix, it was excluded from all phylogenetic analyses, following safe taxonomic reduction criteria (Wilkinson, 1995). For character 42 (character 43 of Lyson et al., 2021), all trionychids were previously scored incorrectly, as the scorings were reversed (i.e., taxa with an exoccipital-pterygoid contact were scored without one, and vice versa). We reversed the scorings to correct the error. The character-taxon matrix is provided as Additional file 2: Data 1.

### Parsimony analysis

We analyzed the character-taxon matrix in TNT 1.5 (Goloboff & Catalano, 2016) using a New Technology Search with the initial level set to 30 and finding the minimum length 30 times. All tree search algorithms were enabled, and we enforced a backbone constraint fixing the topology of extant taxa according to their phylogenetic interrelationships (following Thomson et al., 2021). Characters 1, 3, 5, 16, 18–20, 22, 31, 40, 44, 53–54, 59, 77, 102, 108–110 were treated as ordered morphoclines, and *Adocus lineolatus* was used as the outgroup taxon. After initial analyses, we added an additional round of TBR branch swapping. This analysis was repeated twice, under equal weighting and implied weighting using a concavity constant of K = 12, following recommendations by Goloboff et al. (2018). We provide a.tnt file as Additional file 3: Data 2, which also stores the molecular backbone topology.

### Bayesian analysis

We also ran a Bayesian tip-dating analysis in MrBayes 3.2.6 (Ronquist et al., 2009). We used the same character ordering as in the parsimony analysis and used *Adocus lineolatus* as the outgroup taxon. In addition to including a phylogenetic backbone constraint, we included an ingroup constraint to the exclusion of *Adocus lineolatus* and *Carettochelys insculpta*. For the substitution model, we used the Mkv-model of Lewis (2001), which models equal state frequencies, and includes an ascertainment bias correction for morphological data, counteracting the overestimation of evolutionary rates. Rate variation across characters in the model is drawn from a gamma distribution. For the clock model, we specified a relaxed-clock that allows rates to vary over time with each branch having an independent rate drawn from a gamma distribution (independent gamma rate, or IGR; Lepage et al., 2007). The gamma distribution was set to have a variance following an exp (10) distribution (Zhang, 2016). For the clock rate prior, we used a normal distribution with a mean of 0.001 and a variance of 0.1 This accounts for uncertainty in the clock rate: using a variance higher than the mean establishes a wide prior distribution of clock-rates to sample from, and values with a small standard deviation could bias the analysis toward the prior settings on the clockrate (Ezcurra et al., 2020). We used fixed age priors of zero for extant taxa, whereas uniform age priors modelled around the first and last appearance dates were used for fossils, which were treated as tips. The tree model was implemented as a fossilized-birth–death (FBD) model (Heath et al., 2014; Stadler, 2010), for which we used default priors for the parameterization of speciation, extinction and fossilization rates. A diversified sampling strategy was specified (Zhang et al., 2016), as our taxon sample covers all major clades of trionychids. The extant sampling proportion was set to be 0.571, as our sample includes 20 out of 35 extant species of Trionychia (TTWG, 2021). We specified the root age prior using an offset exponential function. The exponential decay function is shaped by the mean value, and the likelihood of the root age decreases exponentially from the mean (Zhang, 2016). The mean of the function was set to be 175 Ma, and the minimum was set to be 130 Ma. These were modelled after molecular divergence time estimates for the crown group of *Trionychia* (Thomson et al., 2021). To estimate the posterior distribution, we used metropolis-coupled Markov chain Monte Carlo (MCMCMC) algorithms with two independent runs and four chains. We modified the chain temperature from the default coefficient of 0.1 to 0.05 and set the number of attempted chain swaps to be 3 (default is 1), as an initial run of the analysis with default parameters showed only moderate mixing behavior at 24–40% accepted state changes between chains. Samples from the posterior were recorded every 500^th^ generation, at which point the chains attempted a chain swap. In an initial analysis, Markov chains were set to run for 50.000.000 generations and additional generations in batches of 10.000.000 were appended until the estimated sample sizes (EES) for all parameters exceeded 200 in each run individually, which was taken as parameter convergence (Rambaut et al., 2018), and for which the initial run of the analysis required 100.000.000 generations. The changed heating and chain swap parameters resulted in better mixing with acceptance rates for chain swaps of 55–60% successful state exchanges between adjacent chains. Convergence was also achieved earlier, so that the final analysis presented herein required only 50.000.000 generations. At this point EES values for all parameters comfortably exceeded 200 in each run individually. Topological convergence between runs was indicated by the average deviation of split frequencies dropping below 0.01, with a finishing value of 0.004 after 50.000.000 generations. The trace plot of the number of generations against the log probability of the data given by the sump command of MrBayes indicated convergence on stationarity as well. Similar between and within run variances of posterior samples were also achieved, as indicated by the Potential Scale Reduction Factors reaching a value of 1 (Gelman & Rubin, 1992; Ronquist et al., 2009). The MrBayes script is provided as Additional file 4: Data 3, and also includes the fossil ages used.

### Character optimization

In order to retrieve the synapomorphies that support various clades of our phylogenetic results, we performed an optimization in Paup* (Swofford, 2002). This was done because TNT only retrieves unambiguous synapomorphies, i.e., those character state transitions that can be determined with certainty given the data and tree topology. In datasets that include unknown data or sister-taxa with differing scorings, some character state transitions cannot be projected with certainty to a specific node, resulting in ambiguous optimizations. These, however, can still provide important evolutionary information, as the possibility of transitions is usually limited to a specific part of the tree. We thus optimized character states according to two criteria, which determine ambiguous character state transitions to occur in the earliest possible node (accelerated transformation = ACCTRAN) or in the latest possible node (delayed transformation = DELTRAN). Justification for this procedure over the more commonly used listing of only unambiguous character transitions as returned by TNT is best illustrated with an example. In the phylogenetic results of Lyson et al. (2021), *Plastomenus* spp. and *Gilmoremys* spp. form a sister-group and are part of a larger plastomenid clade. Two (hypothesized) skull features exclusively known in *Plastomenus thomasii* and *Gilmoremys lancensis* (i.e., the only plastomenids with known skull material) could be optimized as a synapomorphy of their exclusive clade under DELTRAN, or as a synapomorphy for *Plastomenidae* under ACCTRAN, whereas an unambiguous-only list would exclude the character altogether. As optimizations should be performed on a fully bifurcated tree, we chose a tree with such properties from our results. We decided to use the maximum clade credibility (MCC) tree from our Bayesian analysis, as justified in the results section.

### Comparing parsimony and Bayesian topology

In order to test if our Bayesian-based topological results could realistically also be retrieved using parsimony methods, we compared the topological results of the MCC tree with a most parsimonious tree (MPT) from our parsimony analysis using equal weighting (from which the first MPT was chosen). For the topological comparison, we used Templeton’s test (Templeton, 1983) as implemented in PAUP*. We used two-tailed p-values for the test, as we did not want to hypothesize that one tree is superior to the other, and instead were interested in whether they are significantly statistically different with regard to their tree lengths.

*Institutional abbreviations:* AMNH = American Museum of Natural History, New York, USA; IW = University of Tübingen, Ingmar Werneburg lab collection, Tübingen, Germany; FMNH = Field Museum of Natural History, Chicago, USA; NHMUK = Natural History Museum, London, United Kingdom; SMF = Senckenberg Museum Frankfurt; USNM = National Museum of Natural History,

Smithsonian Institution, Washington, DC, USA.

## Systematic palaeontology

*Pan-Trionychidae* Joyce et al., 2021

*Plastomenidae* Hay, 1908 (sensu Joyce et al., 2021).

*Plastomenus thomasii* (Cope, 1872).

Syntype series: AMNH 3948, USNM 4092, 4093, 5838, shell fragments (Cope, 1884, p. 18.2–8).

Type locality: Bridger Basin; Bridger Formation, Bridgerian NALMA, Ypresian–Lutetian, Eocene (Hay, 1908; Vitek & Joyce, 2015).

Amended diagnosis: *Plastomenus thomasii* can be diagnosed as a member of *Pan-Trionychidae* by a reduced quadratojugal that does not contact the postorbital or maxilla; a V-shaped entoplastron; the absence of scutes and peripherals; and the presence of sculpturing that covers all metaplastic portions of the shell bones. *Plastomenus thomasii* can be diagnosed as an unambiguous member of *Plastomenidae* (i.e., *Gilmoremys* and *Plastomenus* spp.) by the presence of a strongly emarginated dorsal edge of the external naris; a jugal-squamosal contact; a jugal-parietal contact; a division of the foramen palatinum posterius into two smaller foramina; a fenestra postotica, which is partially subdivided by the exoccipital; an epipterygoid-prootic contact anterior to trigeminal foramen in the majority of specimens; an extensive secondary palate formed by the infolded maxillae; vomerine foramina; an extremely elongated mandibular symphysis; a strong expansion of the lingual margin of the dentary forming a spatulate symphyseal area; an enlarged foramen dentofaciale majus; a dorsal surangular foramen; carapacial striations in adults; a preneural; I-shaped epiplastra; and xiphiplastra that contact each other along their entire length. *Plastomenus thomasii* is currently differentiated from other unambiguous members of Plastomenidae by the complete reduction of the postorbital; a postorbital bar less than one-fifth of orbit diameter; the complete fusion of the frontals; the absence of accessory ridges of the upper triturating surfaces; the presence of a massive infolding ridge of the quadrate; a trionychid surface pattern that becomes smooth towards the center of the carapacial and plastral disk; costals VIII that are longer than wide (also present in *P. joycei*); a dorsal rim of the costals that splits into separately protruding dorsal and visceral portions; hyo-, hypo- and xiphiplastra that contact one another fully along the mid-line; and the presence of metaplastic ossification that rolls onto the posterior aspects of the lateral process of the hypoplastron.

## Description

**Fig. 3:**
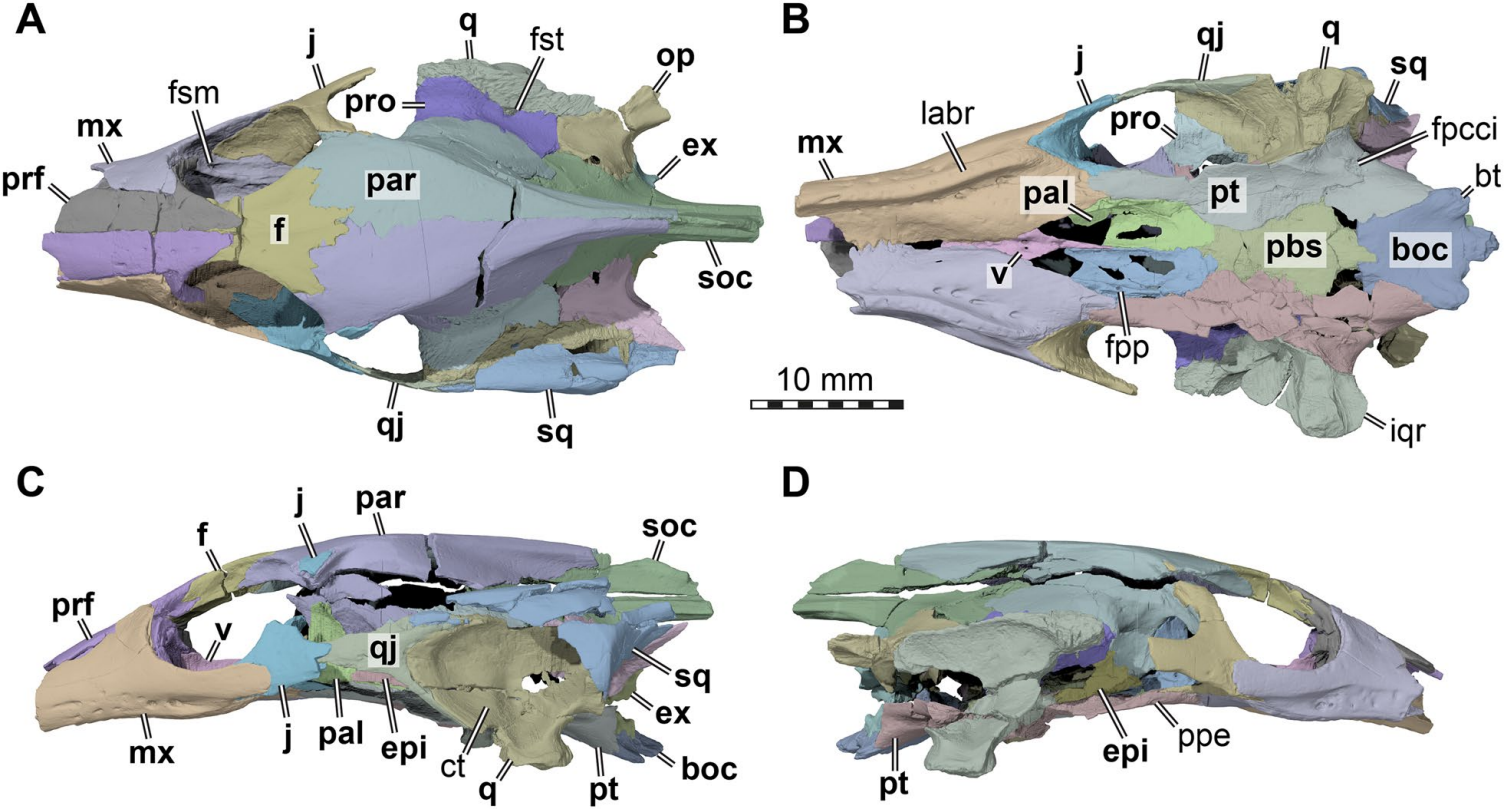
Three dimensional renderings of the cranium of *Plastomenus thomasii* (AMNH FR 6015). **A** dorsal view; **B** ventral view; **C** left lateral view; **D** right lateral view. Note that bones are labelled in bold. *boc* basioccipital, *bt* basi tuber, *ct* cavum tympani, *epi* epipterygoid, *ex* exoccipital, *f* frontals (fused), *fpcci* foramen posterius canalis carotici interni, *fpp* foramen palatinum posterius, *fsm* foramen supramaxillare, *fst* foramen stapedio-temporale, *iqr* infolding quadrate ridge, *j* jugal, *labr* labial ridge, *mx* maxilla, *op* opisthotic, *pal* palatine, *par* parietal, *pbs* parabasisphenoid, *ppe* processus pterygoideus externus, *prf* prefrontal, *pro* prootic, *pt* pterygoid, *q* quadrate, *qj* quadratojugal, *soc* supraoccipital, *sq* squamosal, *v* vomer

**Fig. 4:**
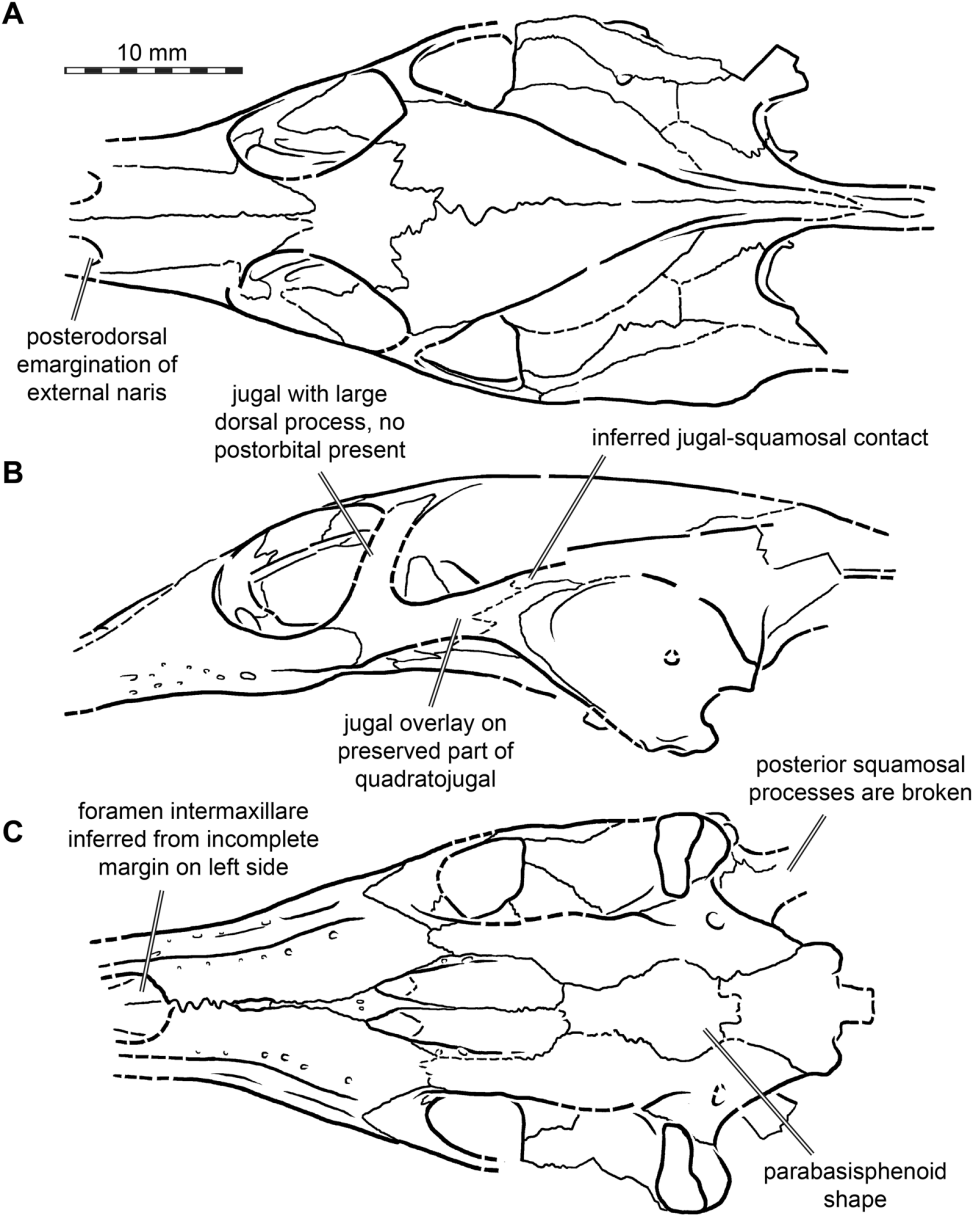
Interpretative line drawings of the cranium of *Plastomenus thomasii* (AMNH FR 6015). **A** dorsal view. **B** left lateral view. **C** ventral view. Drawings are based on renderings in Fig. 3. Labels indicate incompletely preserved features and our interpretation thereof

**Fig. 5:**
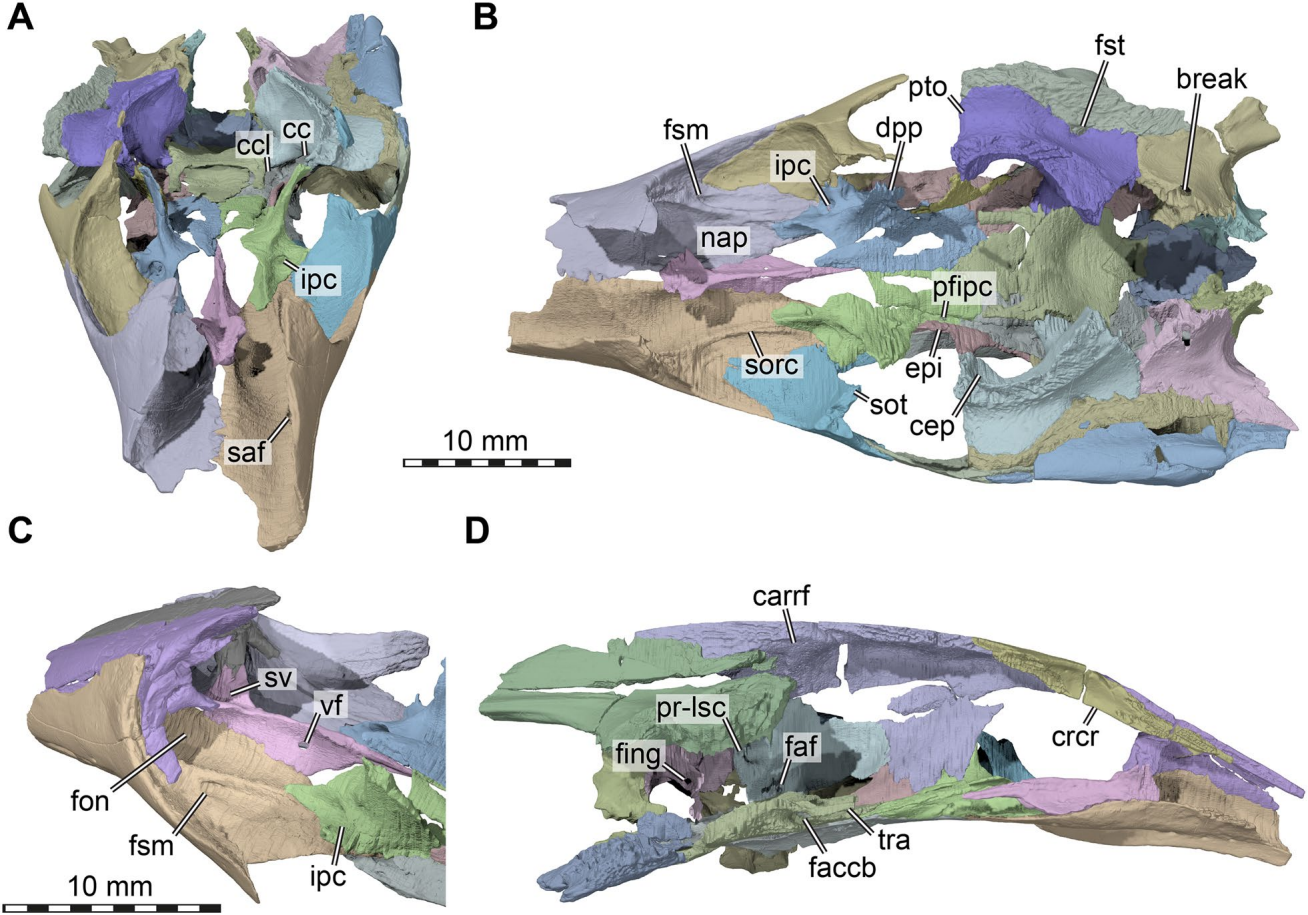
Three dimensional renderings of the interior of the cranium of *Plastomenus thomasii* (AMNH FR 6015). **A** anterodorsal view with skull roof elements removed; **B** dorsal view with skull roof elements removes. Note symmetrical breakage in both opisthotics into the underlying endosseous labyrinth; **C** posterolateral view into anterior parts of the left orbital fossa; **D** medial view onto left side of skull. *carrf* cartilaginous rider fossa, *cc* canalis cavernosus, *ccb* canalis caroticus basisphenoidalis, *cep* cavum epiptericum, *crc*r crista cranii, *dpp* dorsal palatine process, *epi* epipterygoid, *faccb* foramen anterius canalis carotici basisphenoidalis, *faf* fossa acustico-facialis, *fing* foramen internum nervi glossopharyngei *fon* foramen orbito-nasale, *fsm* foramen supramaxillare, *fst* foramen stapedio-temporale, *ipc* intrapalatine canal, *nap* narial passage, *pfipc* posterior foramen into intraplatine canal (for vidian nerve), *pr-lsc* prootic bar of bone contributing to the lateral semicircular canal, *pto* processus trochlearius oticum, *saf* supraalveolar fenestra, *sorc* suborbital crest, *sot* septum orbitotemporale, *sv* sulcus vomeri, *tra* trabecula, *vf* vomer foramen

**Fig. 6:**
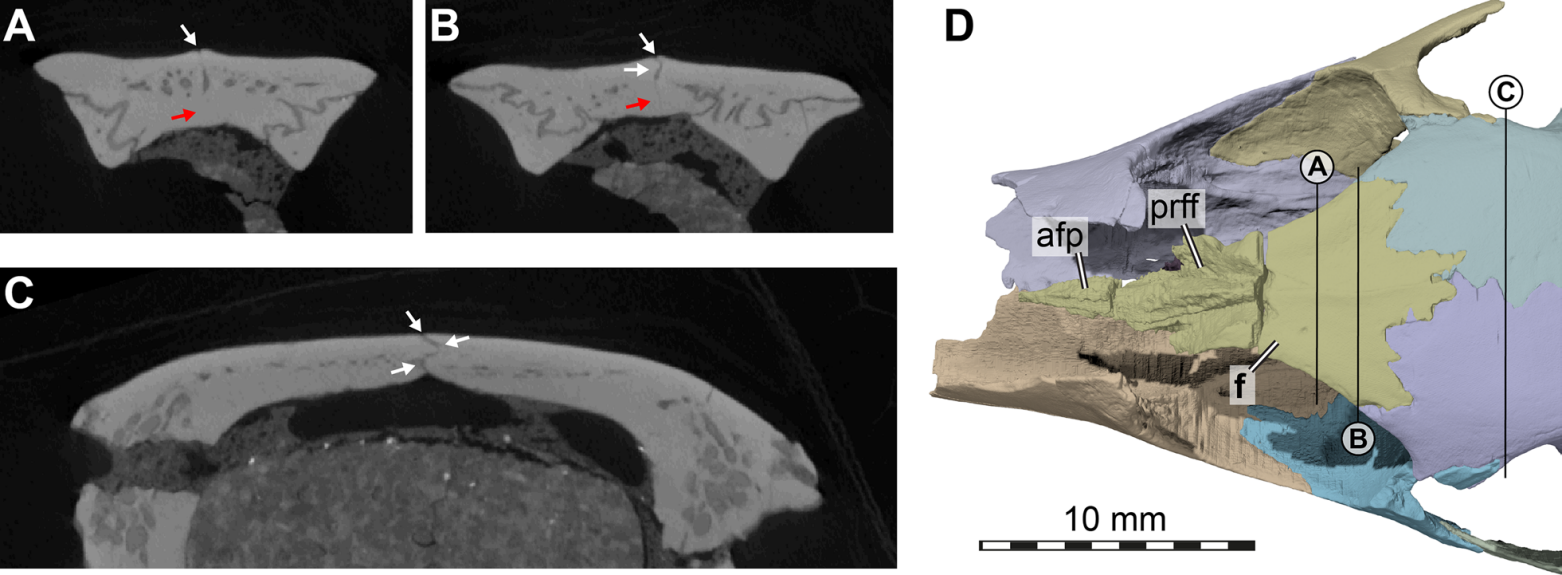
Computed tomographs through the cranium of *Plastomenus thomasii* (AMNH FR 6015) and frontal morphology. **A**–**C**, cropped µCT slices through the frontal (**A**–**B**) and parietal (**C**) region of the skull roof. **D** Partial cranium in dorsal view, with prefrontals removed to illustrate the anterior frontal process. Note that bones are labelled in bold. White arrows show examples of traceable suture lines, red arrow indicates region in which an interfrontal suture would have been expected if the bones were not fused. Straight lines in D indicate slice positions for (**A**–**C**). *apf* anterior frontal process, *f* fused frontals, *prff* prefrontal facet on frontal

**Fig. 7:**
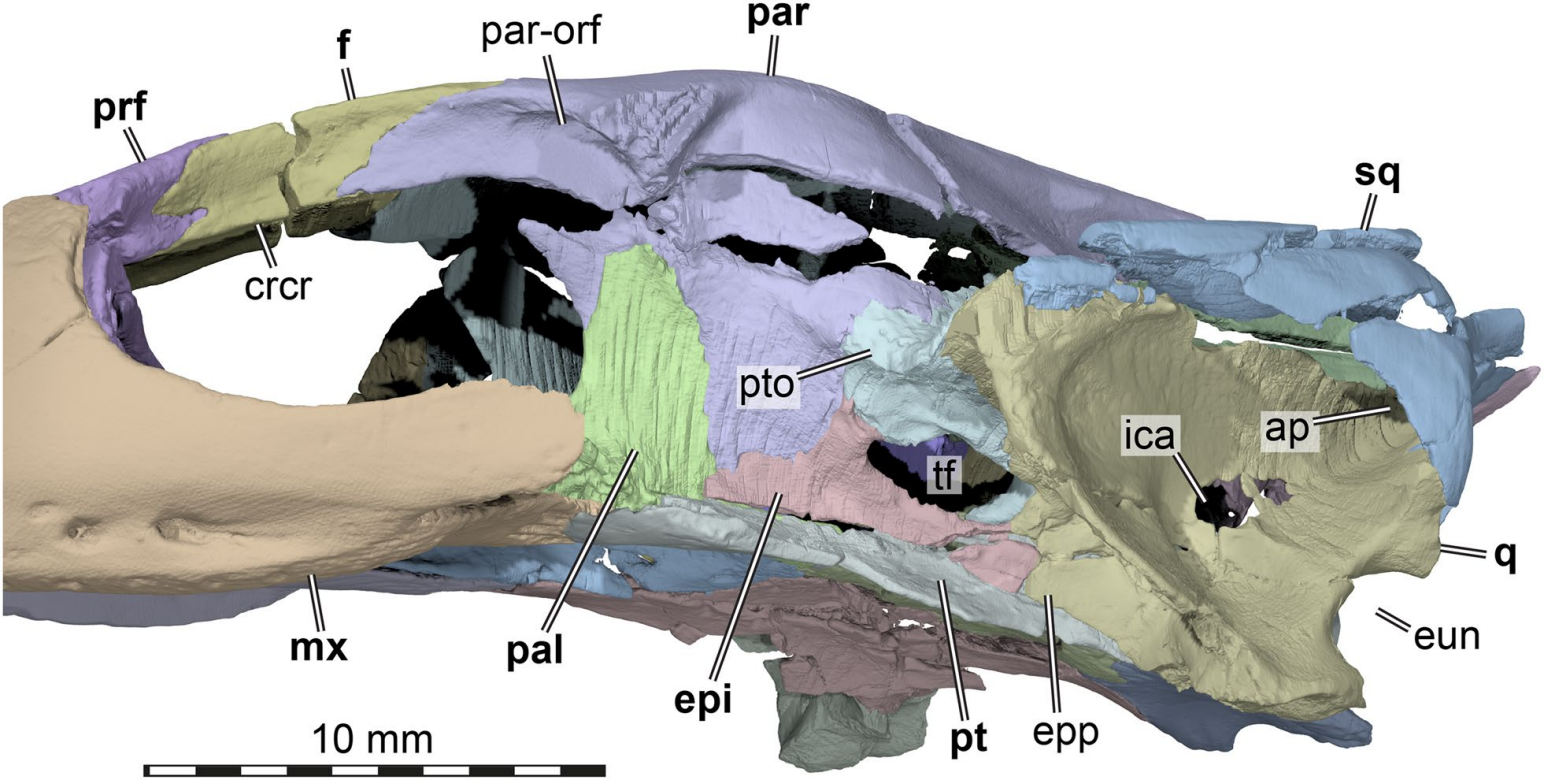
Three dimensional renderings of the exterior trigeminal region of the cranium of *Plastomenus thomasii* (AMNH FR 6015). Partial cranium in left anterolateral and slight ventral view, with jugal and quadratojugal removed. Note that bones are labelled in bold. *ap* antrum postoticum, *crcr* crista cranii, *epi* epipterygoid, *epp* epipterygoid process of quadrate, *eun* Eustachian notch, *f* frontals (fused), *ica* incisura columellae auris, *mx* maxilla, *pal* palatine, *par-orf* orbital fossa of the parietal, *par* parietal, *prf *prefrontal, *pt* pterygoid, *pto* processus trochlearius oticum, *q* quadrate, *sq* squamosal, *tf* trigeminal foramen

**Fig. 8:**
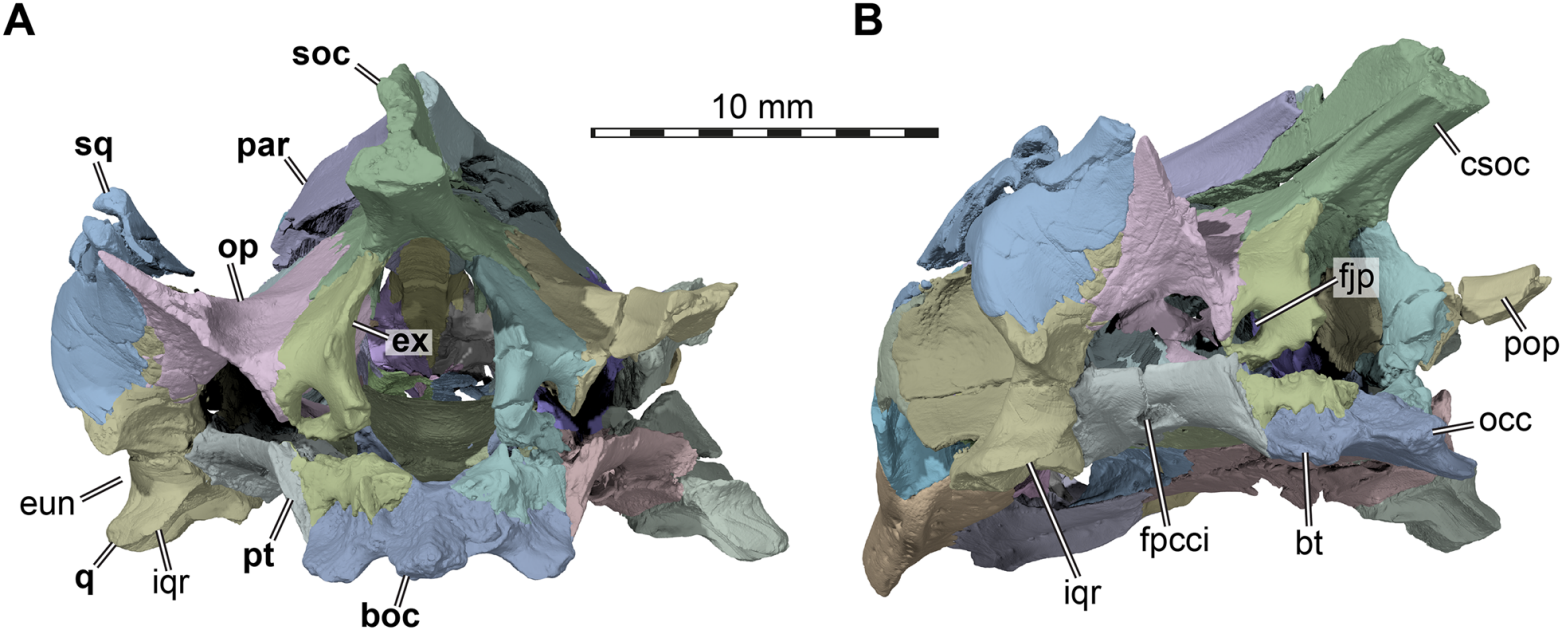
Three dimensional renderings of the posterior of the cranium of *Plastomenus thomasii* (AMNH FR 6015). **A** posterior view; **B** left posterolateral and slight ventral view. Note that bones are labelled in bold. *boc* basioccipital, *bt* basi tuber, *csoc* crista supraoccipitalis, *eun* Eustachian notch, *ex* exoccipital, *fjp* foramen jugulare posterius, *fpcci* foramen posterius canalis carotici interni, *iqr* infolding quadrate ridge, *occ* occipital condyle, *op* opisthotic, *par* parietal, *pop* paroccipital process, *pt* pterygoid, *q* quadrate, *soc* ssupraoccipital, *sq* squamosal

**Fig. 9:**
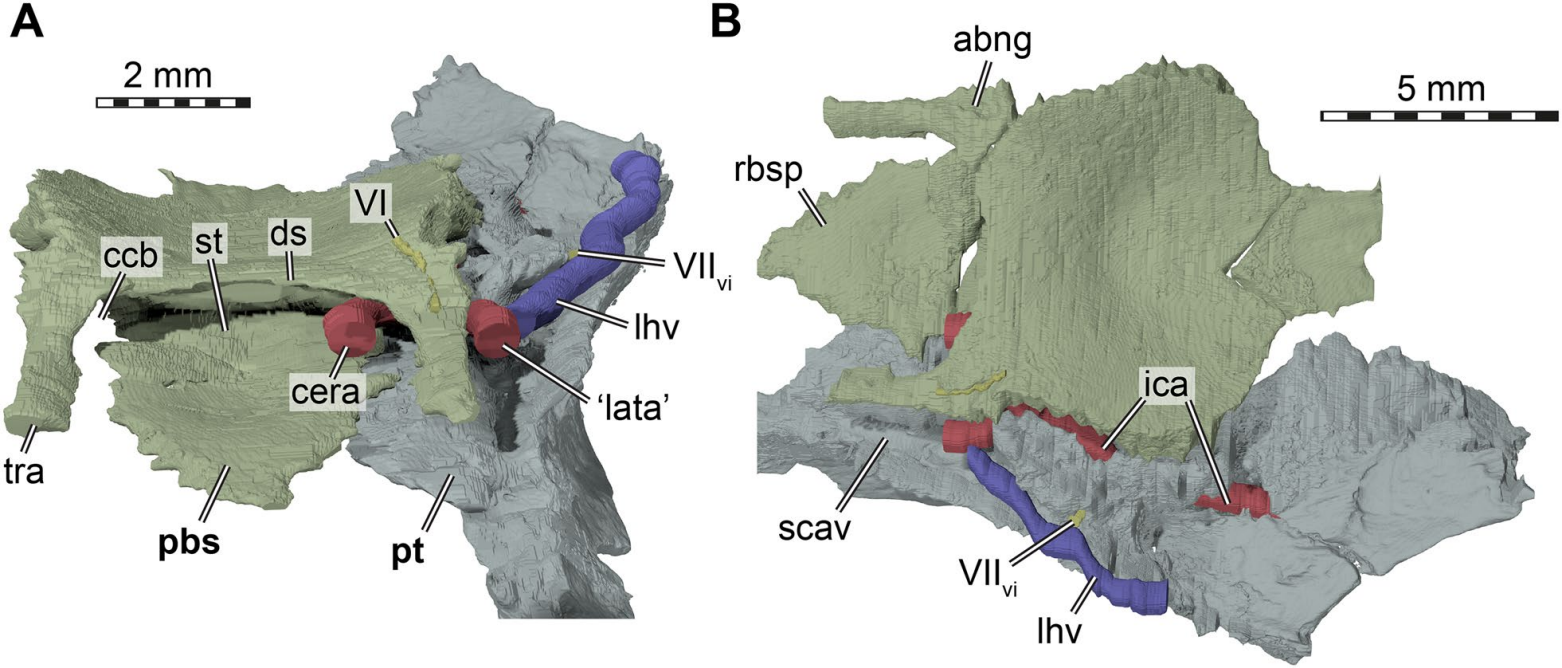
Three dimensional renderings of the partial basicranium of *Plastomenus thomasii* (AMNH FR 6015) with reconstructed arteries and nerves. **A** anterodorsal view onto parabasisphenoid and left pterygoid; **B** dorsal view. Note that bones are labelled in bold. Nerves are reconstructed in yellow, arteries in red, and veins in blue. *abng* abducens nerve groove, *ccb* canals caroticus basisphenoidalis, *cera* cerebral artery, *ds* dorsum sellae, *ica* internal carotid artery, ‘*lata*’ lateral artery (possible palatine artery or pseudopalatine artery or mandibular artery), *lhv* lateral head vein, *pbs* parabasisphenoid, *pt*, pterygoid, *rbsp* rostrum basisphenoidale, *scav* sulcus cavernosus, *st* sella turcica, *tra* trabecula, *VI* abducens nerve, *VII*_*vi*_ vidian branch of facial nerve

**Fig. 10:**
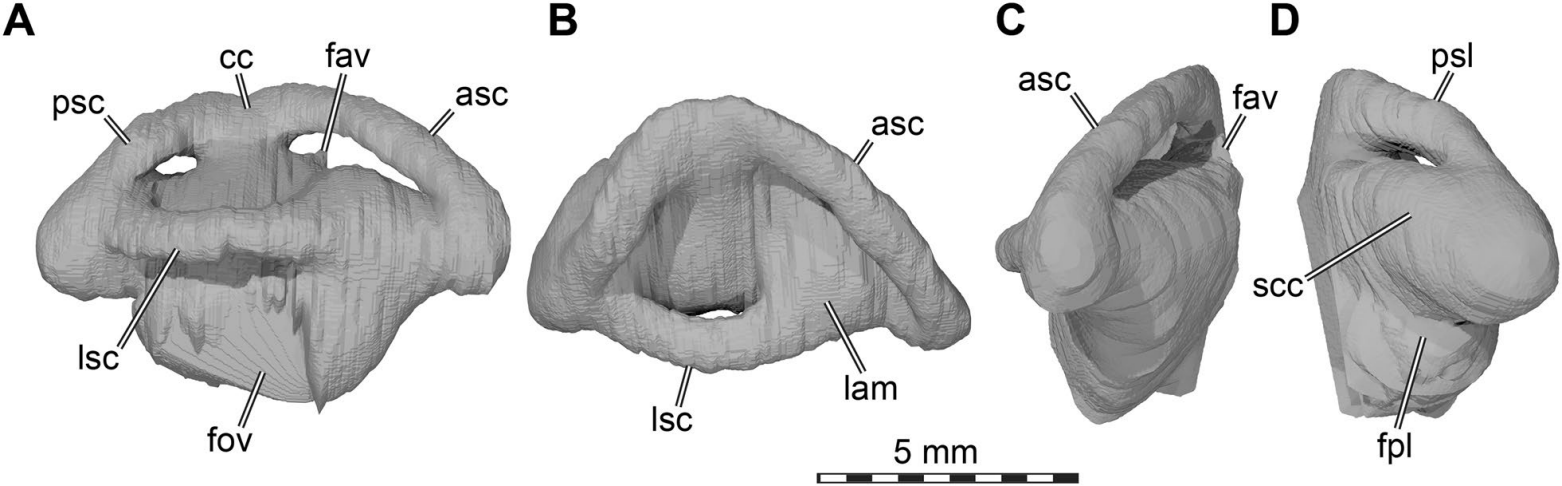
Three dimensional renderings of the right endosseous labyrinth of *Plastomenus thomasii* (AMNH FR 6015). **A** lateral view; **B** dorsal view; **C** anterior view; **D** posterior view. Note that the fenestra ovalis region is incomplete due to breakage along the prootic and opisthotic. Also, the endocast of the fenestra perilymphatica is incomplete due to breakage. *asc* anterior semicircular canal, *cc* common crus, *fav* foramen aquaducti vestibuli, *fov* fenestra ovalis, *fpl* fenestra perilymphatica, *lam* lateral ampulla, *lsc* lateral semicircular canal, *psc* posterior semicircular canal, *scc* secondary common crus

**Fig. 11:**
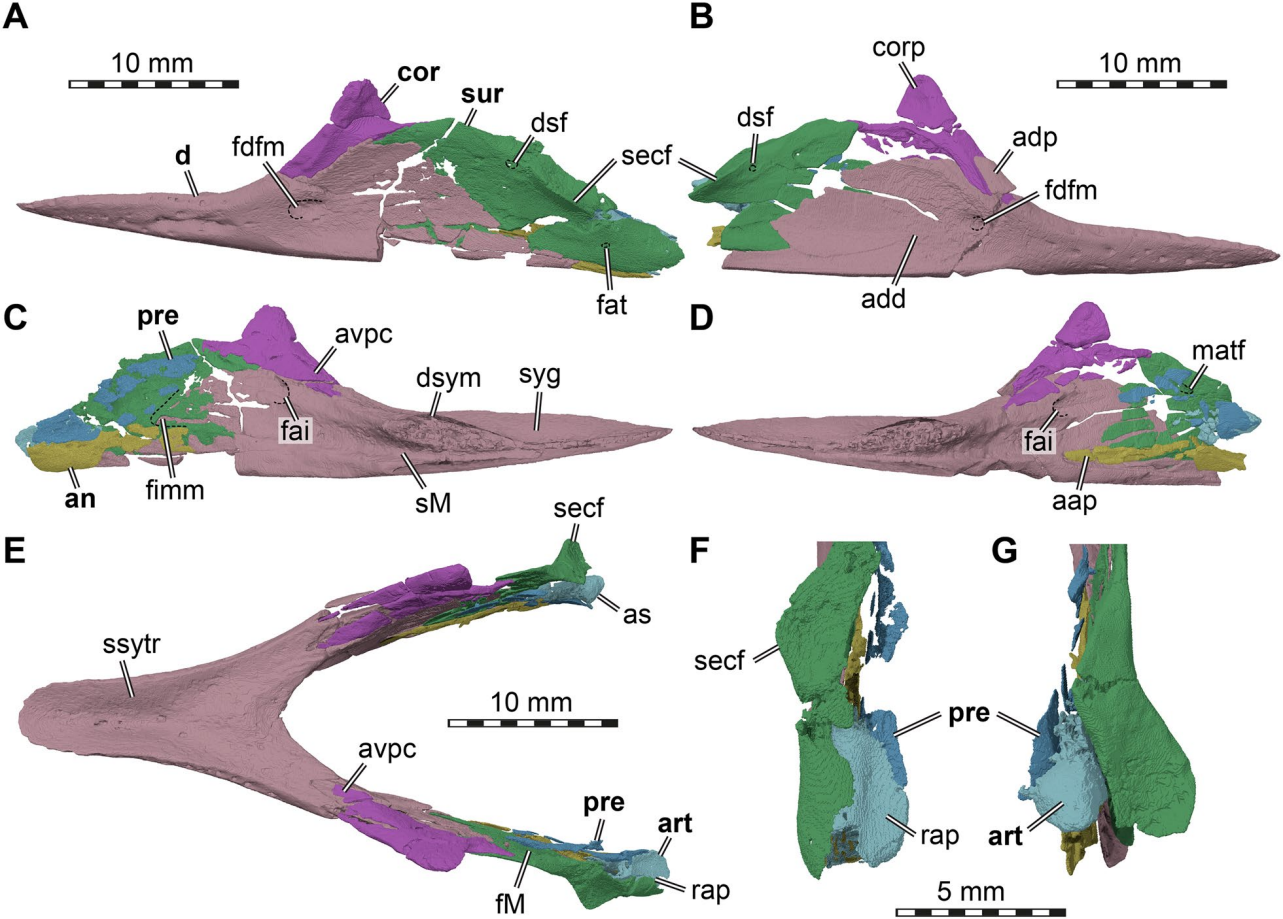
Three dimensional renderings of the mandible of *Plastomenus thomasii* (AMNH FR 6015). **A** left lateral view; **B** right lateral view; **C** medial view of left mandibular ramus; **D** medial view of right mandibular ramus; **E** dorsal view; **F** posterodorsal view of posterior end of left mandibular ramus; **G** posterodorsal view of posterior end of right mandibular ramus. Medial views were achieved by artificially cutting the dentary along the symphysis. Note that bones are labelled in bold. Dashed lines circumscribe foramina to highlight their positions. *aap* anterior angular process, *add* adductor fossa, *adp* ascending dentary process, *art* articular, *as* articulation surface, *cor* coronoid, *corp* coronoid process, *d* dentary, *dsf* dorsal surangular foramen, *dsym* dorsal symphyseal mount, *fai* foramen alveolare inferius, *fat* foramen auriculotemporalis, *fdfm* foramen dentofaciale majus, *fimm* foramen intermandibulare medius, *fM* fossa Meckelii, *matf* medial auriculotemporal foramen, *pre* prearticular, *rap* retroarticular process, *secf* surangular ectocondylar flange, *sM* sulcus Meckelii, *ssytr* spatulate symphyseal triturating surface, *syg* symphyseal groove

AMNH FR 6015 comprises a nearly complete cranium and mandible (Figs. 1–11). The anterior tip of the snout of the cranium is broken and missing, while the posterolateral portion of the left mandible is broken off but is present. The skull is 24 mm wide (measured between the lateral margins of the articular processes of the quadrate) and 12 mm high (measured as a straight line through the specimen from the skull roof to the floor of the basicranium). The preserved midline length between the occipital condyle and the anteriormost point of the intermaxillary contact is 43 mm. The left maxilla extends for another 3 mm from this point with its partially preserved anterior process. Thus, the skull would have roughly measured 50 mm in total length if the premaxillae were preserved. For more measurements, we refer to the digital models that we deposited, from which measurements can be extracted with digital tools.

The skull is elongate and tubular in shape. The interorbital bar is relatively narrow and orbits oriented dorsolaterally. The upper temporal emargination is extremely deep, reaching just behind the orbit to form narrow postorbital bars. The lower temporal emargination, on the other hand, only reaches the lower level of the orbit.

## Prefrontal

Both prefrontals are preserved and near complete, except for the anteriormost ends which are broken off. The prefrontal is a large element in AMNH FR 6015, as is typical for trionychids, and extends from the anterior third of the orbit anteriorly to form the roof of the elongated snout and narial region (Figs. 3A, C, D; 5). Although the anterior margin of both prefrontals is damaged, the nearly intact left element suggests that each element possessed a deep concavity framed by a short and narrow lateral process and wider and more elongate medial process. The dorsal margin of the apertura narium externa is therefore deeply emarginated laterally (Fig. 4A). Each prefrontal has a long midline contact with its counterpart, and a long ventrolateral contact with the ascending process of the maxilla (Figs. 3A, C–D, 5C). Posteriorly, the prefrontal narrows towards the midline and develops a pointed process. Overall, the prefrontal covers the anterior two thirds of the frontals (Fig. 3A, C), which have a long anterior process. Posteroventrally, the prefrontal forms a long ventral process which bifurcates into a ventrally extending lateral ramus and a medioventrally extending medial ramus (Fig. 5C). These rami are subequal in length and are separated by a U-shaped notch that forms the anterodorsal margin of the foramen orbito-nasale. Unlike in non-trionychid cryptodires, the foramen orbito-nasale is ventrally open to the pronounced narial passage, which is particularly strongly developed in AMNH FR 6015 (Fig. 5C). The medial ramus of the ventral process contacts the vomer and laterally frames the rounded fissura ethmoidalis. The more robust lateral process lies against the maxilla within the orbital cavity (Fig. 5C, D).

## Frontals

The frontals are preserved and complete, although a mediolaterally extending fracture separates the anterior part from the posterior part. The frontals of AMNH FR 6015 are completely fused: µCT slices show that the two bones form a single unit (Fig. 6A, B), with only a slight indication of what could be a nearly closed suture line in some of the posteriormost slices through the complex (Fig. 6B). This is notwithstanding a fine median line on the dorsal surface of the bone (Fig. 6), which appears to be a suture, but does not continue internally through the bone. Considering that all other sutures of the skull are easily distinguishable in the slice data (Fig. 6A–C), we treat the frontals as “fully fused” even though the median line on the dorsal surface and posterior suture trace may suggest fusion during ontogeny and reminds of the condition of other trionychids, in which these bones are unfused.

The frontals are relatively small in terms of their externally visible exposure in dorsal view, but much lies hidden from view below the prefrontals (Fig. 3A). They are restricted to the interorbital area, where they contact and broadly underly the prefrontals anteriorly and contact and partially overly the parietals posteriorly. When the prefrontals are digitally removed, the true extent of the frontals becomes apparent, which is much larger: there is a long anterior process that underlies the prefrontals, which exceeds the externally visible length of the frontals by more than 100% (Fig. 6D). At the anterior end, the anterior process is as mediolaterally broad as the externally visible interorbital bar. Along the midline, there is a raised crest on the dorsal surface of the anterior process, which extends into the interprefrontal suture ventrally (Fig. 6D). To either side of the crest, a laterally extending expansion is present with a deep triangular recess along its dorsal surface for articulation with the posterior process of the prefrontal. At the level of the fissura ethmoidalis, which marks the transition from orbital to nasal cavity, the lateral expansions of the anterior process that buttress the prefrontal end, and the central portion of the anterior process tapers as an anteriorly thinning spur underneath the prefrontals.

The lateral margin of the frontals forms the dorsal margin of the orbit (Fig. 3A, C–D). Posteriorly, the frontals broaden mediolaterally, forming posterolateral processes that overlap the dorsal surface of the parietal. Along the posterior suture with the parietals, the frontals form a posterior process that extends between the parietals on the skull roof (Fig. 3A). However, this process only overlaps the parietals, which buttress the process from below.

The ventral surface of the frontals bears the sulcus olfactorius, which is notably shallow due to the low crista cranii (Fig. 5D).

## Parietal

The two parietals of AMNH FR 6015 are preserved and near complete, though heavily fractured in several areas. Their overall morphology is still observable (Figs. 3A, C–D, 5D, 7). The parietal forms the bulk of the dorsal skull roof (Fig. 3A), a large part of the secondary lateral wall of the braincase (Fig. 7), and contributes to the formation of the processus trochlearis oticum (Fig. 7). It contacts the frontal anteriorly, the jugal along a short lateral process, the palatine and epipterygoid along the descending process, and it overlaps the prootic and supraoccipital along its ventrolateral margins. The parietal of AMNH FR 6015 is very clearly excluded from contributing to the trigeminal foramen (Fig. 7).

The anterior margin of the parietal underlaps the fused frontals. From here, the descending process emerges as a posterior continuation of the low crista cranii of the frontals (Fig. 7). The descending process quickly becomes ventrally deeper in the posterior orbital area, where it lies against the medial surface of the ascending process of the palatine (Fig. 7). Anterolaterally, the margin of the parietal broadly contributes to the orbit, a highly unusual feature among turtles (Fig. 3A, C, D). The parietal is mediolaterally broadest in this area, and contacts the jugal along a relatively short lateral process (Figs. 3C, D, 7). Ventrally, this process is buttressed against the descending process of the parietal by a vertical, mediolaterally extending lamina, which forms an arched roof for the passage between the temporal and orbital cavities. As this morphology between the temporal and orbital fossae is reminiscent of the paracryptodiran and pleurodiran condition (Evers et al., 2020, 2021; Gaffney, 1979), we call this the sulcus palatino-pterygoideus, a term that is historically associated with pleurodiran anatomy only (e.g., Gaffney et al., 2006). The ventral lamina, lateral process, anterior process, and orbital margin of the parietal define a recess anterolaterally, forming a deep posteromedial pocket of the orbital fossa (Fig. 7).

The dorsal surface of the parietal is smooth and without texture. The interparietal suture is broadly interdigitated anteriorly, but relatively straight posteriorly (Fig. 3A). The parietals converge posteriorly to a thinning processes, which cap the anterior region of the supraoccipital crest. Toward the upper temporal fossa, the skull roof part of the parietals is clearly demarked by a step-like margin that forms the transition to the sheeted ventrally descending process of the parietal. Along the otic capsule and temporal fossa, this sheet of bone is laterally bulged and extends onto the prootic (Fig. 3A). The braincase in this part housed the cerebellar region of the brain, which must have been notably laterally expanded (Fig. 5A, C, D), as in many trionychids (Werneburg et al., 2021a). At the anterior end of the otic capsule, the parietal expands laterally to form the most medial portions of the processus trochlearis oticum (Fig. 7), which is otherwise largely formed by the prootic (see below). Anterior to this process, the descending process becomes strongly ventromedially inclined. Despite its deep ventral extent, it neither reaches the pterygoid on the external nor on the internal surface, but is separated from this bone by the epipterygoid posteriorly and the palatine anteriorly (Fig. 7).

The ventral surface of the cavum cranii bears no salient features, except for a median, tongue-like depression immediately anterior to the supraoccipital (‘carrf’ in Fig. 5D). In a braincase endocast, this depression would form the “cartilaginous rider”, which indicates that parts of the cartilaginous planum supraseptale of the chondrocranium remained unossified at the point of death of AMNH FR 6015 (Werneburg et al., 2021b).

## Postorbital

*Plastomenus thomasii* seems to have completely lost the postorbital bone, based on the well-preserved right side of the fossil (Fig. 3D). This contrasts with the reconstructive line drawings provided by Gaffney (1979: Fig. 183). The postorbital in trionychids usually is a small, triangular bone in the posterior orbital margin, and wedged between the frontal anteromedially, the jugal ventrolaterally, and the parietal posteromedially (e.g., *Apalone spinifera*; but also see Gaffney, 1979; Meylan, 1987; Vitek & Joyce, 2015). The jugal and parietal usually form a contact posterior to the postorbital (e.g., Gaffney, 1979; Meylan, 1987). In AMNH FR 6015, the typical jugal-parietal arrangement can be observed, with a posterodorsally pointed ascending process of the jugal inserted into a respective facet on the anterolateral process of the parietal that roofs the passage between temporal and orbital cavities (Fig. 3D). Although there is a break separating the dorsal skull roof plate and the ventrally descending process of the parietal (which, evidently, was mistaken by Gaffney (1979) as the postorbital-jugal contact), both pieces fit together nicely, and, if articulated, leave no room or indication for the presence of a postorbital (see Fig. 3D). We also meticulously screened the µCT slices through the dorsal jugal process to identify the presence of a small postorbital along the process, but we could not find any positive evidence for this bone.

## Jugal

Both jugals are preserved in AMNH FR 6015. The right element is basically complete except for the distal end of the posteriorly extending ventral process (Figs. 3D, 5A, B). The left jugal is missing a large portion of the dorsal process as well as the distal end of the posteriorly extending ventral ramus (Figs. 3C; 4B). The jugal forms a triradiate bar in lateral view. It forms the posterior margin or the orbit and the anterior margin of the deep temporal emargination with its dorsal process and the shallow cheek emargination along the ventral margin of its posterior process (Fig. 3C, D). Posteriorly, the jugal has a vertical sheet like process that shortly overlaps the quadratojugal laterally (Fig. 3A, C). Articular facets on the left quadratojugal suggest that the posterior process is damaged on both sides of the skull. Indeed, the facets on the quadratojugal suggest that the jugal was forked posteriorly and that the dorsal branch contacted the squamosal along the upper temporal emargination (Fig. 4B), as in some individuals of *Gilmoremys lancensis* (Joyce & Lyson, 2011). The ventral margin of the jugal is gently concave and raised from the level of the triturating surface, thereby forming a modest cheek emargination that continues posteriorly along the ventral margin of the quadratojugal (only preserved on the left side; Fig. 3C). The dorsal process of the jugal is anteroposteriorly short between the orbit and temporal emargination and contacts the lateral surface of the lateral process of the parietal along its entire dorsoventral depth (contra Gaffney, 1979 and Vitek & Joyce, 2015, who list the absence of a jugal-parietal contact). This contact with the parietal is the only one present along the dorsal process of the jugal as the postorbital seems to be absent (see; Fig. 3C, D). The anterior process of the jugal appears anteroposteriorly short in lateral view, as it is laterally overlapped by the posterior maxillary process. However, within the orbital cavity, the process extends across most of the orbital fossa floor (Fig. 5B) and nearly reaches the prefrontal in an anteriorly pointed end (Fig. 3A). On the medial surface of the jugal, there is a well-developed septum orbitotemporale, which forms a vertical lamina that borders the orbit posteriorly (Fig. 5B). The septum constricts the passage of the sulcus palatino-pterygoideus to a relatively narrow opening. At the ventral base of the septum, the medial process of the jugal contacts the maxilla, the palatine, and, with a small posteriorly directed ramus, the pterygoid along the strongly reduced external pterygoid process (Fig. 3B).

## Quadratojugal

The quadratojugal is fully preserved on the left side of AMNH FR 6015, but only a small fragment of this bone is present on the right side of the fossil. The quadratojugal is a small triradiate bone in the temporal region of the skull (Fig. 3C). The anterior process contacts the medial surface of the jugal, while the posterior part of the bone is forked into a posterodorsal and a posteroventral process. Both of these are delicate and taper to a point. The two posterior processes are separated by a U-shaped notch and contact the quadrate along its anterodorsal, anterior, and anteroventral margins. The ventral margin of the quadratojugal along the anterior and posterior processes defines a relatively shallow cheek emargination (see also jugal; Fig. 3C). The posterodorsal process extends along the anterodorsal margin of the quadrate and contacts the squamosal, which is accommodated by a dorsally facing facet. The extensive articular facets on the lateral side of the quadratojugal suggest that the quadratojugal only had a small contribution to the external surface of the skull and did not contribute to the upper temporal emargination (Fig. 4B), as in some individuals of *Gilmoremys lancensis* (Joyce & Lyson, 2011).

## Squamosal

Only the left squamosal is preserved in AMNH FR 6015, although badly fragmented (Figs. 3A, C, 5B, 7, 8). The squamosal likely contacted four other bones: its most extensive contact is with the quadrate, which it caps posterodorsally, but an anterior process also contacts the quadratojugal and likely also the jugal, and an opisthotic contact is posteriorly present along a posterior horn-shaped process. The squamosal forms the dorsal and posterodorsal margin of the cavum tympani (Figs. 3C, 7) and much of the voluminous antrum postoticum (Fig. 7). The antrum is morphologically simple and continuous with the cavum tympani, rather than being a clearly delimited cavity distinct from the cavum. The posterior process of the squamosal is incomplete, but it formed a horn-like process together with the tip of the paroccipital process of the left opisthotic (Figs. 3A, 4C, 8).

## Premaxilla

Premaxillae are not preserved in AMNH FR 6015.

## Maxilla

Both maxillae of AMNH FR 6015 are preserved but broken along their anterior ends. Despite this, all major features of the maxillary morphology can be observed in the fossil (Figs. 3, 5, 7). The maxilla contacts the prefrontal, the vomer, the palatine, the pterygoid, and the jugal, as well as presumably the premaxilla, which is not preserved. A maxilla-frontal contact was included in the illustration of Gaffney (1979) and listed as a diagnostic characteristic for *Plastomenus thomasii* by Vitek and Joyce (2015), but is not actually present.

The maxilla is elongated anteriorly to form a telescoped snout. The ascending process of the maxilla overlies the prefrontal, which forms a deep triangular facet for this articulation. The ascending process and the posterolateral parts of the maxilla contribute to the anteroventral and ventral orbit margins (Fig. 3C, D).

The posterior end of the maxilla bifurcates into a lateral process and a medial process. The lateral process contacts the jugal in the cheek region (Fig. 3B). Along its dorsal surface, the medial process inserts between and contacts the palatine and jugal at the base of the fenestra interorbitale (Fig. 5A). On the ventral surface of the skull, the medial process forms the posterior end of the triturating surface, extends along the posteromedial process of the jugal to contact the pterygoid, and frames the anterolateral margin of the internal naris (Fig. 3B). The snout forms an extensive secondary palate. However, the right and left triturating surfaces remain separated for most of the length of the maxilla, as a narrow midline gap is present between them (Fig. 3B). In the posterior section of the triturating surface, this midline gap is dorsally bridged by the vomer, which is not truly inserted between the maxillae, but sits dorsally on top of their medial margins (Fig. 5C). At the level of the anterior end of the vomer, the midline gap between the maxillae becomes somewhat mediolaterally broader, and the intermaxillary region becomes gently vaulted dorsally (Figs. 3B, 5D), without, however, developing a tongue groove, which is developed in most trionychids, including *Axestemys infernalis* (DMNH 130951), *Pelodiscus sinensis*, *Amyda cartilaginea*, *Cyclanorbis senegalensis*, *Lissemys punctata*, or *Apalone spinifera*. Anterior to the vaulted region, the right and left maxillae form an interdigitated contact zone with one another posterior to the position of the incompletely preserved foramen intermaxillare (Figs. 3B, 4C).

The triturating surface of the maxilla is laterally framed by a robust labial ridge (Figs. 3B–D, 5D, 7). The periphery of this ridge is pierced by several relatively large neurovascular foramina both medially and laterally, which connect to a central supraalveolar canal that traverses the maxilla anteroposteriorly (see below). Accessory ridges of the maxillary triturating surface are absent (Vitek & Joyce, 2015), which is different from *Gilmoremys lancensis* (Joyce et al., 2011). The lateral surface of the maxilla is recessed along the labial ridge to a fossa that indicates the former position of a keratinous rhamphotheca.

The dorsal surface of the maxilla is dominated by two transversely concave regions separated by an anteroposteriorly extending dorsal crest, which starts at the base of the prefrontal-maxilla contact and extends posteriorly toward the anterior process of the palatine. For the purpose of this description, we term this feature the “suborbital crest” (Figs. 3A, 5B, C). The surface lateral of the suborbital crest is the floor of the orbital fossa, while the nasal passage is medial to it (Fig. 5B, C). This arrangement is similar to cyclanorbines (e.g. *Cyclanorbis senegalensis*, *Lissemys punctata*), which also have a clearly defined suborbital crest, but differs from the condition in trionychines, in which a clearly defined border between orbital fossa and narial passage is absent (*Apalone spinifera*, *Chitra chitra*, *Pelodiscus sinensis*) or only very weakly raised (*Amyda cartilaginea*). The narial passage of AMNH FR 6015 is a transversely concave, anteroposterior trough formed by the maxilla, palatine, and vomer between the internal naris and the nasal cavity (Fig. 5B). The passage is mediolaterally broad, which also leads to a large diameter of the foramen orbito-nasale (Fig. 5C), which is framed dorsally by the prefrontal. Due to the presence of an elongated narial passage, the internal naris is posteriorly removed from the nasal cavity (Figs. 3B, 5B), justifying the identification of an extensive secondary palate.

Within the orbital fossa, immediately lateral to the anterior end of the suborbital crest, there is a large supramaxillary foramen (= foramen supramaxillare of Gaffney, 1972) in AMNH FR 6015 (Figs. 3A, 5B, C). The foramen opens into a large, anteroposteriorly oriented infraorbital canal (= canalis infraorbitalis of Gaffney, 1972) or supraalveolar canal (= canalis alveolaris superior of Gaffney, 1972) that traverses most of the maxilla, and which houses the openings of numerous small and large ventrolateral and ventromedial canals that exit to either side of the labial ridge on the outer surface of the maxilla. There is no morphological justification to distinguish between an infraorbital canal and a supraalveolar canal in AMNH FR 6015, as there is only a single anteroposterior canal within the maxilla. As this canal largely parallels and supplies the labial ridge and is topologically anterior to the orbit, we use “supraalveolar canal” herein. A distinction between both canals was made by Albrecht (1967) for *Apalone spinifera* (= *Trionyx spinifer *sensu Albrecht, 1967), as this taxon, as with cryptodires in general, has two separate entry foramina into the supraalveolar canal: a posterior supramaxillary foramen, and a supraalveolar foramen (= foramen alveolare superius of Albrecht, 1967). Both of these foramina carry arteries that supply the labial ridge region of the maxilla (Albrecht, 1967). In AMNH FR 6015, the supramaxillary foramen seems to be the only supply for the interior of the maxilla. This is again similar to cyclanorbines, which lack a supraalveolar foramen (pers. obs. on above mentioned specimens). The supraalveolar foramen of trionychines is positioned within the nasal cavity, a short distance anterior to the foramen orbito-nasale (Albrecht, 1967; pers. obs. on above mentioned specimens). Often, the bone posterior to the foramen is slightly grooved, indicating the course of the supraalveolar artery toward the foramen (e.g., *Apalone spinifera*: FMNH 22178). AMNH FR 6015 also has an opening to the infraorbital canal within the nasal fossa (Fig. 5A). However, the opening forms a dorsal fenestra into the supraalveolar canal, exposing a short section of the latter. It seems likely that the opening serves as an exit for an arterial branch to supply the internal nasal tissue, but the opening is neither posteriorly directed, nor associated with a posterior groove. As such, although topologically approximately in the position of a supraalveolar foramen, we here argue that the supraalveolar fenestra of AMNH FR 6015 is not homologous to the supraalveolar foramen of trionychines. Tentative evidence for this also comes from the presence of the suborbital crest. This crest is absent in trionychines, and thus the supraalveolar artery can cross from the orbital region into the narial passage, from where it enters the supraalveolar foramen. In cyclanorbines and AMNH FR 6015, the suborbital crest prohibits this branch to cross medially into the narial passage, leaving the supramaxillary foramen as the singular opening into the maxilla.

## Vomer

The vomer is completely preserved in AMNH FR 6015 as a vertically oriented and anteroposteriorly elongate bone (Figs. 3B, 5). It contacts the prefrontal, maxilla, and palatine. Ventrally, the vomer is bifurcated into ventrolaterally directed flanges, which bridge a narrow midline gap between the maxillae. The medial margin of the maxilla is gently upcurved along the vomer contact (Fig. 5D), so that the vomer is essentially excluded from the ventral side of the skull and specifically the triturating surface (Fig. 3B). Near the posterior end of the contact with the maxilla, the vomer has a small foramen on either side, which connects its dorsal surface with the palatal surface. Similar foramina are absent in extant trionychids in which the content of the foramina could be observed directly (e.g., *Amyda cartilaginea*; *Apalone spinifera*; *Chitra chitra*; *Cycloderma frenatum*; *Lissemys punctata*; *Pelodiscus sinensis*). Lyson and Joyce (2011) identified foramina in the same position of *Gilmoremys lancensis* and referred to these as foramina for the pseudopalatine artery, but we raise the possibility that these are related to the anterior nasal artery and therefore homologous to the prepalatine foramina (see “Discussion”). We preliminarily call these “vomer foramina” (Fig. 5C). Posterior to the maxillae contact, the vomer of AMNH FR 6015 becomes a median sheeted process that divides the right and left narial passages (Figs. 3A, 5B) and extends ventrally to the interpalatine contact area. At the anterior end, the vomer forms short dorsolateral processes, that diverge from the midline to form the sulcus vomeri at the floor of the fissure ethmoidalis (Fig. 5). The dorsolateral process contacts the prefrontal and thus contributes to the foramen orbito-nasale.

## Palatine

Both palatines are preserved and near complete in AMNH FR 6015 (Figs. 3B, 5A, B, 7). Although both elements are partially broken along their more fragile areas, creating holes along their central parts, the complete morphology of these bones can be appreciated. The palatine principally consists of a domed plate that roofs the narial canal (Fig. 3B), and a sheet-like vertical process that ascends from the dorsal surface of the horizontal plate (Fig. 7). The palatine contacts the parabasisphenoid posteriorly, the pterygoid laterally, the maxilla and jugal anterolaterally, the vomer anteromedially, the parietal dorsally, the epipterygoid posterodorsally, and its counterpart medially.

Ventrally, the right and left palatines contact one another in a long, weakly interdigitated suture (Fig. 3B). This contact persists in the anterior third of the bone, in which the palatines overlap the posterior end of the vomer (Fig. 5A, B). Each palatine is almost tube-like in cross section, with the main body curving ventromedially as it extends laterally, forming a strong anteroposterior depression on the ventral surface between the slightly protruding median interpalatine suture and the lateral margin of the bone. This depression is the posterior beginning of the nasal passage and roofs the internal naris. The palatine is strongly vaulted around this passage (Fig. 5A, D). The lateral margin of the palatine contacts the pterygoid posteriorly, and the maxilla anteriorly. On the ventral surface near the triple junction of palatine, maxilla and pterygoid, there are two small foramina palatinum posterius, which are anteroposteriorly aligned (e.g., Vitek & Joyce, 2015, but contra the reconstruction of Gaffney, 1979). The posterior foramen is completely enclosed within the lateral palatine margin (Fig. 3B), whereas the slightly smaller anterior foramen is situated in the maxilla-palatine suture on the right skull side, but entirely within the palatine on the left side (Fig. 3B). The foramina palatinum posterius internally merge with the anteroposteriorly directed intrapalatine canal (= canalis intrapalatinus of Albrecht, 1967; see below).

Anterolaterally, the palatine has a triangular process that laps onto the maxilla between the orbital cavity and the nasal passage. The dorsal surface of this process bears an anteroposterior ridge (Fig. 5B), which continues anteriorly onto the maxilla as the suborbital crest (see above).

Laterally, the palatine expands to contact the jugal at the base of the septum orbitotemporale (Fig. 5A). At the level of the septum orbitotemporale, the palatine develops a dorsally tall ascending process (Figs. 5A, B, 7). The process is robust at its base and forms the foot of the anterior margin of the braincase. The majority of the medial surface area of the dorsal palatine process lies against the lateral surface of the descending process of the parietal, so that both together form the majority of the secondary wall of the braincase (with parts of it formed by the epipterygoid posteroventrally; Fig. 7).

Immediately lateral to the base of the dorsal process and anteroventral to the anterior margin of the dorsal process, there is a large foramen in the dorsal surface of the palatine (Fig. 5A, B), which posteriorly leads into the intrapalatine canal (sensu Albrecht, 1967). The intrapalatine canal of AMNH FR 6015 has two posterior openings: one is the foramen palatinum posterius, which opens ventrally on the skull surface and carries the inframaxillary artery from the skull inside to the mouth (Albrecht, 1967). The second opening is posteromedially into the sulcus cavernosus (Fig. 5B). This part of the intrapalatine canal system is much smaller in terms of canal diameter, and it seems likely that it carried at least the vidian nerve (Albrecht, 1967). The anterior foramen of the intrapalatine canal seems to be aligned with the supramaxillary foramen within the maxilla, as in *Gilmoremys lancensis* (Joyce & Lyson, 2011). This idea is reinforced by both foramina being immediately lateral to the suborbital crest, and by the fact that the dorsal surface of the maxilla forms a subtle trough posterior to the supramaxillary foramen, and toward the intrapalatine canal (Fig. 5B). This indicates that the supramaxillary and supraalveolar arteries may have extended through the ‘vidian’ part of the intrapalatine canal.

## Quadrate

Both quadrates are preserved in AMNH FR 6015. The left element is near complete despite some cracks and minor damage within the cavum tympani (Fig. 3C), whereas the right element is missing some of its posterior parts along the cavum tympani and articular process (Fig. 3D). The quadrate contacts the quadratojugal anterolaterally, the squamosal dorsolaterally and posterodorsally, the prootic anteromedially, the opisthotic posteromedially, the epipterygoid anteroventromedially, and the pterygoid medially.

As in all turtles, the quadrate of AMNH FR 6015 forms a large funnel-shaped lateral middle ear chamber called the cavum tympani, which in trionychids is anteroposteriorly elongated. The anterior margin of the cavum is entirely formed by the quadrate, as the quadratojugal articulates anteriorly with the quadrate, without overlapping the anterolateral margin of the quadrate by much. The dorsal and posterior margins of the cavum tympani are, however, formed by the squamosal which caps the quadrate (Fig. 7). The incisura columellae auris is enclosed within a canal in AMNH FR 6015 (Fig. 7), as in all trionychids. In some trionychids, a sharp-edged crest that diverges posteroventrally from the incisura columellae auris is developed, such as in the cyclanorbines *Lissemys punctata* and *Cyclanorbis senegalensis*, but also in *Amyda cartilaginea*. In AMNH FR 6015, such a crest is absent (Fig. 7), although this area of the cavum tympani is slightly elevated, similar to the conditions in *Apalone spinifera* or *Pelodiscus sinensis*. The posteroventral margin of the cavum tympani is characterized by a notch that interrupts the margin of the cavum tympani, which serves as an attachment for the tympanic membrane. This notch is situated between the posteroventral squamosal contact and the articular process (Figs. 7, 8). This notch forms an opening from within the cavum tympani to the posterior quadrate surface for the Eustachian tube and a fine ridge on the posterior surface indicates the former course of this structure along the quadrate.

Ventrally to the Eustachian notch, the quadrate has a peculiar, lip-like process that projects posteriorly off the base of the articular process, and which is ventrally recurved along its outer margin (Fig. 8). Similar ridges are found in thalassochelydians (Anquetin et al., 2015, 2017; Evers & Benson, 2019; Evers & Joyce, 2020), where they have been termed the “infolding ridge” of the quadrate (Anquetin et al., 2015). Morphologically, there is no difference between the ridges of thalassochelydians and AMNH FR 6015, and so we also apply the term “infolding ridge” here. All examined trionychids have a minor ridge in this area, but it is nowhere as protruding as in AMNH FR 6015, and not posteriorly extensive enough to even show a recurved edge. A clear exception is *Gilmoremys lancensis*, where the ridge is clearly developed, although it has not been described in previous work on that taxon (Joyce & Lyson, 2011; Joyce et al., 2016). The ridge may therefore be a local synapomorphy for plastomenids.

The articular process of the quadrate barely extends ventrally below the level of the cavum tympani (Figs. 7–8). The margin of the infolding ridge continues anterolaterally above the articular surface. The articular facet itself is transversely concave. Its lateral part is anteroposteriorly somewhat broader than the medial part (Fig. 3B).

Anteromedially, the quadrate has an extremely short epipterygoid process (Fig. 7). However the quadrate process of the epipterygoid is elongate, and therefore the contact between the quadrate and epipterygoid is present. The quadrate of AMNH FR 6015 forms a small part of the posterior margin of the trigeminal foramen, between the epipterygoid and prootic (Fig. 7). The anteroventral surface of the quadrate, lateral to the prootic contact, is strongly inclined anterodorsally so that it overhangs the adductor fossa, forming a ventrally exposed surface, as in other trionychids (Fig. 3B). This is a consequence of the extremely elongate otic capsule and temporal region. On the other hand, the quadrate is reduced to a sinuous, mediolaterally thin string of bone in the floor of the temporal fossa (Fig. 3A), as is common in trionychids. Wedged between the quadratojugal and prootic, the quadrate is nearly excluded from the processus trochlearis oticum, of which the prootic forms approximately 80% (Figs. 3A, 5A–B, 7). The foramen stapedio-temporale is extremely small in comparison to other trionychids, which are generally already considered to have small stapedial foramina among turtles (Albrecht, 1967, 1976), indicating a secondary reduction of the canal similar to that seen in kinosternoids (e.g., Albrecht, 1967; Rollot et al., 2021a). The foramen is present on both sides of AMNH FR 6015 and the respective canal can be easily traced through the slice data, leading posteroventrally into the cavum acustico-jugulare (Figs. 3A, 5C). The canal and foramen are completely formed between the prootic and quadrate.

## Epipterygoid

The two epipterygoids are well-preserved and complete in AMNH FR 6015, forming as large and separate ossifications (Figs. 3D, 5C–D, 7). In cyclanorbines, the epipterygoids fuse with the pterygoids during early ontogeny (Meylan, 1987). This is not only the case in old and large individuals, as for example seen in our specimen of *Lissemys punctata*, in which there is already no trace of a separate epipterygoid, despite its relatively small head size of 51 mm length. Among trionychines, the epipterygoids are less often fused with the pterygoid, although this may happen in old individuals (Meylan, 1987).

The epipterygoid of AMNH FR 6015 is a laminar, mediolaterally thin bone with three clearly developed processes (Fig. 7). The largest process is the posterior process, which conveys approximately 60% of the total length of the element. The posterior process forms the ventral margin of the trigeminal foramen (Fig. 7), but extends slightly posterior to the end of the foramen as well, where it contacts the quadrate. At the level of the trigeminal foramen, the posterior process lies against a very subtle and low crista pterygoidei of the pterygoid (Fig. 5B). Anterior to the trigeminal foramen, the epipterygoid forms a dorsal process, which frames the anterior margin of the trigeminal foramen and contacts the prootic dorsally and the parietal anteriorly (Fig. 7). With its lateral inclination, the dorsal epipterygoid process contributes to the bulbous cavum epiptericum (Fig. 5B), which is principally formed by the prootic (see below). Anterior to the level of the dorsal process, the epipterygoid has an anterior process, which forms part of the secondary lateral wall of the braincase and bridges a gap between the descending process of the parietal and the pterygoid-palatine region. The anterior epipterygoid process contacts the palatine, and lies laterally against it with its anterior tip (Fig. 7). We found similar morphologies and contacts in *Apalone spinifera* and *Amyda cartilaginea*. However, our examined specimens of *Pelodiscus sinensis* and *Chitra chitra* have distinct epipterygoid morphologies: in *Pelodiscus sinensis*, the element is a small palate at the ventral margin of the trigeminal foramen, but without any of the above-described processes. In *Chitra chitra*, the epipterygoid is slender and almost rod-like, and seems to frame the entire ventral margin of the trigeminal foramen, but without a developed dorsal process. See Meylan (1987) for further variation in epipterygoid morphology.

## Pterygoid

The pterygoids are preserved and near complete in AMNH FR 6015. Although heavily fractured, especially on the right side, their full morphology can be appreciated (Figs. 3B, 5B, 7, 8). The pterygoid is anteroposteriorly elongated and is mediolaterally narrow (Fig. 3B). Although the pterygoid of AMNH FR 6015 is involved in the formation of many cranial structures, as in all turtles, its morphology is comparatively simple due to a strongly reduced crista pterygoidei and external pterygoid process. It mainly consists of a horizontal plate that can be roughly divided into an anterior process, a central portion, and a posterior process. The elongate anterior process contacts the maxilla, jugal, and palatine (Fig. 3A), the central portion contacts the parabasisphenoid medially and the quadrate laterally (Fig. 3B), and the short, but broad posterior process contacts the basioccipital posteromedially (Fig. 3B) and the epipterygoid dorsally (Fig. 7). A minute point contact with the opisthotic may have existed just lateral to the posterior jugular foramen (Fig. 8B). On the dorsal surface, the low crista pterygoidei contacts the epipterygoid, but not the parietal (Fig. 7). A broad dorsal contact furthermore exists with the prootic (Fig. 5B). An interpterygoid contact is absent (Fig. 3B), as is typical for trionychids.

The anterior process of the pterygoid of AMNH FR 6015 reaches the posterior end of the triturating surface of the maxilla and forms the lateral margin of the subtemporal fossa (Fig. 3B). As in other trionychids, a true external process is absent in AMNH FR 6015. Instead, the lateral pterygoid margin is gently dorsoventrally expanded at the contact with the posterior end of the medial jugal process to form a low vertical plate. The lateral margin of the pterygoid is damaged posterior to the vertical plate, but the two sides combined suggest that this margin was shaped into a bladelike bony ridge with a convex outline onto which the pterygoideus musculature articulated (Figs. 3B, 7), much as in recent *Apalone spinifera* (FMNH 22178). Although not nearly as strong as in those taxa, this ventrally downcurved and laterally expanded margin of the central part of the pterygoid is similar to that of the pterygoid flange of podocnemidids (Gaffney, 1979; Gaffney et al., 2011). This morphology of the pterygoid in AMNH FR 6015 results in a transversely concave shape of this part of the roof of the mouth.

The pterygoid’s central portion is mediolaterally narrowest at the level of the articular processes of the quadrate, where the parabasisphenoid is laterally slightly expanded (Fig. 3B). This area also marks the beginning of the posterior process of the pterygoid, which extends posteriorly to reach the base of the basioccipital tubercles (Figs. 3B, 8B) formed by the basioccipital. The ventral surface of the posterior pterygoid process is simple in AMNH FR 6015. A deep pterygoid fossa is absent. A short ridge of the pterygoid extends posteromedially from the lateral pterygoid margin just anterior to the contact with the articular process of the quadrate and toward the large foramen posterius canalis carotici interni. As in other trionychids, this entry foramen for the internal carotid artery is fully enclosed within the pterygoid, and is positioned centrally in the posterior process at the level of the basioccipital-basisphenoid suture, posterior to the level of the quadrate articular process, but anteriorly removed from the margin of the fenestra postotica (Fig. 3B). The pterygoid forms the entire ventral margin of this large aperture, which leads into the cavum acustico-jugulare. The ventral surface of the posterior pterygoid process slopes slightly medioventrally toward this margin, so that the foramen posterius canalis carotici interni opens in a posterior direction (Fig. 8B), although it is clearly visible in ventral view (Fig. 3B).

Due to the sloping, elevated pterygoid margin of the fenestra postotica, the contact surfaces between the pterygoid with the exoccipital and with the basioccipital are nearly vertically oriented (Fig. 8). The pterygoid wraps around the lateral margin of the basioccipital and has a broad contact with the foot of the ventral exoccipital process below the foramen jugulare posterius (Fig. 8). A small contact with the opisthotic is inferred in this region (see opisthotic, below), but breakage and slight disarticulation across the fractures makes it hard to ascertain this.

The dorsal surface of the posterior pterygoid process forms the floor of the cavum acustico-jugulare. This area is heavily damaged on both sides of the fossil. However, the pterygoid is clearly excluded from the posterior part of the canalis cavernosus, which opens anteriorly from the cavum acustico-jugulare between the prootic and quadrate. The pterygoid only forms the floor of the canalis cavernosus in its anterior half. The canalis cavernosus extends approximately parallel to the canalis caroticus internus, which traverses the pterygoid in a more medial position. Both canals are separated by a mediolaterally thin wall of bone (Fig. 5A), which is interrupted twice. First, at the level of where the canalis nervus facialis traverses the prootic, there is a short vertical opening between the carotid canal and the canalis cavernosus for the vidian branch of the facial nerve, called the canalis pro ramo nervi vidiani. And second, at the level of the foramen cavernosus (i.e., the anterior end of the canalis cavernosus), where the internal carotid canal produces a lateral opening toward the sulcus cavernosus, which can be identified as a (short) canalis caroticus lateralis (sensu Rollot et al., 2021a; Fig. 5A). This opening is, in turtles, generally associated with the palatine artery, which splits from the internal carotid artery in this area and extends into the sulcus cavernosus. In trionychids, however, the artery that traverses the above-described opening provides blood to the mandible and is thus called the mandibular artery (Albrecht, 1967). Rollot et al., (2021a) interpreted the trionychid mandibular artery as a ‘repurposed’ palatine artery. Potential evidence that the extant trionychid arterial system was also present in AMNH FR 6015 comes from the extreme narrow shape of the canalis cavernosus. The narrowness indicates that only the lateral head vein traversed the canalis cavernosus, as this structure is usually much broader and often partially compartmentalized in turtles in which the mandibular artery extends through the canalis cavernosus alongside the lateral head vein. It is also unlikely that the mandibular blood supply was via the stapedial artery in AMNH FR 6015, as the stapedial canal is also strongly reduced. Thus, the available information suggests that the mandibular blood supply was as in extant trionychids, implying the presence of a repurposed palatine artery.

At the position of the canalis caroticus lateralis, the other, medial branch of the internal carotid artery enters the sella turcica of the basisphenoid as the cerebral artery through another short canal, the canalis caroticus basisphenoidalis (sensu Rollot et al., 2021a; Fig. 5A). Whereas this canal is entirely within the parabasisphenoid in most turtles, this canal is formed between the trabeculae of the parabasisphenoid dorsally, and the pterygoid ventrally in AMNH FR 6015 and other trionychids (Fig. 5C; see “Parabasisphenoid”).

The widely spaced trabeculae of the parabasisphenoid lie on the dorsal surface of the pterygoid in the trigeminal region of the braincase. They form the medial wall to a sulcus cavernosus, that is otherwise formed by the pterygoid (Fig. 5A, B). As in trionychids more generally, the sulcus cavernosus is otherwise not pronounced as a trough within the pterygoid surface. However, it is laterally bordered by an extremely shallow crista pterygoidei, which extends for the posterior two-thirds of the length of the trigeminal foramen (Fig. 5B). The crista serves as an articular facet for articulation with the posterior epipterygoid process, which lies laterally against it.

## Supraoccipital

The supraoccipital of AMNH FR 6015 is relatively complete and intact with the exception of the tip of the supraoccipital crest, which is missing, and a horizontal break that extends through the entire length of the bone (Figs. 3A, C–D, 5C and 8). The supraoccipital contacts, from anterior to posterior, the parietal, the prootic, the opisthotic, and the exoccipital.

The main part of the supraoccipital forms the roof of much of the braincase. Each side of the bone has a lateroventrally diverging process that contacts the prootic anteriorly, opisthotic posteriorly, and exoccipital even further posteriorly. The area between right and left processes is strongly concave, so that anterior portions of the bone have an inverted, “U”-shaped cross-section. The processes become less strongly arched at the exoccipital contact (Fig. 8A), where the supraoccipital closes the foramen magnum dorsally. The lateral processes of the supraoccipital are lateroventrally deepest at the triple-junction with the prootic and opisthotic, but become laterally expanded a second time at the triple junction with the opisthotic and exoccipital (Fig. 3A). The anterior most of these lateroventral expansions houses the common crus portion of the endosseous labyrinth, and the anterolateral and posterolateral margins facing the prootic and opisthotic, respectively, also house parts of the anterior and posterior semicircular canals. The bone surface between the endosseous labyrinth and temporal fossa is extremely thin. AMNH FR 6015 shows nearly symmetrical damage along the posterior semicircular canal at the interface of the supraoccipital and opisthotic (Figs. 3A and 5B). This is specifically mentioned here because the rough symmetry between breaks on either side of the skull make these features appear as foramina on a first glimpse. Medially, the supraoccipital processes extend relatively deeply, reducing the size of the hiatus acusticus between the endosseous labyrinth and braincase proper. As a consequence, the foramen aquaducti vestibuli is fully ossified within the medial wall of the braincase as formed by the supraoccipital.

This anterior section of the supraoccipital extends somewhat underneath the parietals, which also frame the anterior section of the crista supraoccipitalis, which starts as a dorsal crest in the anterior region of the supraoccipital. More posteriorly, beyond the level of the foramen magnum, the crista supraoccipitalis is a robust vertical ridge which becomes dorsoventrally lower toward its posterior tip, which is not fully preserved. The ventral surface of the crista supraoccipitalis is broadened as typical in trionychids, contributing to an inverse “T”-shaped cross section (Fig. 8).

## Exoccipital

The exoccipitals are near complete in AMNH FR 6015, except for the posteriormost portion of their ventral bases, which contribute to the occipital condyle. Additionally, the exoccipitals are both affected by fractures near their bases (Fig. 8). The exoccipital contacts the supraoccipital dorsally, the opisthotic anterolaterally, the pterygoid ventrolaterally, and the basioccipital posteroventrally. The inter-exoccipital contact that is variably present in turtles along the dorsal surface of the occipital condyle is not preserved in AMNH FR 6015 as the occipital processes are broken off, but the facets for the right and left exoccipital meet in a median ridge on the basioccipital, indicating that the contact was there (Fig. 8).

The ventral part of the exoccipital forms a robust base to the remainder of the bone, which is columnar and extends dorsally to the side of the foramen magnum. Anteriorly, the exoccipital footplate forms the ventral and posterior margin of the foramen jugulare anterius between the braincase and the recessus scalae tympani. On the occipital surface of the skull, the exoccipital completely encases the foramen jugulare posterius (Fig. 8), which, consequentially, is distinct from the fenestra postotica. Below the level of the foramen jugulare posterius, the exoccipital forms a shallow fossa in which the foramina for the hypoglossal nerve rami are expected, but a horizontal fracture erased the traces of these canals on both sides of the skull, although traces of the foramina are present (Fig. 8B). The posterior surface of the exoccipital has a short vertical ridge immediately lateral to the foramen magnum (Fig. 8), which is also present in extant trionychids (e.g., *Apalone spinifera*; *Lissemys punctata*), although they can be subtle (e.g., *Cycloderma frenatum*, *Cyclanorbis senegalensis*).

## Basioccipital

The basioccipital of AMNH FR 6015 is missing its posterior end, which would have formed the ventral part of the occipital condyle, but is otherwise complete (Figs. 3B and 8). The bone contacts the parabasisphenoid anteriorly, the pterygoid laterally, and the exoccipital dorsally. Additionally, there perhaps was a contact with the opisthotic along a recurved process of that bone, which extends posteriorly in the braincase wall to contact the exoccipital and exclude the basioccipital from the margin of the foramen jugulare anterius. At present, there is a small gap between these bones, and it is not entirely clear if this is the result of crushing. This possible contact is only visible on the right side of the specimen, as the left opisthotic is ventromedially damaged.

As preserved, the basioccipital is a roughly pentagonal block of bone in the posterior part of the floor of the basicranium (Fig. 3B). The articulation with the parabasisphenoid is reinforced by dorsal and ventral lappets of bone from the latter, which under- and overlap the anterior margin of the basioccipital. This same mode of articulation seems to be present in other trionychids as well, including *Apalone spinifera*. As in other trionychids, the lateral margins of the basioccipital of AMNH FR 6015 are also underlapped by the extended posterior pterygoid processes (Fig. 3B). Nonetheless, the basioccipital extends posteriorly beyond the level of the pterygoid and forms posterolaterally distinct basioccipital tubercles (Figs. 3B and 8). The ventral surface between these tubercles is gently recessed. As is generally the case in trionychids, each tubercle forms an extended platform dorsally, which is slightly recessed to a fossa below the exoccipital (Fig. 8).

Posteriorly, the basioccipital forms a median occipital process for the occipital condyle, the posterior tip of which is broken in AMNH FR 6015 (Fig. 8). The occipital process likely had a relatively elongate neck compared to other cryptodires (Fig. 3B), as is common in trionychids. Dorsally, the process shows clear facets for the occipital processes of the exoccipitals. As these facets meet along the process, it is clear that the exoccipitals would have contacted one another medially, again as is typical for trionychids (Fig. 8).

The dorsal surface of the basioccipital does not bear any salient features. A crista dorsalis basioccipitalis is absent. The lateral margins of the basioccipital are relatively high toward the foramen jugulare anterius, but do not reach the margin of this opening.

## Prootic

Both prootics are complete and well-preserved in AMNH FR 6015 (Figs. 3A, 5A, C–D and 7). As in turtles more generally, the prootic is a complex bone that contacts the parabasisphenoid, quadrate, pterygoid, epipterygoid, opisthotic, parietal, and supraoccipital. In AMNH FR 6015, a contact with the epipterygoid is also present (Fig. 7).

The prootic is an anteroposteriorly elongate element in trionychids, including AMNH FR 6015, which contributes to the anteroposteriorly long otic capsule and temporal fossa (Fig. 3A). The posterior part has a ventral process, which contacts the parabasisphenoid and pterygoid medially to the canalis cavernosus, and a central block that contains the prootic part of the endosseous labyrinth. This part of the otic capsule lies medially against the quadrate, roofing the canalis cavernosus, of which the quadrate forms the lateral wall. The canalis stapedio-temporale lies between the quadrate and the otic capsule part of the prootic, and extends anterodorsally from the cavum acustico-jugulare to open at the floor of the temporal fossa. The canal and respective foramen stapedio-temporale are reduced in diameter, even more so than in other trionychids.

The ventral process of the prootic is mediolaterally traversed by the canalis nervus facialis (sensu Rollot et al., 2021a), which extends from the fossa acustico-facialis on the medial surface facing the braincase into the canalis cavernosus, and which would have carried the facial nerve. The facial nerve canal organization (including the presence of a canalis pro ramo nervi vidiani in the pterygoid, just ventral to the lateral foramen of the canalis nervus facialis) indicates that the geniculate ganglion of AMNH FR 6015 was positioned within the canalis cavernous, which compares to some, but not all trionychids (Rollot et al., 2021a). While the vidian branch of the facial nerve took the course of the canalis pro ramo nervi vidiani, there is no osteological correlate for the hyomandibular branch, which would have traversed posteriorly through the canalis cavernosus. From the fossa acustico-facialis, additional openings are directed into the prootic part of the endosseous labyrinth cavity. These are for the acoustic nerve rami. The thin bone of the left prootic is broken in parts along the original position of the acoustic nerve foramina in AMNH FR 6015, but in the right prootic, two separate openings remain, indicating that at least two rami entered through separate foramina. Within the labyrinth cavity itself, it is noteworthy that the prootic contributes to the formation of the lateral semicircular canal. This canal is usually only formed by the opisthotic in cryptodires, with several exceptions including *Pelodiscus sinensis* (Evers & Benson, 2019). However, no other extant trionychid species shows this feature according to our observations. The feature is interesting, because the prootic contribution to the lateral semicircular canal is typical of pleurodires, and the distribution of this state among trionychians may influence its optimization for the plesiomorphic state of crown-turtles. Although there is some damage to the skull around the fenestra ovalis on both sides, it seems that the prootic of AMNH FR 6015 lacks a posterior footplate, as in *Apalone spinifera*. Overall, it seems that the fenestra ovalis was ventrally un-ossified in AMNH FR 6015, as in other trionychids, but it is hard to ascertain this as the posterior margin of the fenestra ovalis along the opisthotic is damaged on both skull sides (see opisthotic). A fossa for the pericapsular recess is absent in AMNH FR 6015.

Our µCT scans of AMNH FR 6015 and other trionychids show that the anteroposteriorly elongate prootic shape that differs from other turtles is achieved by an anterior elongation. In most turtles, the prootic barely expands anteriorly beyond the foramen cavernosum or the trigeminal foramen. In AMNH FR 6015 and the fully segmented *Apalone spinifera* (Evers & Benson, 2018), the prootic has a large anterior process, which extends deeply into the subtemporal fossa, bringing the processus trochlearis far forward anteriorly (Figs. 3A, 5B and 7). Simultaneously, the anterior process extends along the entire length of the trigeminal foramen, and forms its posterior and dorsal margin until it contacts the dorsal epipterygoid process, which forms the anterior margin of the foramen (Fig. 7). The trigeminal foramen itself is anteroposteriorly elongate and oval, possibly to release the mandibular artery alongside the trigeminal nerve branches, which are large in turtles (Evers et al., 2019b). The anterior process of the prootic of AMNH FR 6015 is strongly laterally deflected from the posterior, otic part of the bone (Fig. 5B). In addition, the medial surface is very strongly concavely excavated (Fig. 5A, B). Both features result in an inflation of the interior braincase space at the level of the trigeminal foramen, which can also be appreciated in endocasts of other trionychids, such as *Pelodiscus sinensis* (Werneburg et al., 2021a). This part of the braincase endocast corresponds to the cerebral hemispheres dorsally, and the cavum epiptericum for the position of the trigeminal ganglion ventrally. Stained µCT-scans of the extant trionychid *Apalone spinifera* (YPM VZ-1270; Ferreira et al., 2022) show that the cerebral hemispheres of the soft tissue brain are indeed expanded in trionychids relative to other turtles, indicating that the expanded inter-prootic space in AMNH FR 6015 was also likely filled nearly entirely with brain tissue.

The lateral side of the anterior process is developed to form a crescentic, transversely dorsally concave processus trochlearis oticum (Figs. 3A, 5A, B and 7). The parietal, which overlies the prootic mediodorsally, contributes somewhat to the concave facet of this process, as does the quadrate laterally. However, both contributions are small, and the prootic forms the majority of the width of the process, which is narrow to begin with.

## Opisthotic

The two opisthotics are incompletely preserved in AMNH FR 6015 (Figs. 3A, 5B and 8). On both sides, parts of the paroccipital process and processus interfenestralis are broken. In addition, some of the dorsoventrally thin bone above the posterior semicircular canal is broken nearly symmetrically on both sides of the skull, giving the superficial illusion of a foramen between the opisthotic and supraoccipital (Fig. 5B).

The opisthotic of AMNH FR 6015 contacts the prootic anteriorly, the supraoccipital mediodorsally, the exoccipital posteromedially, the quadrate laterally, and the squamosal posteriorly along the paroccipital process. A point contact with the pterygoid seems likely along the bony bar that separates the fenestra postotica from the foramen jugulare posterius (Fig. 8).

The anterior part of the opisthotic is internally hollowed by the cavities for the endosseous labyrinth. The medial wall of the labyrinth is well ossified in AMNH FR 6015. As a consequence, the foramen internum nervi glossopharyngei is fully enclosed in bone (Fig. 5C). In the right opisthotic, the respective canal and lateral foramen for the glossopharyngeal nerve are also preserved, showing that the canal did not traverse the labyrinth cavity, but passed just ventrally to its inner surfaces through the processus interfenestralis. *Apalone spinifera* has the more common condition of the nerve passing through the labyrinth cavity, before exiting laterally into the cavum acustico-jugulare. This minor difference in canal path has not generally been paid attention to, and so the potential systematic relevance should be explored in the future.

The processus interfenestralis is incompletely preserved on both sides. However, a notch for the fenestra perilymphatica is preserved in the right opisthotic. This side also shows that the foramen jugulare anterius was fully ossified between the opisthotic and exoccipital. The left opisthotic preserves most of the part of the processus interfenestralis that extends to the fenestra ovalis. This part of the process is an extremely thin vertical sheet of bone, and it seems unlikely that it extended all the way to the floor of the basicranium or was associated with an extended footplate. Evidence for this suspicion comes from the fact that there are no larger broken pieces floating within the cavum acustico-jugulare/labyrinth part of the skull.

Posteriorly, the opisthotic forms the dorsal margin to the fenestra postotica, which opens posterolaterally from the skull (Fig. 8). Along the medial margin of this fenestra, the opisthotic sends out a ventral, pointed process that likely contacted the pterygoid (Fig. 8). This ventral pterygoid process lies laterally against a thin strut of the exoccipital bone that surrounds the foramen jugulare posterius (Fig. 8). There is a sharp-edged ridge that extends from the pterygoid process of the opisthotic dorsolaterally onto the paroccipital process, forming its sheeted posterior margin. The paroccipital process is posterodorsally and laterally directed, and contacts the squamosal in a horn-like posterior process that would have likely been larger in its original state (Fig. 8).

## Parabasisphenoid

The parabasisphenoid is almost completely preserved in AMNH FR 6015. It is elongate anteroposteriorly (Fig. 3B). As in other trionychids, its ventral exposure is mediolaterally broad posteriorly near the basioccipital contact, narrows anteriorly between the central part of the pterygoids, and then becomes slightly broader again just before contacting the palatines anteriorly (Figs. 3B and 4C). Dorsally, within the braincase, the parabasisphenoid is much broader mediolaterally than expected from ventral view alone (Fig. 9B), as the pterygoids underlie the lateral margin of the bone. The dorsal surface of the parabasisphenoid forms only a very shallow cup for the midbrain region, and the lateral margins that contact the prootic are low (Fig. 5A, C, D). There is no sign of the basis tuberculi basalis. The parabasisphenoid forms flat projections that posteriorly sandwich the anterior parts of the basioccipital from above and below.

The dorsum sellae is dorsoventrally low, overhanging a low but mediolaterally broad sella turcica (Figs. 5A and 9). The anterior surface of the sella turcica is flat and without vertical ridge. Clinoid processes are entirely absent in AMNH FR 6015, as in most other trionychids (e.g., *Lissemys punctata*, *Cyclanorbis senegalensis*, *Amyda cartilaginea*, *Apalone spinifera*, *Pelodiscus sinensis*, but not *Chitra chitra*, which retains dorsally spiked clinoid processes). Interestingly, AMNH FR 6015 lacks a fully ossified nerve canal for the abducens nerve. Instead, there is a fine groove on the dorsal surface of the parabasisphenoid, which extends from the midbrain cup anterolaterally over the base of the trabecula. As the abducens nerve foramen usually exists dorsal to the trabecula, this position is topologically congruent with an interpretation as a groove for the abducens nerve. An abducens nerve groove is unusual for turtles, but has previously been described for *Sandownia harrisi* (Evers & Joyce, 2020, a thalassochelydian turtle which was originally identified as a “trionychoid” (Meylan et al., 2000). Besides this groove, these two taxa share a lot of otherwise unusual parabasisphenoid features, including the spaced trabeculae and foramina for the cerebral artery that are not fully formed by the parabasisphenoid alone (see Evers & Joyce, 2020). Within trionychids, all digitally examined trionychines (*Pelodiscus sinensis*, *Amyda cartilaginea*, *Apalone spinifera*, *Chitra chitra*) have clear abducens canals. However, the examined cyclanorbines (*Lissemys punctata*, *Cyclanorbis senegalensis*) lack the canal, just like AMNH FR 6015.

The cerebral artery of AMNH FR 6015 enters the sella turcica via a short, foramen-like canalis caroticus basisphenoidalis (Fig. 5A). In most turtles, the anterior opening foramen into the sella turcica, the foramen anterius canalis carotici basisphenoidalis, is entirely enclosed by the parabasisphenoid. In AMNH FR 6015 and other trionychids, this differs and the foramen is floored ventrally by the pterygoid (Fig. 5A, C). The base of the parabasisphenoid trabecula forms the dorsal roof of the passage, but the trabeculae are so far spaced from the midline (Fig. 5A, B) that they do not lie on the lateral margins of the rostrum basisphenoidale (as in turtles more generally), but on the laterally adjacent pterygoids. As a consequence, the foramina carotici canalis basisphenoidalis are also extremely broadly spaced. The rostrum basisphenoidale is flat and narrows anteriorly, so that is assumes a triangular shape in dorsal view, which overlaps the palatines within the floor of the cavum cranii (Fig. 5A, C, D).

## Stapes

No stapes is preserved in AMNH FR 6015.

## Endosseous labyrinth

The labyrinth of AMNH FR 6015 is a low pyramidal structure with three partly distinguishable semicircular canals (Evers et al., 2022b; Fig. 10). In AMNH FR 6015, the anterior semicircular canal is notably longer than the posterior semicircular canal (Fig. 10A, B). To some degree, this mimics the morphology of the bones of the otic capsule, in which the prootic is notably elongated (see above). This reinforces ideas that the braincase morphology itself has a strong influence on the morphology of the vertebrate labyrinth by imposing spatial constraints (Benson et al., 2017; Bronzati et al., 2021; Evers et al., 2022b).

All semicircular canals of AMNH FR 6015 are relatively slender (Fig. 10). Although the posterior semicircular canal appears short, the membranous duct would have extended ventrally to its intersection with the lateral semicircular canal through the secondary common crus (Evers et al., 2019b). The posterior end of the labyrinth is relatively strongly bulged, and this bulge extends relatively deep below the level of the lateral semicircular canal (Fig. 10A, D). The same morphology can be seen in other models of published trionychid endosseous labyrinths (Evers et al., 2019b, 2022b; Lautenschlager et al., 2018), and indicates that the posterior semicircular duct curved around the lateral semicircular duct in a comparatively wide arch (Ferreira et al., 2022).

The ampullae of the semicircular canals are only weakly demarked in AMNH FR 6015, as is generally the case in turtles (Evers et al., 2019b). Only the lateral ampulla appears relatively clearly as an inflated region at the anterior end of the respective semicircular canal (Fig. 10B). The common crus is low, contributing to the overall low aspect ratio of the labyrinth in AMNH FR 6015 (Fig. 10A). The short canal for the endolymphatic duct through the foramen aquaducti vestibuli in the supraoccipital appears as a small finger in the vestibular area below the posterior part of the anterior semicircular canal (Fig. 10A, C). The imprints of the fenestrae ovalis and perilymphatica are incomplete due to incomplete preservation of the opisthotic (Fig. 10A, D).

## Mandible

The mandible of AMNH FR 6015 is well-preserved in its anterior half, but the posterior half is strongly fractured and incompletely preserved (Fig. 11). The left mandibular ramus preserves the articulation surfaces for the cranium and the retroarticular process, but the ventral margin is broken. On the right side, the retroarticular process is completely broken off but the ventral margin of the dentary is preserved. Nearly all bone fragments found in the scan can be confidently assigned to a particular mandibular bone (i.e., dentary, surangular, coronoid, prearticular, angular articular) based on comparisons with extant trionychid mandibles (Evers et al., 2022a). The postcoronoid bones are unfused, as is generally the case in cryptodires (Evers et al., 2022a). A splenial is clearly absent, as there are no mandibular pieces preserved in the area in which a splenial could be expected. The absence of the splenial is also expected, given that trionychians as well as fossil outgroups of the clade (e.g., *Adocus lineolatus*: Meylan & Gaffney, 1989) lack a splenial.

## Dentary

As in most turtles (e.g., Gaffney, 1979), the left and right dentary are completely fused in AMNH FR 6015 (Fig. 11E). The symphysis is extremely elongated in AMNH FR 6015 to form an expanded, spatulate area (Fig. 11E). Whereas right and left triturating surfaces of turtles are usually only in contact with one another at the tip of the jaw (Evers et al., 2022a), the triturating surfaces of AMNH FR 6015 are nearly conjoined along their entire anteroposterior extent, posteriorly nearly reaching the level of the coronoid (Fig. 11E). Neither the elongation of the symphysis, nor the complete fusion of the right and left triturating surfaces along the midline is known from any extant trionychid (Evers et al., 2022a). Fused triturating surfaces appear, however, in some other fossil turtles, notably the thalassochelydian *Sandownia harrisi* (Evers & Joyce, 2020; Meylan et al., 2000) and the plastomenid *Gilmoremys lancensis* (Joyce & Lyson, 2011). In AMNH FR 6015, the symphyseal triturating surface area is posteriorly ever so slightly raised to a dorsal symphyseal mount (Fig. 11C, E), but anteriorly this makes way to a central depression that is laterally defined by a low labial ridge (Fig. 11C, D). In *Gilmoremys lancensis*, the entirety of the preserved triturating surface area is gently depressed (Joyce & Lyson, 2011). The outside of the blunt labial ridge of AMNH FR 6015 is lined by a row of neurovascular foramina, as is also the case in extant trionychids (Evers et al., 2022a). The dentary is dorsoventrally low anterior to the coronoid region of the mandible (Fig. 11A, B), and a second row of neurovascular foramina is apparent on the lateral dentary surface.

At the level of the coronoid process, the dentary becomes dorsoventrally much taller. It forms an anterodorsally ascending process along the anterior margin of the coronoid (Fig. 11B), a feature that is exclusively shared between extant trionychids and *Carettochelys insculpta* among extant turtles (Evers et al., 2022a). This process is only preserved on the right side of AMNH FR 6015, but a facet for the process along the left coronoid indicates its former presence on both sides. Posterior to the ascending dentary process, the lateral surface of the dentary shows a shallow adductor fossa (Fig. 11B). This depression is inconspicuous, as in extant trionychids (Evers et al., 2022a), but different from the deep fossa observed in *Carettochelys insculpta*. Within the anterior margin of the adductor fossa, AMNH FR 6015 has a foramen dentofaciale majus that is much larger than those of any extant trionychid (Fig. 11A, B), but smaller than the extremely large foramen known from *Carettochelys insculpta* (Evers et al., 2022a). Among trionychids, the foramen can be completely reduced, as in cyclanorbines or *Chitra chitra* among trionychines (Evers et al., 2022a).

Posteriorly, the dentary is developed as a mediolaterally thin sheet of bone that laterally abuts against the surangular. At its ventral margin, the bone is thicker and extends posteriorly approximately up to the level of the mandibular articulation (Fig. 11A–B). The exact extent is difficult to access due to breakage.

On the medial surface, the dentary bears a large foramen alveolare inferius ventral to the level of the coronoid (Fig. 11C–D). Although the prearticular is badly preserved, it seems that this foramen was exposed in medial view, as is also the case in extant trionychids and *Carettochelys insculpta* (Evers et al., 2022a). The sulcus Meckelii is a shallow but dorsoventrally tall depression on the medial dentary surface in AMNH FR 6015. The right and left sulci converge in the symphyseal jaw area into a central depression that invades the symphyseal spatula of AMNH FR 6015 (Fig. 11C, D).

## Surangular

The surangular of AMNH FR 6015 is completely preserved on the left side (Fig. 11A, E, F), but is heavily fractured, like all of the postcoronoid bones. The right surangular lacks its posterior end (Fig. 11B, E, G). The surangular of turtles is a vertically oriented, mediolaterally thin sheet of bone in the posterior part of the mandible (e.g., Gaffney, 1979). In AMNH FR 6015, much of its lateral surface is exposed posterior to its contact of the dentary (Fig. 11A, B). The dentary-surangular contact is quite similar to those of extant trionychids (Evers et al., 2022a), suggesting that the area is genuine as preserved. The surangular usually frames the lateral wall of the fossa Meckelii, and contacts the coronoid at the anterior end of the dorsal opening into this space (Evers et al., 2022a; Gaffney, 1979). In AMNH FR 6015, the surangular-coronoid contact is well-preserved on the left side (Fig. 11A, C, E), which shows that a short posterior process of the coronoid along the anteromedial margin of the surangular prevents a surangular-prearticular contact along the anterior margin of the fossa Meckelii (Fig. 11C, E). The preserved bits of the prearticular suggest that the fossa Meckelii and its dorsal opening were mediolaterally narrow (Fig. 11E), as is typical for trionychids (Evers et al., 2022a).

The dorsal margin of the surangular is laterally bulged at the level of the mandibular articulation surface, forming a prominent ectocondylar flange (Fig. 11A–B, E–F), as in trionychians in general (Evers et al., 2022a). The flange forms more than 50% of the mediolateral width of the articulation area (Fig. 11F, G), which is again typical for trionychians (Evers et al., 2022a; Gaffney, 1979; Meylan, 1987). The dorsal surface of the ectocondylar flange of the surangular is gently depressed. The medial margin of the surangular that faces the articular would have likely formed a shallow anterolateral ridge, contributing to a biconcave articulation facet that is also observed in extant trionychians (Evers et al., 2022a).

The foramen auriculotemporalis is strongly reduced in size, as in other trionychids in which there may be several small foramina (Evers et al., 2022a; Meylan, 1987). The foramen of AMNH FR 6015 is positioned posteroventrally to the ectocondylar flange (Fig. 11A–B) and can be differentiated from other small, fracture-related holes in the postcoronoid bones by its association with a small interior canal, which extends anterodorsally through the surangular and into the fossa Meckelii via a medial auriculotemporal foramen (visible in Fig. 11D due to breakage of the prearticular). A dorsal surangular foramen is also present, unlike in other trionychids. This is a small opening anterodorsally to the ectocondylar flange, and connected to the internal auriculotemporal canal (see Evers et al., 2022a for details).

The surangular is posteriorly expanded to form the lateral half of the elongated retroarticular process (Fig. 11E, F), the length of which exceeds the anteroposterior length of the articulation surface immediately anterior to it. Among turtles, such elongated retroarticular processes are only found among trionychians (Evers et al., 2022a; Meylan, 1987). The retroarticular process of AMNH FR 6015, although not completely preserved at its posterior tip, seems to have a broad posterior end (Fig. 11F) similar to those seen in trionychids.

Although the surangular is well-preserved on both sides, there is no evidence of an anteromedially recurved surangular lamina on the medial surface of this bone (see Evers & Benson, 2019; Evers et al., 2022a). In extant trionychids, this lamina often forms a contact for the medially adjacent prearticular. In AMNH FR 6015, it seems that the contact was entirely absent, evidence for which also comes from the articular preservation (see below).

## Coronoid

Both coronoids of AMNH FR 6015 are nearly completely preserved, albeit fractured. The dorsal portion of the left coronoid is attached to the cranium instead of the remainder of the mandible. We therefore digitally re-articulated it with the ventral part of the bone.

The coronoid of AMNH FR 6015 forms a dorsally tall, vaguely triangular coronoid process (Fig. 11A–D), as in all extant trionychians (Evers et al., 2022a; Gaffney, 1979; Meylan, 1987). The coronoid is rigidly connected to the dentary via two anteroventral processes. The lateral one of these is laterally overlain by the anterodorsal process of the dentary (Fig. 11A, B). The medial process deeply slots into a facet of the dentary, and extends to the base of the triturating surface to which it also contributes (Fig. 11C–E). A small contribution to the triturating surface is also seen in extant trionychids, and to a lesser degree in *Carettochelys insculpta* (Evers et al., 2022a). Cyclanorbines have a small coronoid foramen along the medial surface of the anteroventral process of the coronoid. This foramen is absent in AMNH FR 6015, which is also the case in trionychines (Evers et al., 2022a). The lateral surface of the coronoid of AMNH FR 6015 is recessed by the adductor fossa, and a shallow anterodorsally trending adductor fossa ridge is present along the anterolateral margin of the bone (Fig. 11A, B). This ridge is not seen among extant trionychids, but is slightly reminiscent of the strong coronoid adductor fossa ridge of *Carettochelys insculpta* (Evers et al., 2022a).

Posteriorly, the coronoid contacts the surangular lateral to the dorsal foramen into the fossa Meckelii (Fig. 11A, B). A medial contact with the prearticular is not preserved, but was likely present (Fig. 11C), as this is the case in all turtles except some derived chelids (Evers et al., 2022a).

## Prearticular

The prearticular is the most fractured and incompletely preserved bone of the mandible of AMNH FR 6015 (Fig. 11C–D). The left side of the fossil preserves many pieces of the bone in life position (Fig. 11C). As these pieces are predominantly near the bone margin, the shape of the prearticular can be determined surprisingly well. Our interpretation results in a prearticular that is similar to that of other trionychians, particularly in that a posteroventral process of the prearticular is absent (Evers et al., 2022a). The bone seems to form the uni-radiate plate also seen in other trionychians, in which there is an anterodorsal end of the bone that contacts the coronoid medial to the fossa Meckelii and a posterior end that contacts the angular and articular (Fig. 11C).

The anterodorsal half of the prearticular diverges dorsally away from the angular, leaving an acutely triangular opening between the fossa Meckelii and the space between the mandibular rami, the foramen intermandibularis medius of Gaffney (1972, 1979) (Fig. 11C).

In the posterior half of the element, the prearticular contacts the angular in a nearly horizontal suture (Fig. 11C). It is unclear if a posterior intermandibular foramen was present along the angular-prearticular contact. This foramen is often but not ubiquitously reduced in trionychids (Evers et al., 2022a; Meylan, 1987).

The dorsal margin of the prearticular forms the medial border of the foramen in the fossa Meckelii, which must have been mediolaterally narrowed to a slit, as in other trionychids (Evers et al., 2022a; Meylan, 1987). Although a prearticular-surangular contact is often present posterior to the dorsal foramen into the fossa Meckelii among trionychids (Evers et al., 2022a; Meylan, 1987), this does not seem to be the case in AMNH FR 6015: the articular seems to separate both bones clearly on the left side (Fig. 11F–G). The preserved bits of the prearticular along its dorsal margin suggest that the bone was medially slightly flared at the level of the mandibular articulation (Fig. 11F–G) to make room for the articular. This morphology makes it likely that the prearticular was at least minorly included in the formation of the articular surfaces. Possible evidence for this also comes from further posterior sections of the bones: the left side of the fossil clearly demonstrates that the prearticular contributes to the retroarticular process (Fig. 11F). This is largely absent in those extant trionychids in which the prearticular is also excluded from the articulation surface, but present for example in *Carettochelys insculpta*, in which the prearticular contributes to both the mandibular articulation surface and the retroarticular process (Evers et al., 2022a).

## Angular

The left angular of AMNH FR 6015 is nearly complete (Fig. 11C), whereas the right element lacks the posterior end (Fig. 11D). Both elements are heavily fractured. The angular morphology confines to the generalized morphology of this bone in trionychids: the anterior end of the bones is a simple, vertical sheet of bone, and the posterior end is twisted and slightly broadened mediolaterally, forming a horizontally oriented cup-like structure that underlies the articular and forms the ventral margin of the mandibular ramus at the level of the mandibular articulation. The anterior process of the angular is restricted to the medial mandibular surface, and thus ventrally bordered by the dentary (Fig. 11D). This is the same as in extant trionychids (Evers et al., 2022a). Posterior to the foramen intermandibulare medius, the angular contacts the prearticular in an anteroposteriorly trending suture (Fig. 11C). As stated above, the presence or absence of a posterior intermandibular foramen is not entirely clear (see “Prearticular”).

## Articular

The two articulars of AMNH FR 6015 are incompletely preserved (Fig. 11E–G), but left and right elements complement one another. On the left side, the posterior end of the articular that forms the retroarticular process is preserved (Fig. 11F). On the right side, the portion of the articular that forms part of the mandibular articulation and the anterior process is preserved (Fig. 11G). The articular of AMNH FR 6015 likely gives important clues to the ontogenetic stage of the specimen. In the extant trionychian *Carettochelys insculpta*, articular ossification and related morphology changes quite strongly throughout postnatal growth. In hatchlings, the articular is only present as a small ovoid ossification that is positioned medial to the surangular portion of the retroarticular process, but which lacks the central parts of the bone entirely (personal observation SWE on a hatchling specimen, UMMZ herps 123941, of 18 mm skull length). In subadult and adult specimens (USNM 32,796, with 42 mm skull length; NHMUK, 1903.7.10.1, with 104 mm skull length), the articular is fully formed. As the articular of AMNH FR 6015 is completely ossified, the specimen likely corresponds to a mature individual.

As in extant trionychians, the articular of AMNH FR 6015 is a large, anteroposteriorly elongate element in comparison to other turtles (Evers et al., 2022a). Its central portion forms the medial third of the mandibular articulation surface (Fig. 11G), and is dorsally convexly rounded, as in *Carettochelys insculpta* but differing from extant trionychids, in which this surface is flat (Evers et al., 2022a). As the angular of *Carettochelys insculpta* is slightly medially tilted and as the articular is medially bordered by a prearticular with a raised dorsal margin, the combination of these surfaces still results in a concave medial portion of the jaw articulation. This same morphology is seen here in AMNH FR 6015. The ectocondylar flange of the surangular forms the lateral half of the articulation surface (Fig. 11G).

At the anterior end, the articular has a spur-like process that inserts into the gap between prearticular and surangular. Such an anterior process is seen in extant trionychids as well.

The posterior end of the articular of AMNH FR 6015 forms the medial part of the retroarticular process (Fig. 11F), as in trionychians (Evers et al., 2022a; Meylan, 1987). The dorsal surface of the bone is recessed to a shallow fossa. Within this surface, there is no evidence for the presence of a posterior chorda tympani foramen (Fig. 11F). This foramen is located on the dorsal articular surface of the retroarticular process in most turtles (Evers et al., 2022a; Gaffney, 1979), including in cyclanorbines. In trionychines and *Carettochelys insculpta*, however, the foramen is absent from this area of the jaw, and the respective chorda tympani nerve enters the mandible instead on the medial surface of the prearticular, ventral to the mandibular articulation area (Evers et al., 2022a). Unfortunately, this part is badly preserved in AMNH FR 6015 (Fig. 11C), so that the alternative position for the posterior chorda tympani foramen cannot be confirmed. The posterior end of the retroarticular process is not fully preserved (Fig. 11F), but the observable morphology suggests a mediolaterally broad end to the process, that is typical for trionychids.

## Phylogenetic results

**Fig. 12:**
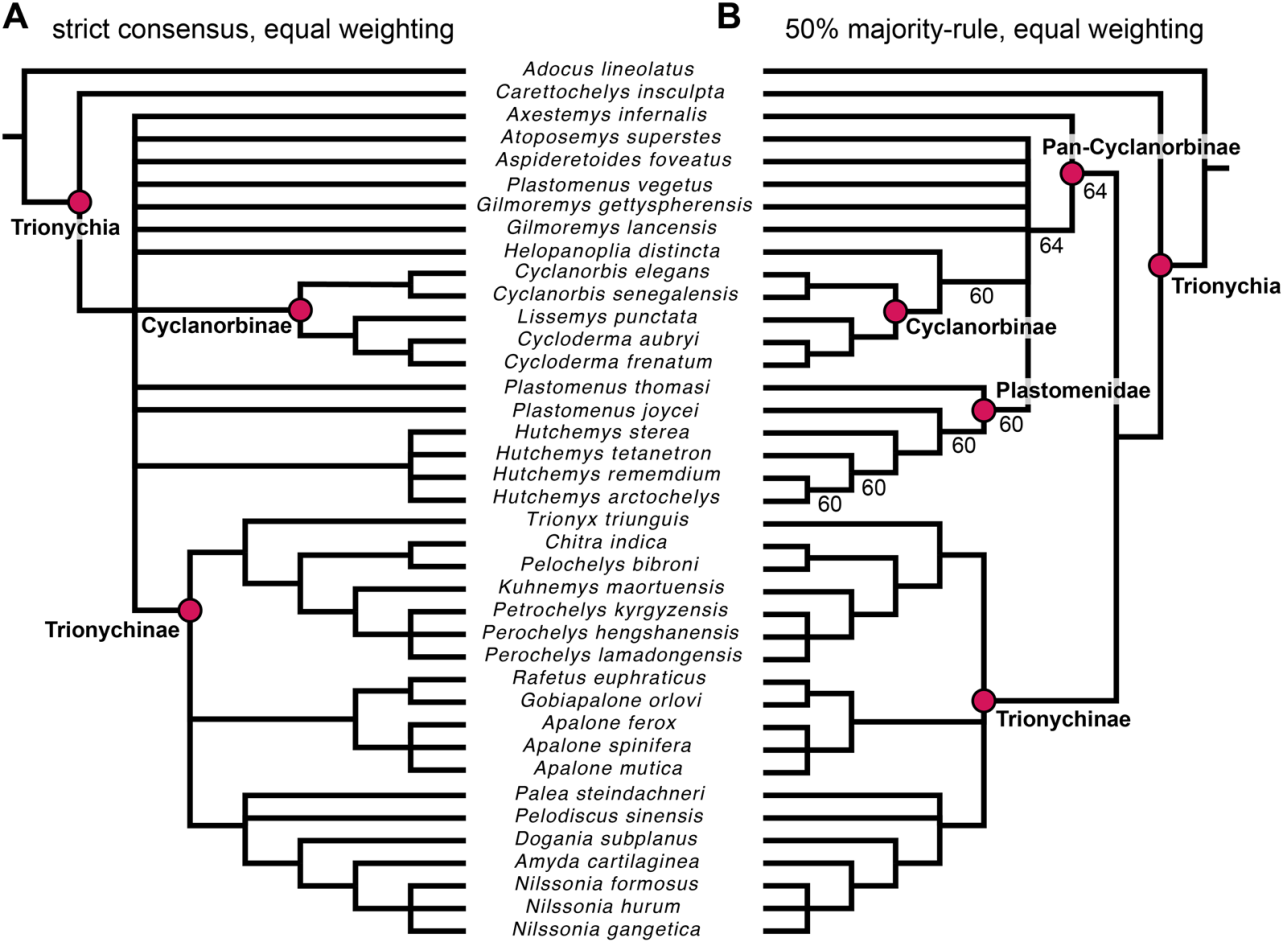
Phylogenetic results of parsimony analysis using equal weights. **A** strict consensus tree of 75 MPTs. **B** 50% majority rule tree constructed from MPTs, with numbers indicating the proportion of MPTs in which a specific node is found. Nodes without numbers in B are found in all MPTs, whereas polytomies represent nodes in which no possible resolution receives more than 50% support. Main clades are indicated

The initial equal weighting analysis retrieved 70 MPTs of 393 character state transitions, and a second round of TBR found 5 additional MPTs. The strict consensus tree of these 75 MPTs is poorly resolved and did not recover a monophyletic *Plastomenidae* (Fig. 12A). Instead, most taxa commonly found as plastomenids (including *Plastomenus* and *Gilmoremys*) were found in a large polytomy with *Helopanoplia distincta*, *Aspideretoides foveatus*, *Atoposemys superstes*, *Axestemys infernalis*, a clade that unites the species of *Hutchemys*, crown cyclanorbines, and crown trionychines. However, this large polytomy is only caused by *Plastomenus vegetus*, which variably jumps between being a stem cyclanorbine (in the majority of MPTs) and a stem trionychine. All other taxa commonly found as plastomenids are recovered as stem cyclanorbines across all MPTs, albeit in different arrangements. Among trionychines, *Gobiapalone orlovi* is found to be the sister taxon to *Rafetus euphraticus* in all MPTs (Fig. 12A). *Perochelys lamadongensis*, *Petrochelys kyrgyzensis*, *Perochelys hengshanensis*, and *Kuhnemys maortuensis* consistently form a clade of Cretaceous trionychines that was retrieved as sister to the clade formed by *Chitra indica* and *Pelochelys bibroni* (Fig. 12A).

In the 50% majority rule consensus tree, the fossil taxa found in the large polytomy are all moved onto the stem lineage of cyclanorbines (Fig. 12B). Specifically, *Helopanoplia distincta* is found as the immediate sister taxon to crown *Cyclanorbinae*. *Plastomenidae* is found as a clade that includes the species of *Hutchemys*, *Plastomenus thomasii*, and *Plastomenus joycei*. However, these two species of *Plastomenus* are paraphyletically arranged with regards to *Hutchemys*, and the genus is polyphyletic overall, as *Plastomenus vegetus* is found outside of *Plastomenidae*, in a polytomy with plastomenids, *Helopanoplia* + *Cyclanorbinae*, *Aspideretoides foveatus*, *Atoposemys superstes*, and the two species of *Gilmoremys*. Outside of this polytomy, *Axestemys infernalis* is found as the sister taxon to all remaining taxa of the cyclanorbine total lineage. MPTs, the strict consensus tree, and the 50% majority tree are deposited as Additional file 5: Data 4.

**Fig. 13:**
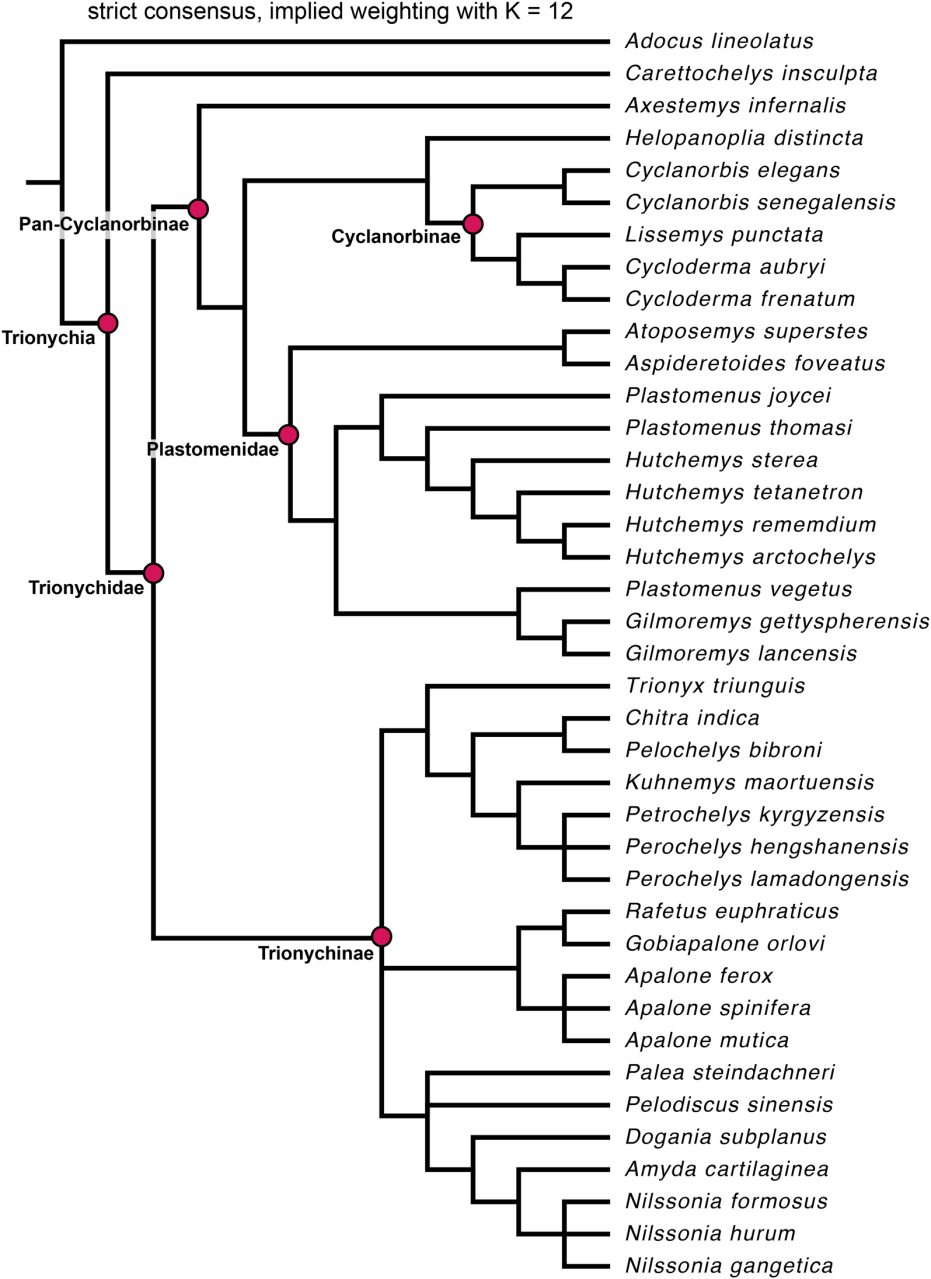
Phylogenetic results from parsimony analysis using implied weights with a concavity constant of K = 12. Important clades are labelled

Implied weighting using a concavity constant of K = 12 recovered 3 MPTs (score of 15.97). The three tree topologies of the implied weighting tree are among those topologies recovered as MPTs in the equal weighting analysis, so that the implied weighting analysis also requires the same tree length of 393 character transformations. The strict consensus of these trees is well resolved (Fig. 13). Its overall topology is similar to that of the 50% majority rule consensus tree using equal weights. The tree includes a large stem lineage of cyclanorbines, which includes *Helopanoplia distincta* as the immediate sister of crown *Cyclanorbinae*, most taxa commonly retrieved as plastomenids as further stem cyclanorbines, and *Axestemys infernalis* as the sister to all other total-cyclanorbines. *Plastomenidae* is found as a large clade in which the genus *Plastomenus* remains polyphyletic, whereas the relationships of *Plastomenus thomasii* and *Plastomenus joycei* are as described for the 50% majority rule tree of the equal weights analysis, and whereby *Plastomenus vegetus* is the sister taxon to a monophyletic genus *Gilmoremys*. *Aspideretoides foveatus* and *Atoposemys superstes* form a sister relationship at the base of *Plastomenidae*. Again as in the equal weights analysis, the implied weighting analysis supports *Gobiapalone orlovi* as the sister of *Rafetus euphraticus*, and members of the *Perochelys*-group as deeply nested among trionychines, indicating that downweighting of homoplasy alone does not move these Cretaceous taxa into more stemward positions. The MPTs and strict consensus tree of the implied weighting analysis are provided as Additional file 6: Data 5.

**Fig. 14:**
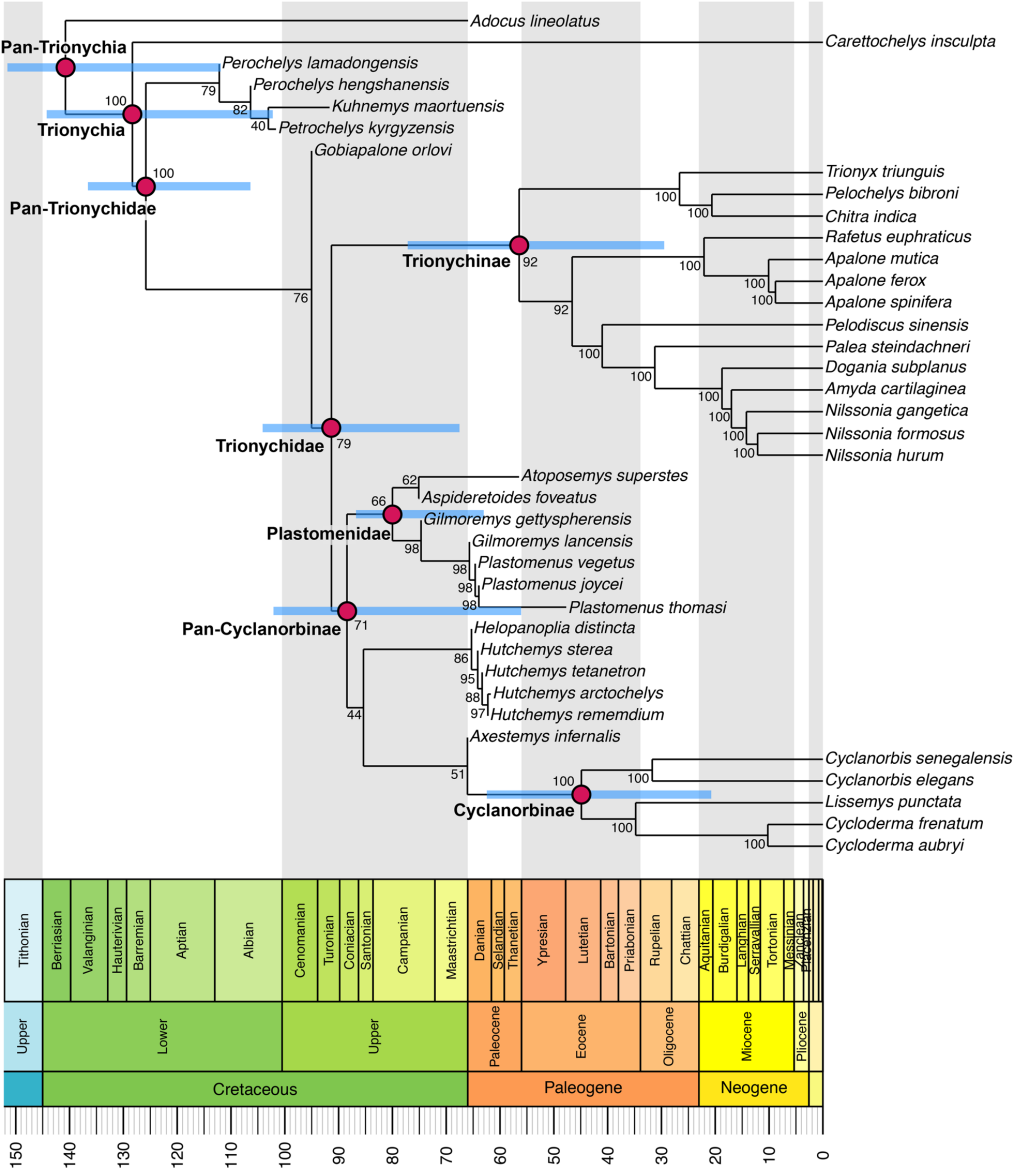
Phylogenetic topology of the MCC tree retrieved from the Bayesian tip-dating analysis, scaled to geologic time. Major clades are labelled and shown with 95% HPD intervals for their estimated node ages. Small numbers at nodes are clade credibility values

The Bayesian tip dating analysis implies a median root age of the posterior tree sample of 140.8 Ma for *Pan-Trionychia* (Fig. 14; Table 1), which is well within the bounds of our root age prior. This Early Cretaceous age for the total group of *Trionychia* as sampled herein also supports an Early Cretaceous divergence time for crown trionychians, at 123.8 Ma. This is younger than the median divergence time estimates of Thomson et al. (2021), but within the confidence intervals of their findings. Our analysis further finds a divergence time for the crown group of *Trionychidae* at 94.1 Ma. This Cenomanian age agrees with that reported by Thomson et al. (2021). The crown clades of *Trionychinae* and *Cyclanorbinae* have divergence times of 50.7 Ma and 36.6 Ma, respectively. The Early Eocene (Ypresian) age retrieved for trionychines is slightly younger than that reported in Thomson et al. (2021), but again lies within the confidence interval of their estimates. Our Late Eocene (Priabonian) age for the divergence of cyclanorbines is slightly older than the Oligocene estimate by Thomson et al. (2021). Whereas Thomson et al. (2021) show relatively narrow 95% highest posterior density (HPD) values for *Cyclanorbinae*, our confidence interval for the age of this node is wide and extends into the Paleocene (60.7 Ma; see Table 1). Overall, our divergence time estimates are consistent with those from Thomson et al. (2021), despite the differences in the datasets (morphological vs. molecular) that underly the clock approach.

**Table 1:** Age estimates and their uncertainty for selected clades

Clade	Age estimates [Ma]			median character transition rate [per Ma]
	lower 95% HPD	Median	higher 95% HPD	
*Pan-Trionychia*	130.00	140.77	169.63	–
*Trionychia*	118.04	133.95	160.04	0.4
*Pan-Trionychidae*	113.0	123.77	143.19	2.74
*Trionychidae*	81.34	94.11	117.91	1.55
*Trionychinae*	30.0	50.69	77.63	0.43
*Pan-Cyclanorbinae*	78.22	91.88	124.24	1.81
*Plastomenidae*	75.93	82.7	99.66	2.61
*Cyclanorbinae*	19.02	36.58	60.71	1.27

A list of all node age estimates and evolutionary rates is provided in Additional file 7: Data 6. HPDs are highest posterior densities

The ‘allcompat’ tree output of MrBayes topologically uses a 50% majority rule consensus tree of the posterior sample of trees, to which all compatible groups are added. Low topological resolution among the ‘allcompat’ tree indicates that there are few groupings (i.e., clades) consistently retrieved across the entire sample of posterior trees (shown in Additional file 7: Data 6). Besides the constrained topologies of extant trionychids, it is noteworthy that this includes the clade *Plastomenidae*, which is composed of the species of *Plastomenus* and *Gilmoremys* only in the ‘allcompat’ tree. *Plastomenidae* is found in a large polytomy with the extant clades and all other fossils as individual taxa (Additional file 7: Data 6). We also computed a maximum clade credibility (MCC) tree (Fig. 14), i.e., the tree with the maximum product of the posterior clade probabilities. Clade credibility values are indicated for all nodes in Fig. 14 and show that only two nodes are associated with credibility values < 50. The MCC tree topology implies 406 character state transitions and is thus 13 steps longer than the MPTs generated by the parsimony analyses. According to a Templeton’s test, this constitutes a significant difference (T-statistic = 135, P = 0.0236, N = 30) in tree length compared to the first of the equal weighting-analysis MPTs listed in Additional file 5: Data 4.

In the MCC tree, cyclanorbines have an extended stem lineage that includes a succession of three clades forming a paraphyletic grade (Fig. 14). Of these, *Axestemys infernalis* is found as the immediate sister taxon to *Cyclanorbinae*. Further stemward is the clade composed of *Hutchemys* spp. and *Helopanoplia distincta*. We find *Plastomenidae* to be the earliest branching clade of stem cyclanorbines. As found in the ‘allcompat’ tree, this includes the species of *Plastomenus* as the sister group to *Gilmoremys* spp., the latter of which are not found to be monophyletic at the genus level (Fig. 14). However, differing from the ‘allcompat’ topology, *Plastomenidae* in the MCC tree additionally includes a clade of *Aspideretoides foveatus* and *Atoposemys superstes* as the sister group to all other plastomenids (Fig. 14). *Trionychidae* does not have fossil stem members in our MCC tree. *Gobiapalone orlovi* is found as the immediate sister to *Trionychidae*, and thus as a stem trionychid (Fig. 14). One node further stemwards, we find a clade of Cretaceous trionychids, which includes *Kuhnemys maortuensis*, *Petrochelys kyrgyzensis*, *Perochelys hengshanensis*, and *Perochelys lamadongensis* (Fig. 14).

As the stem-trionychid positions for taxa of the *Perochelys*-group make stratigraphic sense, and as the MCC fulfills the criterion of being fully resolved, which is required for a sensible character optimization, we used the MCC tree for a full character optimization. Although character optimization lists and the discussions thereof can amount to quite tedious text, we consider this an important part of an article like ours, which presents a large number of new or modified phylogenetic characters along a novel phylogenetic hypothesis that at least partially differs from previous topological arrangements. As outlined in the methods, character discussions must also extend beyond discussing unambiguous synapomorphies for a comprehensive understanding of the character support for various clades. Here, we report only synapomorphies of the clades most relevant to our study, although *Plastomenidae* as the primary focus of this study is discussed in more detail. The full optimization (i.e., all nodes) is provided as Additional file 8: Data 7, and a key to all numbered nodes is provided in the PAUP-log file of the optimization procedure (Additional file 9: Data 8).

The clade *Pan-Trionychidae* is supported by eight unambiguous synapomorphies. This relatively large number is maybe unsurprising, given that our *Pan-Trionychidae* taxonomically includes Cretaceous fossil turtles that have previously often been found deeply nested among crown trionychids, and which must therefore share numerous features with constituents of this clade. However, this list also shows that morphological support for the stem placement of these enigmatic fossils exists, even if this implies a longer tree length than acceptable under a strict parsimony criterion (see above). The synapomorphies for *Pan-Trionychidae* provide evolutionary information in the sense that they imply the following features to be plesiomorphically present during (crown) trionychid evolution: an extremely wide nuchal (width-length ratio > 4); the absence of peripherals; the presence of seven neurals; the presence of suprascapular fontanelles which close during ontogeny; open hyo-, hypo- and xiphiplastral contacts that are retained even in adults; a costal VIII that is square or wider than long; a hyoplastron with a strongly serrated medial margin; and the presence of a well-developed suborbital crest. The ACCTRAN optimizations for this clade are not specifically mentioned (but see Table 2), as these can currently not be verified for the fossil taxa of the clade. The trionychid crown clade is only unambiguously supported by the presence of a xiphiplastral callosity. This small number of unambiguous synapomorphies, in contrast to the much longer list of pan-trionychid synapomorphies, indicates that the principal trionychid bauplan was already established on its stem lineage. Rates of morphological evolution estimated during the Bayesian analysis further indicate that the branch leading to *Pan-Trionychidae* has some of the highest evolutionary rates (rate = 2.74; Table 1), which is nearly twice as high as the rate leading to the trionychid crown (rate = 1.55; Table 1).

**Table 2:** Synapomorphies of selected clade, as optimized on the MCC tree topology

Clade	Unambiguous	ACCTRAN	DELTRAN
*Pan-Trionychidae*	1:4, 5:4, 16:2, 20:2, 75:0, 84:0, 85:0, 98:1	7:2, 31:2, 40:3, 67:1, 102:1, 111:0	NA
*Perochelys*-clade	16:1, 18:0, 20:3, 25:2	29:2, 110:0	7:2
*Trionychidae*	11:1	7:1, 34:2, 35:2, 101:1	88:1
*Trionychinae*	1:3, 59:2, 98:0	31:3, 48:1, 104:1	68:2, 99:1
*Pan-Cyclanorbinae*	4:1, 19:2, 20:1, 77:1, 85:1	29:2, 38:2, 49:1, 63:0, 68:1, 76:1, 79:1, 99:0, 105:0, 106:1	34:2, 35:2, 48:2
*Plastomenidae*	33:2	3:3, 28:2, 50:2, 69:1, 80:1, 83:1, 93:1, 99:2, 100:1, 101:0, 107:1, 110:2, 111:1	76:1
*Gilmoremys* + *Plastomenus*	14:2, 91:1	21:2, 31:3, 37:3, 79:2, 82:1, 94:1, 109:2	NA
*Gilmoremys lancensis* + *Plastomenus* spp.	NA	9:1; 69:0	28:2; 29:2; 31:3; 37:3; 38:2; 50:2; 80:1; 82:1; 93:1; 100:1; 105:0; 109:2; 110:2; 111:1
*Plastomenus* spp.	24:1, 25:2	23:1, 54:2, 97:0, 102:2	NA
*Plastomenus thomasii*	69:1; 72:1; 75:1; 89:1	NA	9:1; 54:2; 83:1; 94:1; 97:0; 99:2; 102:2; 106:1; 107:1
*Helopanoplia* + *Hutchemys*	14:2, 75:1	72:1, 78:1, 84:1, 90:1	15:2, 89:1
*Axestemys* + *Cyclanorbiae*	NA	76:0	31:1, 63:0, 101:1
*Cyclanorbinae*	1:2, 13:2, 66:2, 110:0	23:1, 85:0	15:2, 29:2, 38:2, 39:1, 52:1, 61:2, 92:0, 105:0

Unambiguous optimizations are those for which ACCTRAN and DELTRAN criteria agree. Numbers before the colon correspond to character numbers, and numbers after the colon correspond to character states

*Trionychinae*, one of the principal clades within *Trionychidae*, is supported by three unambiguous synapomorphies: a reduction of the relative nuchal width to a width-length ratio that ranges between 3–4; an enlarged number of ossifications in the cornu branchiale II (2–6); and the loss of a well-developed suborbital crest. As trionychines, as sampled herein, do not have a fossil stem lineage in our analysis, we do not discuss the ambiguous synapomorphies for this clade, but they are listed in Table 2. Trionychines are associated with low rates of evolution (rate = 0.43; Table 1).

The other principal clade of trionychids in our tree is *Pan-Cyclanorbinae*. The clade is well supported by five unambiguous synapomorphies and ten ACCTRAN synapomorphies for which it is currently unclear if they are present in all pan-cyclanorbines or only subsets of more crownwardly placed nodes. The unambiguous synapomorphies are unfused first and second neurals (i.e., presence of a preneural); a point of reversal for the orientation of neurals that is located on neural VI; the absence of suprascapular fontanelles (already closed at hatching); the presence of lateral branches of the entoplastron that loosely abut against the hyoplastron, which has an anterior flap or shoulder; and the absence of a strongly serrated medial margin of the hyoplastron. Ambiguous character optimizations for *Pan-Cyclanorbinae* are listed in Table 2 and in part discussed in more detail for clades nested within the clade. The evolution of *Pan-Cyclanorbinae* (rate = 1.81) as well as crown *Cyclanorbinae* (rate = 1.27) are associated with moderate rates of character evolution that are higher than those seen in trionychines.

Among plastomenids, *Plastomenus thomasii*, the taxon described herein, has four unambiguous autapomorphies (Table 2). According to our analysis, these are all shell characters: a trionychid surface sculptural pattern that grades towards the center of the carapacial and plastral disk to a smooth pattern; a dorsal rim of the costals that splits into separately protruding dorsal and visceral portions; hyo-, hypo- and xiphiplastra that contact one another fully along the mid-line; and the presence of a metaplastic ossification that rolls onto the posterior aspects pf the lateral process of the hypoplastron. In addition, *Plastomenus thomasii* has nine DELTRAN autapomorphies, all of which are alternatively retrieved under ACCTRAN as synapomorphies of various clades within *Plastomenidae* or of *Pan-Cyclanorbinae* (Table 2). Two noteworthy DELTRAN optimizations include the complete reduction of a postorbital, which is either an autapomorphy of *Plastomenus thomasii* or a synapomorphy of the genus, given that the sister species *Gilmoremys lancensis* as well as successive outgroups have a postorbital; and the presence of a massive infolding ridge of the quadrate, which again is absent in *Gilmoremys lancensis*.

The genus *Plastomenus* is found with two unambiguous synapomorphies of the shell (Table 2), namely a short bridge length and a large body size in which adults exceed lengths of 200 mm. The node that unites the species of *Plastomenus* with *Gilmoremys lancensis* has, as expected, a large number of cranial and mandibular DELTRAN optimizations that are optimized within the clade *Plastomenidae* using ACCTRAN (Table 2). They represent shared features of the skull anatomy of *Plastomenus thomasii* and *Gilmoremys lancensis*, which could plausibly evolve in more basal nodes of *Plastomenidae* given that most other plastomenids do not preserve skull material. These characters are the presence of a jugal-squamosal contact; the presence of a jugal-parietal contact on the skull surface; a strongly emarginated dorsal edge of the external naris; a division of the foramen palatinum posterius into two small foramina; the presence of an epipterygoid-prootic contact anterior to trigeminal foramen in the majority of specimens; the presence of an extensive secondary palate formed by the in-folded maxillae; a contribution of the exoccipital to the bar separating the foramen jugulare posterius and fenestra postotica; the presence of foramina in the vomer; the presence of an extremely elongated mandibular symphysis; the presence of a strong expansion of the lingual margin of the dentary, forming a spatulate symphyseal area; the enlarged size of the foramen dentofaciale majus; and the presence of a dorsal surangular foramen. Two further DELTRAN characters of *Gilmoremys lancensis* + *Plastomenus* spp. are optimized as synapomorphies of *Pan-Cyclanorbinae* as a whole under ACCTRAN. These are the enclosure of the foramen jugulare posterius and the absence of a lateral flange on the external pterygoid process.

The next successive node in the MCC tree is that which unites all species of *Gilmoremys* with all species of *Plastomenus*. This is an interesting node as this OTU combination is that commonly retrieved in previous phylogenetic studies (e.g., Lyson et al., 2021). The *Gilmoremys* + *Plastomenus* node includes an extended midline contact of xiphiplastral callosities along much of the midline, and the presence of carapacial striations in adults as unambiguous synapomorphies (Table 2). The ACCTRAN optimizations additionally include the presence of an I-shaped epiplastron; the presence of two lateral hypoplastral processes; and a number of cranial and mandibular characters already listed as DELTRAN characters of the node (*Gilmoremys lancensis*, *Plastomenus* spp.).

The taxonomic clade content of *Plastomenidae* of the MCC tree is identical to the topology retrieved in the 50% majority rule consensus tree of the equal weighting analysis. This clade is unsurprisingly supported by only few unambiguous characters, but many ACCTRAN optimizations. The only unambiguous synapomorphy of plastomenids is a basioccipital-palatine contact, which is overall rare among trionychians, and only appears in our sample within the plastomenids *Aspideretoides foveatus*, *Gilmoremys lancensis*, *Plastomenus thomasii*, and homoplastically in the stem-cyclanorbine *Axestemys infernalis*, the trionychine *Rafetus euphraticus*, and *Carettochelys insculpta*. ACCTRAN optimizations for plastomenids again include many cranial and mandibular features discussed as DELTRAN optimizations of *Gilmoremys* + *Plastomenus*, but additionally include an alignment of the anterior end of the first thoracic vertebra with the anterior edge of the nuchal; a parietal contribution to the orbits; a supraalveolar foramen that is present as a fenestra-like opening into the supraalveolar canal; the absence of a tongue groove; and a prootic contribution to the formation of the lateral semicircular canal. Plastomenids show high rates of evolution (rate = 2.61; Table 1) according to our Bayesian analysis.

*Helopanoplia distincta* and the species of *Hutchemys* are not found to be plastomenids in our analyses, and instead form an independent clade of stem cyclanorbines in the MCC tree. This clade is supported by two unambiguous plastral synapomorphies. The first is the presence of an extended midline contact of the xiphiplastral callosities along much of the midline. The second one is that the hyo-, hypo-, and xiphiplastra contact one another fully along the entire midline in adults, which is autapomorphically shared by *Plastomenus thomasii* (see above). In addition, the ACCTRAN optimizations are likely or at least potential synapomorphies of the clade, and include a dorsal rim of the costals which splits into separately protruding dorsal and visceral portions, a lateral bridge ossification that extends laterally beyond the hyo- and hypoplastral bridge processes, a costal VIII that is significantly taller than wide, and an absence of metaplastic bone growth over costal rib ends VII and VIII. The *Hutchemys* + *Helopanoplia* clade shares with more crownwardly positioned pan-cyclanorbines a long bridge (unambiguous synapomorphy, homoplastically present in *Plastomenus thomasii*), as well as a range of ACCTRAN synapomorphies. The latter index characteristics that are present in the crownward part of the cyclanorbine stem lineage or the cyclanorbine crown itself, and which could plausibly already be present in this node but which are absent in plastomenids (unless homoplastically gained). Specifically, these are the presence of epiplastral callosities; the presence of an entoplastral callosity (homoplastically present in *Gilmoremys lancensis* + *Plastomenus* spp.); a hypoplastral-xiphiplastral contact in which the hypoplastron extends laterally to the xiphiplastron; the presence of metaplastic ossifications of the hypoplastron that rolls onto the posterior aspect of the lateral hypoplastral process (variably present among plastomenids); an absence of a dorsolateral emargination of the external naris; the presence of a pterygoid contribution to the bar separating the foramen jugulare posterius and the fenestra postotica; the absence of an opisthotic contribution to the bar separating the foramen jugulare posterius and the fenestra postotica; the frequent presence of an epipterygoid-palatine contact; the frequent contact of the epipterygoid and prootic anterior to the trigeminal foramen; the ontogenetically early fusion of the epipterygoid; and the presence of a close contact between the basihyals, which project anteriorly.

The next successively more crownward node is that of *Axestemys infernalis* + crown cyclanorbines. This node lacks unambiguous synapomorphies. For this node, the DELTRAN optimizations are interesting, because they index characters present in crown cyclanorbines and *Axestemys infernalis* for which it is unknown if successive outgroups already possess them. Thus, these characters are those that currently support the unusual position of *Axestemys infernalis* as a stem-cyclanorbine. These DELTRAN optimizations are the absence of a dorsolateral emargination of the external naris; the absence of a posterior intermandibular foramen; and the presence of a tongue groove. The latter probably provides only weak morphological support, as tongue grooves are also widely present among trionychines. The other two characters are also not unique to cyclanorbines among extant taxa, but rare among trionychines.

Crown cyclanorbines are supported by four unambiguous synapomorphies: the nuchal is more than twice as wide as it is anteroposteriorly long; the hyo- and hypoplastra fuse just after hatching; a (distinct) metischial process is absent; the foramen dentofaciale majus is completely reduced. In addition, there are a number of DELTRAN characters (Table 2), which have largely already been discussed as potential synapomorphies of deeper nested nodes along the cyclanorbine stem, as well as ACCTRAN synapomorphies that reflect upon variation among living cyclanorbines, which we will not comment on further.

## Discussion

### Infraorbital arterial pattern

Albrecht (1967) described the infraorbital arterial pattern for *Apalone spinifera*, summarized here as follows: the infraorbital artery is one of three main subbranches of the pseudopalatine artery. Coming from the cerebral artery, the pseudopalatine artery extends anteriorly from the basicranium along the medial surface of the secondary braincase wall. At the level of the interorbital foramen, the infraorbital artery branches laterally into the orbital fossa, where it gives off two posterior branches, the pseudomandibular artery (without osteological correlate), and the inframaxillary artery, which extends through the intrapalatine canal and exits at the ventral skull surface through the foramen palatinum posterius. More anteriorly, the supramaxillary artery branches off the infraorbital artery and enters its namesake foramen. Even more anteriorly, within the foramen orbito-nasale, the infraorbital finally gives rise to two branches, the posterior nasal artery (no osteological correlate), and the superior alveolar artery, which enters the maxilla through the supraalveolar foramen. Although our data does not allow us to investigate the arterial patterns directly, our osteological sample of trionychids indicates that the same infraorbital pattern is seen across all trionychines: our specimens of *Pelodiscus sinensis*, *Amyda cartilaginea*, *Chitra chitra* and *Apalone spinifera* all show the intrapalatine canals as a short throughlet from the base of the braincase wall to the foramen palatinum posterius and the presence of a foramen supramaxillare and supraalveolar foramen.

Our sample of cyclanorbines (*Lissemys punctata*, *Cyclanorbis senegalensis*) show a slightly different foraminal arrangement. Although the intrapalatine canal is present, as in trionychines, the foramen supramaxillare is the only entry into the supraalveolar region of the maxilla, and a supraalveolar foramen is absent in the medial wall of the nasal fossa. It seems possible that the suborbital crest observed in cyclanorbines, but not trionychines, is related to this: the crest effectively borders the orbital fossa from the narial passage, but the infraorbital artery of trionychines is expected to traverse the border between these spaces below the eyeball, which it can only do if there is no bony wall shielding the passage. In this regard, the presence of a suborbital crest in AMNH FR 6015 points to a cyclanorbine-like arterial passage in *Plastomenus thomasii*. Additional evidence comes from the morphology of the supramaxillary canal formed by the foramen supramaxillare, which is indistinct from a supraalveolar canal in AMNH FR 6015, despite the fenestra-like opening of the canal within the nasal fossa. Following this interpretation, the fenestra-like opening (labelled as supraalveolar fenestra herein) is not homologous to the supraalveolar foramen of trionychines.

### Foramina praepalatina in plastomenids?

Lyson and Joyce (2011) identified unusual foramina in the vomer of *Gilmoremys lancensis*, and we extend this observation to *Plastomenus thomasii* herein. Given that similar foramina are absent in extant trionychids for which the content could be tested (e.g., *Amyda cartilaginea*; *Apalone spinifera*; *Chitra chitra*; *Cycloderma frenatum*; *Lissemys punctata*; *Pelodiscus sinensis*), we chose the neutral name “vomer foramina” in our description. Joyce and Lyson (2011) identified these foramina as pertaining to the pseudopalatine artery. This hypothesis is based on comparisons with the dissection of Albrecht (1967), who described the pseudopalatine artery as traversing the narial passage above the vomer while giving off numerous small arteries towards the palate, which, however, are not further discussed in the text. As an alternative, we here raise the possibility that these openings are the equivalent to the foramina praepalatina of other turtles (Gaffney, 1972), which transmit the anterior nasal artery (Albrecht, 1967), but which are generally absent in trionychids. In AMNH FR 6015 and *Gilmoremys lancensis*, the vomer foramina are limited entirely to the vomer, and positioned so that they connect the palate with the nasal passage, instead of entering the nasale capsule directly. Although foramina praepalatina in non-trionychid turtles are often positioned completely within the premaxilla (e.g., *Sternotherus minor*, FMNH 211696: Evers & Benson, 2019), the position of the foramina actually varies among turtles: they may be positioned between the premaxillae and vomer (e.g., *Adocus lineolatus*: Meylan & Gaffney, 1989), or even lie completely within the vomer, although at the very front of the bone (e.g., Meylan & Gaffney, 1989). Thus, the osteological position of the vomer foramina may be compatible with their identification as foramina praepalatina. If the vomer foramina of plastomenids indeed were homologous to the foramina praepalatina of other turtles that retain these, this would have implications for the homology of the intermaxillary foramen of trionychids: this opening in the anterior palatal region of trionychids is often homologized with the foramina praepalatina in phylogenetic characters of turtles (e.g. Joyce, 2007: ch. 24). This homology must be rejected if an identification of the vomer foramina as foramina praepalatina was correct, as the plastomenid *Gilmoremys lancensis* has both the foramina praepalatina and the foramen intermaxillaris (Joyce & Lyson, 2011; Joyce et al., 2016). This would support studies that argued these palatal openings should not be considered homologous and treated as separate phylogenetic characters (Anquetin, 2012; Evers & Benson, 2019). As we find plastomenids to be nested among crown-group trionychids, it currently seems likely that the unusual vomer foramina are a novel anatomical structure, rather than plesiomorphically related foramina praepalatina. We expect dissections focused on the exact path of the arteries in the anterior skull region to perhaps provide meaningful data that might further elucidate this issue.

### Phylogeny and evolution of plastomenids among trionychids

There is strong support based on our data, parsimony and Bayesian analyses that cyclanorbines have an extended stem lineage composed of taxa that are commonly interpreted as plastomenids (e.g., Lyson et al., 2021), alongside *Axestemys infernalis*. This is despite a large polytomy in the strict consensus tree of the equal weighting analysis, which suggests that there is character conflict for the placement of plastomenids among trionychids: our results show that this is caused by a single taxon, *Plastomenus vegetus*. Plastomenids partially fill the stratigraphic gap which results from shallow divergence times of crown cyclanorbines during the Priabonian (late Eocene), the earlier origin of trionychines during the Ypresian (early Eocene), and an estimated divergence of both clades at the crown node of *Trionychidae* during the Cenomanian (early Late Cretaceous), which are implied both by molecular divergence dates (e.g., Thomson et al., 2021) as well as ours.

The exact topological arrangement of stem cyclanorbines varies among different phylogenetic analyses presented herein, as does the taxonomic content of *Plastomenidae*, which is determined by the relative relationships of fossil turtles with *Plastomenus thomasii* and crown cyclanorbines. According to parsimony analysis, *Plastomenidae* minimally includes *Plastomenus thomasii*, *Plastomenus joycei*, and the species of *Hutchemys*, which form a monophylum (50% majority rule tree of equal weighting analysis). The clade may additionally include *Plastomenus vegetus*, the species of *Gilmoremys*, *Atoposemys superstes*, and *Aspideretoides foveatus* (but not *Helopanoplia distincta*; implied weighting). The Bayesian results differ by removing the *Hutchemys*-group (which also includes *Helopanoplia distincta*) from *Plastomenidae*, and instead finding them as a clade of non-plastomenid stem cyclanorbines. As a result, the species of *Gilmoremys* become the closest relatives of *Plastomenus* spp., a result that was also found in previous analyses of the group (e.g., Lyson et al., 2021). *Atoposemys superstes* and *Aspideretoides foveatus* form a clade of basal plastomenids in both the implied weighting and Bayesian analyses, but there is no support for or against this specific arrangement according to equal weighting. Whilst the Bayesian analysis recovers a monophyletic genus *Plastomenus*, the implied weighting topology and 50% majority rule consensus tree of the equal weighting analysis both recover a polyphyletic genus *Plastomenus* (with *Plastomenus vegetus* being only distantly related to the other two species), that is furthermore paraphyletic with regard to the genus *Hutchemys*. It is noteworthy that a monophyletic *Plastomenidae* including the species of *Plastomenus* and *Gilmoremys* recovers strong support from the Bayesian analysis, as this clade is even found in the ‘allcompat’ tree of posterior samples and has a clade credibility support of 98 (Fig. 14). Although the details of these differences may be complex, it is clear that different methodologies all find support for a stem cyclanorbine identity of plastomenids and closely related fossil taxa. In addition to plastomenids, *Axestemys infernalis* is found as a stem cyclanorbine across all analyses. Although this species was not included in phylogenetic analyses in its initial description (Joyce et al., 2019), other species of the genus have been found as closely related to the trionychines *Rafetus* spp. and *Apalone* spp. in analyses that also include cyclanorbines and plastomenids (Vitek, 2012; Danilov et al., 2014). Although the position of *Axestemys infernalis* is not the focus of this study, we think its novel position may at least in part result from increased character sampling, as we examined the skull anatomy for this taxon based on µCT data.

Our analyses re-iterate that plastomenids are characterized by a high number of unusual features, although incomplete fossils hinder the unambiguous optimization of many of these characters onto the node uniting all plastomenids vs. nodes within the clade. Specifically, *Plastomenus thomasii* shows a high number of similarities in the cranium and mandible to *Gilmoremys lancensis*, but it is possible that these characterize plastomenids more widely. The continued description of new fossils will provide the test for this question.

### Congruence of Bayesian results with stratigraphy and molecular evolution

The stratigraphic fit of the topologies recovered across our analyses is highest in the Bayesian analysis. This is expected under tip-dating methods, as the fossilized birth–death tree model assigns higher likelihoods to trees with better stratigraphic fit (King, 2021). Notwithstanding, the implicit expectation of stratigraphic fit in Bayesian tip-dating methods can be overturned by strong character evidence (King, 2021). Both phenomena are observed in our analysis. For instance, the Bayesian methodology has a strong impact on the relatively old fossils from the Cretaceous that belong to the *Perochelys*-group, which have been variously interpreted as stem trionychids or deeply nested crown trionychids (e.g.; Brinkman et al., 2017; Georgalis & Joyce, 2017; Li et al., 2015). The consideration of stratigraphic position during the estimation of topology clearly favors a stem-trionychid interpretation of these taxa, as well as for the Cretaceous *Gobiapalone orlovi*. The research by King (2021) suggests that the alternative position of the *Perochelys*-group as highly nested within *Trionychinae* that is supported by our parsimony analyses (see also: Li et al., 2015; Brinkman et al., 2017) may be based on weak character evidence—a hypothesis supported by parsimony studies that find many different most parsimonious solutions for the position of this group of turtles (Li et al., 2015). It should be noted that parsimony results that place the *Perochelys*-group (or individual taxa thereof; Li et al., 2015), as the sister to extant trionychid genera have basically no independent support from molecular divergence time estimates, as even the stratigraphically deepest diverging genera retrieve upper age estimates no older than from the early Eocene (Thomson et al., 2021). As such, trionychids as a group with highly similar morphological taxa or sub-clades of vastly different ages may be a case in which homoplasy can only be detected by using tests that do not only rely on morphological data, as has been documented for some non-turtle groups (see, for instance, Lee & Yates, 2018: gharials). This is especially important as even implied weighting, as a strategy to downweigh homoplasy, fails to produce results that are congruent with stratigraphy or molecularly inferred node ages, at least among our dataset (see Brinkman et al., 2017 for a study in which implied weighting does result in stemward slippage of the *Perochelys*-group). Our Bayesian analysis implies a tree length that is significantly higher than most parsimonious solutions when compared statistically (Templeton’s test: *T*-statistic = 135, *P* = 0.0236), which indicates that it is unlikely to find this particular topology based on cladistic analysis alone (i.e., excluding stratigraphic data). Thus, fossilized-birth death models in tip-dated Bayesian analyses are a promising method alongside parsimony to explore trionychid evolution, and as closer stratigraphic fits and compatibility with molecular data are clearly desirable properties of morphology-based phylogenies. These results also affect other aspects of evolution, such as biogeography. For example, as the oldest pan-trionychids are all Asian, our Bayesian phylogeny implies an Asian origin of the group (Georgalis & Joyce, 2017; Jasinski et al., 2022). The direct integration of morphology, stratigraphy, and molecular sequence data in total-evidence approaches is the logical next step forward.

Although the interpretation of plastomenids and closely related taxa as stem cyclanorbines is not affected by the choice of phylogenetic method, the in-group relationships of *Plastomenidae* according to Bayesian results still roughly follow the stratigraphic appearance of fossils. This may primarily be due to the incompleteness of many plastomenid fossils, causing the methods to arrange these taxa according to stratigraphic occurrence (e.g., King, 2021). Although the chosen methodology seems to heavily rely on stratigraphic information for reconstructing plastomenid phylogeny, sister-group relationships that break this pattern are apparent, too (e.g., *Atoposemys superstes* + *Aspideretoides foveatus*). This could indicate comparatively strong morphological support for this particular result (e.g., King, 2021), and also illustrates that not all fossil taxa are arranged in stratigraphic sequence by our analysis.

### High early rates of character evolution establish the trionychid body plan

The divergence times we estimated for various clades using a morphological clock and fossil tip-dating match previous dates from calibrated molecular phylogenies (e.g., Thomson et al., 2021). As both methods use different underlying datasets, these divergence time estimates may be seen as independently supported. Although our matrix is not designed to comprehensively sample trionychid evolution more widely, our analyses have some implications for the evolution of the group as a whole. The ages recovered for the divergences of the trionychid and carettochelyid lineages and the origin of crown trionychids (134.0 Ma and 94.1 Ma, respectively) imply a duration of roughly 40 Ma during which the characteristic bauplan of trionychids evolved. However, our character optimization suggests that many typical trionychid characters already evolved early on the trionychid stem lineage. This is evidenced by our extended *Pan-Trionychidae* (including the *Perochelys*-group, *Gobiapalone orlovi*, and crown *Trionychidae*) being supported by a high number of synapomorphies, whereas the node of crown trionychids sees only the addition of few characters, indicating there was only little additional morphological innovation at the origin of the crown clade. This reduces the evolutionary time to accumulate the morphological changes that characterizes the softshell turtle bauplan to less than 11 Ma (the time difference between the origin of *Trionychia* and *Pan-Trionychidae*; Table 1). Given the relatively short branch lengths of the deepest trionychid branches and high morphological distinctness of softshell turtles compared to other turtle groups, early trionychid evolution may be expected to have experienced high rates of morphological evolution. Our Bayesian analysis returns rate estimates that provide a test for this question. Indeed, the branch leading to pan-trionychids is associated with relatively high rates of morphological evolution at 2.74 inferred character transitions per Ma. As a comparison, the average of the median rates associated with each of the extant OTUs is 0.76, indicating the evolutionary rate at the beginning of trionychid evolution is more than 3.5 times as high than the rates at which extant taxa diverge from one another. The evolutionary rate associated with the crown clade of *Trionychidae* still has moderate rates of 1.55 character transitions per million year. This decrease in rate of morphological evolution early after the origination of pan-trionychids is consistent with the notion that the principal morphological innovations of softshell turtles were already established during the evolution of their stem lineage, although higher rates than observed for modern taxa are still invoked during the origination of the crown node of *Trionychidae*. These hypotheses of rate evolution should receive further testing by Bayesian phylogenetic analysis with increased fossil taxonomic sampling.

## Conclusions

Our study adds to the many examples showing that digital models segmented from µCT data can greatly improve our anatomical understanding of fossils. For *Plastomenus thomasii*, our data on AMNH FARB 6015 reveal many unusual skull features that are likely widespread among plastomenids, based on comparisons with *Gilmoremys*. Our phylogenetic results lend support for the hypothesis that plastomenids are stem-cyclanorbines. Bayesian phylogenetics that include stratigraphic data lead to topologies that have stronger stratigraphic congruence than morphology-only, parsimony analyses. The recovered divergence time estimates match those retrieved from molecular studies, and further suggest that the trionychid body plan with its many peculiarities evolved on the trionychid stem-lineage during a relatively short time span of maybe as little as 11 million years and at high rates of morphological evolution. Once this body plan was established, total-group trionychids show comparatively little variation, leading to difficulties in the reconstruction of trionychid phylogeny, and suggesting that the rate of their morphological evolution has varied between different phases of trionychid evolution.

## Supplementary Information

**Additional file 1:** Character List.

**Additional file 2.** Character-taxon matrix.

**Additional file 3.** TNT file with molecular backbone topology and ordered characters.

**Additional file 4.** MrBayes command script.

**Additional file 5.** Multiple tree file containing MPTs, strict consensus tree and 50% majority rule consensus tree of equally weighted parsimony analysis.

**Additional file 6.** Multiple tree file containing MPTs, strict consensus tree and 50% majority rule consensus tree of implied weighted parsimony analysis.

**Additional file 7.** Log file of Bayesian analysis, including convergence parameters, allcompat tree topology, and summary statistics for node and branch parameters.

**Additional file 8.** Spreadsheet with all character optimizations.

**Additional file 9.** Log file of PAUP* optimization, with numerical key for nodes that match those of the optimization spreadseheet.

## Data Availability

The fossil AMNH FARB 6015 is accessioned in the American Museum of Natural History Fossil Reptile collection, and can be assessed pending curatorial permission. The µCT data generated for this study as well as the 3D models derived from segmenting these data are provided at the online repository MorphoSource (Evers & Chapelle, 2022), and can be accessed here: https://www.morphosource.org/projects/000451076. The phylogenetic data matrix is available as a supplementary file to this article.
