# Supplementary material for: Cranial and mandibular anatomy of Plastomenus thomasii and a new time-tree of trionychid evolution
Source: Swiss J Palaeontol. 2023 Mar 16;142(1):1. doi: 10.1186/s13358-023-00267-5 (PMC10020266; doi:10.1186/s13358-023-00267-5)
Supplement: Supplementary file 1 — Additional file 1: Character List. [file 13358_2023_267_MOESM1_ESM.docx]

**Supplementary Text: Character List**

We used the phylogenetic matrix of Lyson et al. (2021; 95 characters, 39 taxa) as a base line matrix for our work. We added 26 new phylogenetic characters. Of these, 18 are cranial characters and 8 are mandibular characters. We deleted four character that were in the original character matrix (characters 38, 56, 84, 88 of Lyson et al. 2021), and slightly modified the state definitions of three additional characters. Remarks section in our character list are given to justify changes and deletions. The new total number of characters is thus 116. Due to the deletion of characters, our character numbers differ from thise of Lyson et al. (2021), but the original character numbers are given in our character list.

**Modified character list**

**Character 1 [same character number as in Lyson et al. 2021]**: Width-length of nuchal bone. 1 = less than 2; 2 = greater than 2; 3 = greater than 3; 4 = greater than 4.

**Character 2 [same character number as in Lyson et al. 2021]**: Anterior and posterior costiform processes of nuchal bone united. 1 = no; 2 = yes.

**Character 3[ same character number as in Lyson et al. 2021]**: Position of anterior edge of first body vertebra relative to nuchal bone. 1 = posterior edge of nuchal; 2 = middle of nuchal; 3 = anterior edge of nuchal.

**Character 4 [same character number as in Lyson et al. 2021]**: First and second neurals fused. 1 = no; 2 = yes.

**Character 5 [same character number as in Lyson et al. 2021]**: Total number of peripherals. 1 = 22; 2 = 20; 3 = 14-18; 4 = 0.

**Character 6 [same character number as in Lyson et al. 2021]**: Prenuchal bone. 1 = absent; 2 = present.

**Character 7 [same character number as in Lyson et al. 2021]**: Size of eighth pleurals. 1 = large; 2 = reduced or absent.

**Character 8 [same character number as in Lyson et al. 2021]**: Epiplastral callosity. 0 = absent; 1 = present.

**Character 9 [same character number as in Lyson et al. 2021]**: Entoplastral callosity. 0 = absent; 1 = present.

**Character 10 [same character number as in Lyson et al. 2021]**: Hyo/Hypoplastral callosity. 0 = absent; 1 = present.

**Character 11 [same character number as in Lyson et al. 2021**]: Xiphiplastral callosity. 0 = absent; 1 = present.

**Character 12 [same character number as in Lyson et al. 2021]**: Supernumerary callosities. 0 = absent; 1 = present.

**Character 13 [same character number as in Lyson et al. 2021]**: Hyoplastra and hypoplastra fuse just after hatching. 1 = no; 2 = yes.

**Character 14 [same character number as in Lyson et al. 2021]**: Extended midline contact of xiphiplastral callosities. 1 = absent; 2 = present along much of the midline.

**Character 15 [same character number as in Lyson et al. 2021]**: Hypo-xiphiplastral union. 1 = xiphiplastra lateral to hypoplastra; 2 = hypoplastra lateral to xiphiplastra.

**Character 16 [same character number as in Lyson et al. 2021]**: Number of neurals. 1 = eight ; 2 = seven ; 3 = six or less.

**Character 17 [same character number as in Lyson et al. 2021]**: Variability in position of neural reversal. 1 = always at same neural; 2 = always at adjacent neurals; 3 = highly variable.

**Character 18 [same character number as in Lyson et al. 2021]**: Pleurals which meet at midline. 0 = none; 1 = eighth only2 = seventh and eighth or eighth only3 = sixth, seventh, and eighth or seventh and eighth3 = sixth, seventh, and eighth or seventh and eighth.

**Character 19 [same character number as in Lyson et al. 2021]**: Point of reversal of orientation of neurals. 1 = seven ; 2 = six; 3 = five; 4 = four.

**Character 20 [same character number as in Lyson et al. 2021]**: Suprascapular fontanelles. 1 = closed at hatching; 2 = closed in large adults only; 3 = open throughout life.

**Character 21 [same character number as in Lyson et al. 2021]**: Epiplastron shape. 1 = J-shaped; 2 = I-shaped.

**Character 22 [same character number as in Lyson et al. 2021]**: Length epiplastra anterior to entoplastron contact. 1 = short; 2 = intermediate; 3 = long.

**Character 23 [same character number as in Lyson et al. 2021]**: Depressions on eighth pleurals for contact of ilia. 1 = present; 2 = absent.

**Character 24 [same character number as in Lyson et al. 2021]**: Bridge length. 1 = long; 2 = short.

**Character 25 [same character number as in Lyson et al. 2021]**: Largest adult size 200 mm or less (disc length). 1 = no; 2 = yes.

**Character 26 [same character number as in Lyson et al. 2021]**: Carapace margin straight to concave posteriolaterally. 1 = no; 2 = yes.

**Character 27 [same character number as in Lyson et al. 2021]**: Sexual dimorphism in disc length. 1 = no; 2 = yes.

**Character 28 [same character number as in Lyson et al. 2021]**: Jugal contacts squamosal. 1 = no; 2 = in one half of sample.

**Character 29 [same character number as in Lyson et al. 2021]**: Jugal contacts parietal on skull surface. 1 = no; 2 = in one half of sample; 3 = yes.

**Character 30 [same character number as in Lyson et al. 2021]**: Vomer contacts prefrontal. 1 = yes; 2 = no.

**Character 31 [same character number as in Lyson et al. 2021]**: Dorsal edge of aperture narium externum laterally emarginated. 1 = no; 2 = weakly; 3 = strongly.

**Character 32 [same character number as in Lyson et al. 2021]**: Dorsal edge of aperture narium externum medially emarginated. 1 = no; 2 = yes.

**Character 33 [same character number as in Lyson et al. 2021]**: Basisphenoid contacts palatines. 1 = no; 2 = yes.

**Character 34 [same character number as in Lyson et al. 2021]**: Vomer divides maxillae. 1 = yes; 2 = no.

**Character 35 [same character number as in Lyson et al. 2021]**: Vomer reaches intermaxillary foramen. 1 = yes; 2 = no.

**Character 36 [same character number as in Lyson et al. 2021]**: Vomer contacts basisphenoid. 1 = no; 2 = occasionally.

**Character 37 [same character number as in Lyson et al. 2021]**: Size of foramen palatinum posterius. 1 = large; 2 = small; 3 = small and divided; 4 = many small openings.

**Character 38 [character 39 in Lyson et al. 2021]**: Foramen jugulare posterius. 1 = open; 2 = enclosed.

**Character 39 [modified from character 40 in Lyson et al. 2021]**: Contribution of pterygoid to bar separating foramen julare posterius. 0 = absent; 1 = present.

*Remarks* : The previous version of this character only considered variation about whether the separating bar was either formed by the pterygoid, or by the opisthotic. However, the exoccipital can also be involved in the bony bar, and combinations of these three bones are variable. Thus, we modified this character to only record the absence vs. presence of a pterygoid contribution to the bar, and encode same type of variation for the exoccipital and opisthotic in separate characters. The original character was thus effectively split into three distinct characters.

**Character 40 [character 41 in Lyson et al. 2021]**: Foramen posterius canalis carotici interni relative to lateral crest of basioccipital tubercle. 1 = above ; 2 = in it; 3 = below.

**Character 41 [character 42 in Lyson et al. 2021]**: Maxilla contacts frontal in front of orbit. 1 = no; 2 = yes.

**Character 42 [character 43 in Lyson et al. 2021]**: Exoccipital contacts pterygoid. 1 = no; 2 = yes.

**Character 43 [character 44 in Lyson et al. 2021]**: Basisphenoid shape. 1 = not medially constricted; 2 = medially constricted.

*Remarks*: We updated the character state names to reflect the scoring of the character, therefore removing the polymorphic state.

**Character 44 [character 45 in Lyson et al. 2021]**: Premaxilla absent. 1 = no; 2 = occasionally; 3 = usually.

**Character 45 [character 46 in Lyson et al. 2021]**: Vomer lost. 1 = no; 2 = yes.

**Character 46 [character 47 in Lyson et al. 2021]**: Jugal contacts orbit. 1 = yes; 2 = no.

**Character 47 [character 48 in Lyson et al. 2021]**: Epipterygoid, if present, contacts the palatine. 1 = yes; 2 = in ca. 50%; 3 = no.

**Character 48 [character 49 in Lyson et al. 2021]**: Contact between pterygoid and foramen nervi trigemini occurs when epipterygoid is present. 1 = yes; 2 = no.

**Character 49 [character 50 in Lyson et al. 2021]**: When epipterygoid is present pterygoid contacts foramen nervi trigemini. 0 = between epipterygoid and quadrate or not at all; 1 = between prootic and epipterygoid or not at all; 2 = between epipterygoid and parietalor not at all.

**Character 50 [character 51 in Lyson et al. 2021]**: Epipterygoid contacts prootic anterior to foramen nervi trigemini. 1 = no; 2 = in ca. 50%; 3 = yes.

**Character 51 [character 52 in Lyson et al. 2021]**: Epipterygoid contacts prootic posterior to foramen nervi trigemini. 1 = no; 2 = yes.

**Character 52 [character 53 in Lyson et al. 2021]**: Epipterygoid fuses to pterygoid. 1 = in subadults; 2 = in adults only; 3 = never.

**Character 53 [character 54 in Lyson et al. 2021]**: Average ratio of intermaxillary foramen length to length primary palate. 0 = 0.07; 1 = 0.20 to 0.402 = about 0.60.

**Character 54 [character 55 in Lyson et al. 2021]**: Postorbital bar relative to orbit. 0 = about 2 times orbit diameter; 1 = about equal to orbit to 1/3 of orbit2 = less than 1/5 of orbit.

**Character 55 [character 56 in Lyson et al. 2021]**: Proportion of processus trochlearis oticum made up by parietal. 1 = 15.6% or less; 2 = 22.1% or more.

**Character 56 [character 58 in Lyson et al. 2021]**: Ventral keel on 8th cervical present and limited to posterior end. 1 = no; 2 = yes.

**Character 57 [character 59 in Lyson et al. 2021]**: Strong dorsal processes on cervicals. 1 = no; 2 = yes.

**Character 58 [character 60 in Lyson et al. 2021]**: Number of ossifications in corpus hyoidis. 1 = 6; 2 = 8.

**Character 59 [character 61 in Lyson et al. 2021]**: Number of ossifications in comu branchiale II. 1 = 1 only; 2 = 44714; 3 = 7 or more.

**Character 60 [character 62 in Lyson et al. 2021]**: Ossifications of comu branchiale II broad and strongly sutured. 1 = no; 2 = yes.

**Character 61 [character 63 in Lyson et al. 2021]**: Basihyals in close contact and projecting anteriorly. 1 = no; 2 = yes.

**Character 62 [character 64 in Lyson et al. 2021]**: Symphyseal ridge strong and present in a depression. 1 = no; 2 = yes.

**Character 63 [modified from character 65 in Lyson et al. 2021]**: Foramen intermandibularis caudalis. 0 = absent; 1 = present at least in parts of the population.

*Remarks*: We modified the character definition to more directly reflect that this character is essentially an absence vs. presence character, and not about a compositional question about how the foramen is formed. We retained the frequency information originally included by Meylan 1987 (character 98).

**Character 64 [character 66 in Lyson et al. 2021]**: Ilia curve medially. 1 = no; 2 = yes.

**Character 65 [character 67 in Lyson et al. 2021]**: Ischia extend into thyroid fenestra. 1 = yes; 2 = no.

**Character 66 [character 68 in Lyson et al. 2021]**: Metischial processes present and distinct. 1 = yes; 2 = no.

**Character 67 [character 69 in Lyson et al. 2021]**: Angle of acromion process to scapula approaches that of coracoid to acromion. 1 = no; 2 = yes.

**Character 68 [character 70 in Lyson et al. 2021]**: Coracoid longest of three pectoral processes. 1 = no; 2 = yes.

**Character 69 [character 71 in Lyson et al. 2021]**: Development of surface sculpturing of carapace and plastron. 0 = all metaplastic portions of carapace and plastron have trionychid sculpturing; 1 = trionychid pattern grades towards the center of carapacial and plastral disk to a smooth pattern, as developed in Hutchemys rememdium and Hutchemys arctochelys.

**Character 70 [character 72 in Lyson et al. 2021]**: Nuchal notch. 0 = anterior rim of nuchal convex or slightly notched; 1 = anterior rim of carapace with deep nuchal notch, as developed in H. rememdium.

**Character 71 [character 73 in Lyson et al. 2021]**: Shape of neural 1 and 2 (neural 2 and 3 of Meylan 1987). 0 = neurals 1 and 2 hexagonal with short posterior sides; 1 = neural 1 circular to rectangular and neural 2 octagonal, as developed in H. rememdium and H. arctochelys.

**Character 72 [character 74 in Lyson et al. 2021]**: Splitting of costals along distal margin. 0 = costal rim rounded or graded; 1 = dorsal rims split into separately protruding dorsal and visceral portions, as developed in H. rememdium and H. arctochelys.

**Character 73 [character 75 in Lyson et al. 2021]**: Lateral notch in carapace at the level of costal 5. 0 = absent, lateral carapacial margin rounded; 1 = present, lateral carapacial margin shows a waist, as developed in H. arctochelys.

**Character 74 [character 76 in Lyson et al. 2021]**: Skin callosity developed on the visceral side of costals 6 and 7. 0 = absent, visceral side smooth; 1 = present, visceral side sometimes develops a callosity, as seen in H. arctochelys.

**Character 75 [character 77 in Lyson et al. 2021]**: Mid-line contact of hyoplastra, hypoplastra and xiphiplastra. 0 = hyo-, hypo- and xiphiplastra do not contact another fully, even in adults; 1 = hyo-, hypo and xiphiplastra contact another fully along the entire mid-line in adults, as developed in H. rememdium and H. arctochelys.

**Character 76 [character 78 in Lyson et al. 2021]**: Shape of deep portion of entoplastron. 0 = lateral branches of entoplastron more or less straight and merge anterior at a clear angle; 1 = entoplastron wide and rounded, as seen in P. aff. thomasii.

**Character 77 [character 70 in Lyson et al. 2021]**: Mobility of entoplastron and anterior development of hyoplastron. 0 = lateral branches of entoplastron abut loosely against hyoplastron, anterior rim of hyoplastron develops no anterior flap/shoulder; 1 = lateral branches of entoplastron abut loosely against hyoplastron, but hyoplastron develops an anterior flap/shoulder, as seen in Plastomenus aff. thomasii2 = entoplastron tightly integrated into anterior plastral lobe due to strong development of anterior flap/shoulder, as developed in H. rememdium and H. arctochelys.

**Character 78 [character 80 in Lyson et al. 2021]**: Peripheral ossification. 0 = lateral bridge ossification of plastron does not significantly extend beyond the lateral processes of hyo- and hypoplastron; 1 = lateral bridge ossification of plastron extends laterally beyond bridge processes of the hyo- and hypoplastron and ossifies the peripheral aspects of the shell, as seen in H. arctochelys.

**Character 79 [character 81 in Lyson et al. 2021]**: Number of lateral hyoplastral processes. 1 = one; 2 = two; 3 = three or more.

**Character 80 [character 82 in Lyson et al. 2021]**: Extensive secondary palate consisting of infolded maxillae. 0 = absent; 1 = present, as developed in Plastomenus thomasii.

**Character 81 [character 83 in Lyson et al. 2021]**: Accessory ridges of upper triturating surfaces. 0 = absent; 1 = present.

**Character 82 [character 85 in Lyson et al. 2021]**: Dentary symphysis. 0 = short; 1 = extremely long, mandible extremely elongate, as developed in Plastomenus thomasii.

**Character 83 [character 86 in Lyson et al. 2021]**: Parietal contribution to orbits. 0 = parietals neither contribute to orbit margins or orbit walls; 1 = parietals either contribute to orbit walls, as developed in Gilmoremys lancensis, or orbit margin, as developed in Plastomenus thomasii.

**Character 84 [character 87 in Lyson et al. 2021]**: Proportions of costals VIII. 0 = wider than long to nearly square; 1 = significantly taller than wide.

**Character 85 [character 89 in Lyson et al. 2021]**: Hyoplastron with strongly serrated medial edge. 0 = present; 1 = absent.

**Character 86 [character 90 in Lyson et al. 2021]**: Serrated medial edge of hyoplastron extends nearly to the posterior edge of bone. 0 = present; 1 = absent.

**Character 87 [character 91 in Lyson et al. 2021]**: Process on medial edge of hypoplastron. 0 = of subequal size and radiating outward from the medial edge of the bone; 1 = with enlarged anterior process separated by a gap from smaller posterior processes.

**Character 88 [character 92 in Lyson et al. 2021]**: Ossification of basibranchials. 0 = poorly ossified, especially posterior pair ; 1 = well ossified.

**Character 89 [character 93 in Lyson et al. 2021]**: Metaplastic ossification of hypoplastron "rolls" onto posterior aspects of lateral processes. 0 = absent; 1 = present.

**Character 90 [character 94 in Lyson et al. 2021]**: Free rib ends. 0 = all ribs end free, not covered by metaplastic ossification; 1 = only costal ribs VII and VIII free2 = all ribs covered by metaplastic bone.

**Character 91 [character 95 in Lyson et al. 2021]**: Carapacial striations in adults. 0 = absent; 1 = present.

**NEW characters**

**Character 92 (This study).** Contribution of opisthotic to bar separating foramen jugulare posterius: 0 = absent; 1 = present.

**Character 93 (This study).** Contribution of excoccipital to bar separating foramen jugulare posterius: 0 = absent; 1 = present.

**Character 94 (This study).** Fusion of frontals: 0 = absent; 1 = present.

**Character 95 (Joyce 2007, ch15).** Jugal, jugal participation in the margin of the upper temporal emargination: 0 = absent; 1 = present, upper temporal emargination extensive.

**Character 96 (Evers et al. [2019]: character 30; Evers & Benson [2019]: character 29)**. Jugal, contact with the pterygoid: 0 = absent; 1 = present.

**Character 97 (This study).** Postorbital: 0 = completely reduced; 1 = present.

**Character 98 (This study).** Maxilla, well-developed suborbital crest: 0 = absent; 1 = present.

**Character 99 (This study).** Maxilla, Supraalveolar foramen (on the medial surface of the maxilla within the nasal capsule): 0 = absent; 1 = present, with posteriorly developed groove; 2 = present as fenestra-like opening into the supraalveolar canal.

**Character 100 (separate from character on foramen intermaxillaris, following Anquetin 2012, ch34).** Premaxilla/vomer, foramen praepalatinum: 0 = absent; 1 = present.

**Character 101 (separate from character on foramen intermaxillaris, following Anquetin 2012, ch34).** Foramina in vomer (possibly praepalatine foramina): 0 = absent; 1 = present.

**Character 102 (NEW, modified after Lyson & Joyce 2009).** Anterior palate region, vomer and or premaxillae/maxillae, transversely concave ventral surface (‘tongue groove’) on anterior parts between maxillae: 0 = absent; 1 = present.

**Character 103 (modified from Anquetin et al. 2015)***.* Quadrate, infolding ridge on the posterior surface of the quadrate ventral to the incisura columella auris: 0 = absent or extremely minor; 1 = ridge present but low; 2 = ridge present and forms massive overhanging flange. Ordered.

**Character 104 (Brinkman & Wu 1999, ch 49).** Pterygoid, pterygoid contribution to foramen palatinum posterius: 0 = absent; 1 = present.

**Character 105 (This study).** Maxilla, maxilla contribution to foramen palatinum posterius: 0 = absent; 1 = present.

**Character 106 (Joyce 2007, ch 42).** Pterygoid, lateral flange at lateral margin: 0 = absent; 1 = present.

**Character 107 (This study).** Parabasisphenoid, abducens nerve course: 0 = in fully ossified canal; 1 = in dorsally open groove.

**Character 108 (Evers & Benson 2019, ch 122).** Prootic, lateral semicircular canal enclosure by bone: 0 = canal only formed by bone of the opisthotic, the prootic portion of the canal is not ossified and is instead medially confluent with the recessus labyrinthicus prooticus; 1 = prootic and opisthotic both contribute to the formation of the lateral semicircular canal.

**Character 109 (modified from Zhou & Rabi 2015, ch 253; as in Evers & Benson 2019, ch 148).** Position of the foramen posterius canalis carotici interni (fpcci): 0 = the fpcci is located at the ventral surface of the skull in a position far anterior to the margin of the fenestra postotica; 1 = the fpcci is located at the posterior end of the skull, either on the ventral surface of the skull close to the margin of the fenestra postotica, or on the posterior surface of the skull at the ventral margin of the fenestra postotica.

***Character 110 (modified from Evers et al. 2022, ch 8).** Dentary, expansion of lingual margin: 0 = absent; 1 = lingual margin is medially expanded to a shelf, which often is broader along the central part of the mandibular ramus than near the coronoid and symphyseal ends; 2 = lingual margin is so far expanded that it forms a huge, spatulate continuous surface with the symphysis. Ordered.

***Character 111 (modified from Hirayama 1985, ch 47; as in Evers et al. 2022, ch 16).** Dentary, size of foramen dentofaciale majus: 0 = absent, foramen completely reduced; 1 = small, size of a small vessel; 2 = enlarged, foramen is several mm in diameter. Ordered

**Character 111 (Evers et al. 2022, ch 22).** Dorsal surangular foramen: 0 = absent; 1 = present.

**Character 112 (Evers & Benson 2019, ch 182).** Coronoid, foramen at anterior end, leading from fossa Meckelii into space between mandibular rami: 0 = absent; 1 = present.

**Character 113 (Evers et al. 2022, ch 34).** Prearticular, exposure of foramen alveolare inferius in medial view: 0 = absent, the foramen is covered by expansions of the prearticular and/or coronoid; 1 = present, the foramen can be seen in medial view.

**Character 114 (modified from Vlachos & Rabi 2019, ch 68; as in Evers et al. 2022, ch 37).** Anterior intermandibular foramen: 0 = absent; 1 = present, fully or partially formed between the angular and prearticular along their anterior processes. Note that this character is scored as inapplicable when the anteroventral process of the prearticular is absent.

**Character 115 (modified from Meylan 1996, ch 21; as in Evers et al. 2022, ch 46).** Posterior chorda tympani foramen: 0 = located within the articular notch on the posterior surface of the articular; 1 = located on the posteromedial jaw surface, usually on the prearticular or prearticular-articular contact.

**Character 116 (Evers et al. [2022] : character 51).** Articulation facet, contribution of prearticular: 0 = absent, prearticular completely retracted from articulation facet; 1 = prearticular forms small medial portion of articulation facet.

**DELETED CHARACTERS :**

Character 56: Quadratojugal participates in processus trochlearis oticum. 1 = no; 2 = yes.

*Remarks*: We could not reproduce the scorings for this character and do not properly understand the variation that is underlying this character, and thus removed it from the matrix.

Character 84: Posterior portion of narial canal defined by bone. 0 = absent; 1 = present, as developed in *Gilmoremys lancensis*.

*Remarks*: We could not reproduce the scorings for this character and do not properly understand the variation that is underlying this character, and thus removed it from the matrix.

Character 88: Ilium with expanded dorsal end. 0 = present; 1 = absent.

*Remarks*: We could not reproduce the scorings for this character and do not properly understand the variation that is underlying this character, and thus removed it from the matrix.

Character 38: Foramen palatinum posterius forms in. 1 = palatine and pterygoid and/or maxilla; 2 = palatine only.

*Remarks*: As the palatine is always involved in the formation of the foramen palatinum posterius, but as there is variation as to whether the pterygoid or maxilla contribute to the formation of the foramen, we deleted this character and instead add two new one, which each score the absence vs. presence of the maxilla or pterygoid contribution to the foramen, respectively.

**References**

Anquetin, J., Püntener, C., Billon-Bruyat, J.-P. (2015). *Portlandemys gracilis* n. sp., a new coastal marine turtle from the late Jurassic of Porrentruy (Switzerland) and a reconsideration of plesiochelyid cranial anatomy. *PLOS ONE,* *10(6)*, e0129193. doi: [10.1371/journal.pone.0129193](https://doi.org/10.1371/journal.pone.0129193)

Brinkman, D., Wu, X.-C. (1999). The skull of *Ordosemys*, an Early Cretaceous turtle from Inner Mongolia, People’s Republic of China, and the interrelationships of Eucryptodira (Chelonia, Cryptodira). *Paludicola, 2(2)*, 134–147.

Evers, S.W., Benson, R.B.J. (2019). A new phylogenetic hypothesis of turtles with implications for the timing and number of evolutionary transitions to marine lifestyles in the group. *Palaeontology, 62(1)*, 93–134.

Evers, S.W., Barrett, P.M., Benson, R.B.J. (2019). Anatomy of *Rhinochelys pulchriceps* (Protostegidae) and marine adaptation during the early evolution of chelonioids. *PeerJ, 7,* e6811. doi: [doi.org/10.7717/peerj.6811](https://doi.org/10.7717/peerj.6811)

Evers, S.W., Ponstein, J., Gray, J., Jansen, M., Fröbisch, J. (2022a). A systematic compendium of turtle mandibular anatomy using digital dissections of soft tissue and osteology. *Anatomical Record,* 1–76. doi: [doi.org/10.1002/ar.25037](https://doi.org/10.1002/ar.25037)

Hirayama, R. (1985). Cladistic analysis of batagurine turtles. *Studia Palaeocheloniologica, 1*, 140–157.

Joyce, W.G. (2007). Phylogenetic relationships of Mesozoic turtles. *Bulletin of the Peabody Museum of Natural History,* *48*, 3–102.

Lyson, T.R., Joyce W.G. (2007). A revision of *Plesiobaena* (Testudines: Baenidae) and an assessment of baenid ecology across the K/Pg boundary. *Journal of Paleontology, 83(6)*, 833­–853.

Lyson, T.R., Petermann, H., Miller, I.M. (2021). A new plastomenid trionychid turtle, Plastomenus joycei, sp. nov., from the earliest Paleocene (Danian) Denver Formation of south-central Colorado, U.S.A. *Journal of Vertebrate Paleontology,* *41(1)*, 1913600. doi: [doi.org/10.1080/02724634.2021.1913600](https://doi.org/10.1080/02724634.2021.1913600)

Meylan, P.A. (1987). The phylogenetic relationships of soft-shelled turtles (Family Trionychidae). *Bulletin of the American Museum of Natural History,* *186(1)*, 1–110.

Meylan, P.A. (1996). Skeletal morphology and relationships of the Early Cretaceous Side-Necked turtle, *Araripemys barretoi* (Testudines: Pelomedusoides: Araripemydidae), from the Santana Formation of Brazil. *Journal of Vertebrate Paleontology,* *16(1)*, 20–33.

Vlachos, E., Rabi, M. (2018). Total evidence analysis and body size evolution of extant and extinct tortoises (Testudines: Cryp- todira: Pan-Testudinidae). *Cladistics,* *34*, 652–683.

Zhou, C.-F., Rabi, M. (2015). A sinemydid turtle from the Jehol Biota provides insights into the basal divergence of crown turtles. *Scientific Reports,* *5*, 16299. doi: doi.org/10.1038/srep16299
